# Recomendaciones preventivas vasculares. Actualización PAPPS 2024

**DOI:** 10.1016/j.aprim.2024.103123

**Published:** 2024-11-27

**Authors:** Domingo Orozco-Beltrán, Carlos Brotons-Cuixart, José R. Banegas, Vicente F. Gil-Guillen, Ana M. Cebrián-Cuenca, Enrique Martín-Rioboó, Ariana Jordá-Baldó, Johanna Vicuña, Jorge Navarro-Pérez

**Affiliations:** a Medicina Familiar y Comunitaria, Unidad de Investigación CS Cabo Huertas, Departamento San Juan de Alicante. Departamento de Medicina Clínica. Centro de Investigación en Atención Primaria. Universidad Miguel Hernández, San Juan de Alicante, España; b Medicina Familiar y Comunitaria. Institut de Recerca Sant Pau (IR SANT PAU). Equipo de Atención Primaria Sardenya, Barcelona, España; c Medicina Preventiva y Salud Pública, Universidad Autónoma de Madrid y CIBERESP, Madrid, España; d Medicina Familiar y Comunitaria. Hospital Universitario de Elda. Departamento de Medicina Clínica. Centro de Investigación en Atención Primaria. Universidad Miguel Hernández, San Juan de Alicante, España; e Medicina Familiar y Comunitaria, Centro de Salud Cartagena Casco Antiguo, Cartagena, Murcia, España. Instituto de Investigación Biomédica de Murcia (IMIB), Universidad Católica de Murcia, Murcia, España; f Medicina Familiar y Comunitaria, Centro de Salud Poniente, Córdoba. Departamento de Medicina. Universidad de Córdoba. Grupo PAPPS, Córdoba, España; g Medicina Familiar y Comunitaria. Centro de Salud Plasencia II, Plasencia, Cáceres, España; h Medicina Preventiva y Salud Pública. Hospital de la Sant Creu i Sant Pau, Barcelona, España; i Medicina Familiar y Comunitaria, Centro de Salud Salvador Pau (Valencia). Departamento de Medicina. Universidad de Valencia. Instituto de Investigación INCLIVA, Valencia, España

**Keywords:** Enfermedades cardiovasculares, Diabetes mellitus, Obesidad, Hipertensión arterial, Dislipemias, Atención primaria ;, Cardiovascular Diseases, Diabetes Mellitus, Obesity, Hypertension, Dyslipidemias, Primary Health Care

## Abstract

Se presentan las recomendaciones del Programa de Actividades Preventivas y Promoción de la Salud (PAPPS) de la Sociedad Española de Medicina de Familia y Comunitaria (semFYC), para la prevención de las enfermedades vasculares (EV). Como novedad en esta edición se incluyen nuevos apartados como obesidad, enfermedad renal crónica (ERC) y esteatosis hepática metabólica; además de un apartado «No hacer» en las diferentes patologías tratadas. Se han actualizado los apartados: Revisión epidemiológica, donde se describe la morbimortalidad vascular actual en España y su evolución y los principales factores de riesgo, riesgo vascular (RV) y recomendaciones para el cálculo del RV, factores de riesgo mayores como hipertensión arterial (HTA), dislipidemia y diabetes mellitus (DM), describiendo el método para su diagnóstico, los objetivos terapéuticos y las recomendaciones de medidas de estilo de vida y de tratamiento farmacológico, indicaciones de antiagregación y recomendaciones para el cribado de la fibrilación auricular (FA), así como para el manejo del paciente crónico. Para las principales recomendaciones se incluyen tablas específicas que recogen la calidad de la evidencia y la fuerza de la recomendación.

## Abstract (en)

The recommendations of the semFYC's Program for Preventive Activities and Health Promotion (PAPPS) for the prevention of vascular diseases (VD) are presented. New in this edition are new sections such as obesity, chronic kidney disease and metabolic hepatic steatosis, as well as a ‘Don’t Do’ section in the different pathologies treated. The sections have been updated: epidemiological review, where the current morbidity and mortality of CVD in Spain and its evolution as well as the main risk factors are described; vascular risk (VR) and recommendations for the calculation of CV risk; main risk factors such as arterial hypertension, dyslipidemia and diabetes mellitus, describing the method for their diagnosis, therapeutic objectives and recommendations for lifestyle measures and pharmacological treatment; indications for antiplatelet therapy, and recommendations for screening of atrial fibrillation, and recommendations for management of chronic conditions. The quality of testing and the strength of the recommendation are included in the main recommendations.

## Revisión epidemiológica y riesgo vascular

### Mortalidad vascular

En el 2022, 121.341 personas murieron en España por enfermedades vasculares (EV), que siguen siendo la primera causa de muerte (26,1%) (+1,8% vs. 2020), seguida de los tumores (24,7%) (+1,9% vs. 2020). Las enfermedades infecciosas siguen ocupando el tercer puesto (9,3%) (-7,1% vs. 2020). Sin embargo, dentro del conjunto de causas, la mortalidad por EV se ha reducido, del 34,9% en el año 2000 (30,1% en hombres y 40,2% en mujeres) al 24,3% en 2022 (24,4% en hombres y 27,9% en mujeres)^1^.

En el año 2022 la enfermedad isquémica representaba el 12,7% y las enfermedades cerebrovasculares, el 20,3% del total de la mortalidad^1^. La preponderancia de la enfermedad cerebrovascular sobre la enfermedad isquémica del corazón se produce a expensas de las mujeres (56,1% vs. 38,0%), dato que no ocurre en los varones (43,9% vs. 62,0%)^1^.

El riesgo de morir (tasas ajustadas por edad) por las EV está disminuyendo en España desde mediados de la década de 1970, sobre todo debido al descenso de la mortalidad por enfermedades cerebrovasculares y coronarias. Sin embargo, en el contexto de la pandemia por COVID-19, se ha observado que la mortalidad por EV aumentó un 2,9% respecto a 2019, siendo esta más elevada en mujeres que en hombres^2^. Además, destaca el incremento considerable de muertes por enfermedades hipertensivas entre el 2019 y el 2020, siendo en esa fecha el doble que en el 2006. La tasa estandarizada de mortalidad por EV fue de 219,4/10^5^ habitantes en el 2020, pero con una importante diferencia entre comunidades autónomas (Madrid 173,2/105 vs. Andalucía 282,3/105)^2^.

En el ámbito internacional, las tasas de mortalidad ajustadas por edad de España para el conjunto de las enfermedades del sistema circulatorio, la enfermedad isquémica del corazón y la enfermedad cerebrovascular son más bajas que en otros países occidentales^3^, ^4^, ^5^.

#### Morbilidad vascular

En el año 2020, en España, la tasa de morbilidad hospitalaria de las enfermedades del sistema circulatorio fue de 1.285 por 10^5^ hab. (1.285 en varones y 935 en mujeres), y causó 4,5 millones de estancias hospitalarias y 524.016 altas^1^. La tasa de morbilidad hospitalaria de la enfermedad isquémica del corazón fue de 249 por 10^5^ hab. (366 en los varones y 138 en las mujeres). Respecto a la enfermedad cerebrovascular, la tasa de morbilidad fue de 237 por 10^5^ hab. (260 en varones y 215 en mujeres)^1^. Por tanto, se observan tasas de morbilidad vascular hospitalaria superiores en hombres que en mujeres. La tendencia de las tasas de morbilidad hospitalaria de las enfermedades del sistema circulatorio ha sido de un constante aumento desde 1977 hasta 2019, con un ligero descenso en el periodo 2003-2012, tras lo cual disminuyó 13,2 puntos en el 2020^1^ probablemente debido a la pandemia por COVID-19.

#### Mortalidad prematura y años potenciales de vida perdidos

El cáncer pasó a ser la primera causa de mortalidad prematura en ambos sexos en los países de ingreso alto como España, relegando a la segunda posición a las EV, las cuales siguen en el primer puesto en los países europeos de ingreso medio^4^. Un aspecto que merece destacar es que las EV siguen siendo la primera causa de mortalidad prematura (< 70 años) en hombres en toda Europa, mientras que en las mujeres es el cáncer^4^.

Del total de años potenciales de vida perdidos, las EV supusieron en Europa el 35% en hombres y el 40% en mujeres, siendo mayor que el cáncer que supuso 24% en hombres y 25% en mujeres^4^.

### Factores de riesgo vascular

Los factores de riesgo vascular (FRV) mayores, causales de enfermedad y mortalidad, incluyen la HTA, la dislipemia, la diabetes mellitus (DM), el tabaquismo y la obesidad^2^, ^4^, ^5^. Además, existen factores adicionales como el consumo de alcohol, el sedentarismo y una dieta no saludable, así como factores que potencialmente pueden modificar el cálculo del riesgo vascular (RV) (ver apartado «Tablas de riesgo vascular»)^5^.

#### Hipertensión arterial

En todos los grupos de edad y étnicos, la presión arterial (PA) elevada tiene una relación continua e independiente con la incidencia de ictus hemorrágico, ictus isquémico, infarto agudo de miocardio (IAM), muerte súbita, enfermedad arterial periférica, enfermedad renal terminal, fibrilación auricular (FA), deterioro cognitivo y demencia^6^, ^7^, ^8^, ^9^. La presión arterial diastólica (PAD) se asocia a un aumento de EV en menores de 50 años, a partir de cuya edad la presión arterial sistólica (PAS) y en especial valores ≥ 140 mmHg, pasa a ser el mejor indicador de complicaciones por EV y discapacidad. Además, la PA superior a > 120/80 mmHg, incluyendo por tanto a la HTA, fue el factor de riesgo con una mayor asociación con la mortalidad prematura en Europa^6^, ^7^, ^8^, ^9^.

#### Dislipemia

La relación causal entre los niveles de colesterol unido a lipoproteínas de baja densidad (c-LDL) y el RV ha sido establecido a través de diferentes estudios genéticos, observacionales y experimentales^5^, en los que se observa que la reducción de los valores de c-LDL disminuye el RV. Además, en personas con RV muy alto y alto, este beneficio aparece con cualquier reducción en los niveles de c-LDL. Otros indicadores que evalúan los lípidos no- lipoproteínas de alta densidad (HDL) (apoB, colesterol no-HDL), demostraron tener una relación tan fuerte como la del c-LDL con el RV^5^. Sin embargo, no hay evidencia de que aumentar los niveles de colesterol HDL (c-HDL) disminuya el RV, pero sí que son un indicador útil para el cálculo del RV según la escala *Systematic Coronary Risk Evaluation-2* (SCORE2) (ver apartado «Tablas de riesgo vascular»).

#### Diabetes mellitus tipo 2

Tanto la DM tipo 1 (DM1) como la DM tipo 2 (DM2) son factores de riesgo independientes de EV aterosclerótica, duplicando el riesgo. Las mujeres con diabetes tienen un riesgo mayor al de los hombres. Además, son pacientes que suelen tener asociado más de un FRV, como por ejemplo la hipertensión la dislipidemia o la obesidad^5^.

#### Tabaquismo

El tabaquismo es responsable del 50% de las muertes evitables en fumadores, siendo la mitad de ellas por enfermedad aterosclerótica. Además, causa en promedio la pérdida de 10 años de vida en las personas que han fumado durante toda su vida. El RV en personas fumadoras de menos de 50 años de edad es cinco veces superior al de no fumadores, y afecta más a las mujeres que a los hombres^5^, ^8^, ^9^, ^10^, ^11^, ^12^.

En el mundo, tras la PA elevada, el tabaquismo es el principal factor de riesgo en cuanto a DALYs (o los años de vida ajustados por discapacidad [AVAD]), que son años perdidos debido a muertes prematuras o vividas con discapacidad. Cabe destacar que los fumadores pasivos también tienen un RV superior al de los no fumadores, y que los fumadores de cigarrillos electrónicos también tienen un RV aumentado^5^, ^8^, ^9^, ^10^, ^11^, ^12^.

#### Obesidad

A nivel mundial, el índice de masa corporal (IMC en kg/m^2^) incrementó considerablemente en los niños, adolescentes y adultos en las últimas décadas, teniendo una prevalencia superior a la del bajo peso a nivel global. En cuanto al RV, el metaanálisis del *Global* *BMI* *Mortality Collaboration* evidencia que el *Hazard ratio* (HR) de mortalidad fue de 1,39 (intervalo de confianza [IC] 95%: 1,34-1,43) para el aumento de cada 5 kg/m^2^ en personas con sobrepeso u obesidad. Existe una asociación continua entre el IMC y la mortalidad. Tanto el IMC y sobre todo la circunferencia de la cintura, se asocian fuertemente y de forma continua con la EV aterosclerótica y la DM2^5^.

#### Alcohol

La asociación del consumo de alcohol con las EV es incierta, pero en los estudios que lo abordan, se encontró una asociación positiva con la mortalidad por todas las causas, con un umbral de menor riesgo de 100 g/semana (5,2 L/año de alcohol puro), concretamente para los accidentes cerebrovasculares y la insuficiencia cardiaca. Se ha estimado que la esperanza de vida de un bebedor de 40 años podría incrementarse hasta en dos años mediante reducciones en el consumo de alcohol por debajo de 100 g/semana^4^.

La Unión Europea es la región del mundo con mayor consumo excesivo de alcohol y tiene la proporción más alta de enfermedades totales y muertes prematuras atribuibles al alcohol. En el año 2020, España ocupó el cuarto puesto en hombres (20,1 L/año) y el noveno en mujeres (5,8 L/año) en cuanto a consumo de alcohol en Europa^4^.

#### Sexo y género

La Organización Mundial de la Salud (OMS) define el sexo como las características biológicas de mujeres, hombres y personas intersexuales^13^. Los modelos de medicina cardiovascular han utilizado mayormente muestras masculinas, por lo que es vital comprender cómo la fisiología específica del sexo afecta el desarrollo y curso de las EV^14^. Las EV son la principal causa de muerte en mujeres en países desarrollados, aunque su tasa de mortalidad es menor que la de los hombres en todos los grupos de edad. El RV es similar en ambos sexos, pero las mujeres experimentan eventos cardiovasculares más tardíamente^15^. Factores como menarquia temprana, menopausia prematura y complicaciones del embarazo incrementan el RV en mujeres^5^, ^15^, ^16^. A pesar de ello, estas condiciones no modifican el cálculo de RV ni reclasifican a los pacientes.

El género, definido por la OMS como características socialmente construidas^13^, también influye en la salud vascular, interactuando con otros determinantes sociales como la etnicidad y la posición socioeconómica (tabla 1)^14^. En países con alta igualdad de género, se han observado grandes reducciones en la mortalidad por cardiopatía coronaria a 40 años^14^. Sin embargo, existen diferencias en la atención médica entre hombres y mujeres debido a sesgos y la percepción errónea de que las EV son problemas masculinos, lo que lleva a un cribado y manejo inadecuado en mujeres^15^. Por ejemplo, un estudio en EE. UU. observó que la supervivencia de las mujeres tratadas por médicos hombres es menor que la de los hombres^17^.

**Tabla 1:** Efecto del género en la enfermedad vascular

Conciencia del riesgo	Menor conciencia y conocimiento del riesgo en las mujeres
Actividad física	Tendencia a que los niños desarrollen fuerza física y que las niñas desarrollen habilidades emocionales y verbales. El sedentarismo es mayor en las mujeres desde los seis años de edad
Sociabilización	El aislamiento social es un factor de riesgo vascular potente, influyendo en el consumo de drogas ilícitas que aumentan el RV. Los roles y rasgos de género (masculinidad en particular) explican parte de las diferencias de género en el estrés y el afrontamiento. Los datos del *Framingham Offspring Study* muestran que las medidas de ira y hostilidad predicen el desarrollo de la fibrilación auricular en los hombres
Tabaquismo	Las tasas de tabaquismo son similares entre hombres y mujeres en los países de altos ingresos. Las mujeres que empiezan a fumar a partir de los 16 años son más propensas a desarrollar hipertensión y enfermedad cardiaca. Gran parte del hábito tabáquico en las mujeres (46%) y en los hombres (30%) está relacionado con el control del peso para el mantenimiento de la imagen corporal.
Eventos traumáticos	Los eventos adversos en la infancia son predictores sólidos de problemas vasculares en la vejez, incluida la aparición y recurrencia de EV, aunque del 50% al 80% de esta relación está mediada por factores de RV tradicionales. De manera similar, la victimización por violencia de pareja íntima en la edad adulta se ha asociado con comportamientos y resultados de RV nocivos, siendo las mujeres las más susceptibles a este fenómeno.
Estrés laboral, doméstico y financiero	El acoso diario y la discriminación son estresores crónicos que comprometen la salud vascular. Esto afecta aún más a las mujeres no caucásicas, a las minorías religiosas y a las personas de orientación no-heterosexual. A menudo se socializa a los niños desde una edad temprana para que crean que son financieramente responsables de una familia, mientras que es más probable que las niñas sean socializadas para que sean emocionalmente responsables. En hogares con mayor equidad de género, social o financiera, se observó una mejoría del estado global de salud.

Elaboración propia. Modificado de ^11,13^.

La evidencia existente sobre el efecto modificador del riesgo del sexo, de condiciones clínicas específicas del sexo y de estrategias de manejo clínico, está incluida en las Guías Europeas de Prevención Cardiovascular del 2021. Esta última recalca también la importancia de la influencia del género en la experiencia de un individuo en cuanto a acceso a la atención médica, y su impacto en la salud^5^.

## Estimación del riesgo de eventos vasculares

### Tablas de riesgo vascular

En las Guías Europeas de Prevención Vascular 2021^5^ se recomienda el cálculo del RV de manera sistemática a todas las personas adultas con algún factor de RV, pudiéndose considerar también en los hombres > 40 años y las mujeres > 50 años, y que se pueda repetir cada cinco años.

Las guías presentan un modelo para calcular el riesgo SCORE2^18^ y *Systematic Coronary Risk Evaluation-2 Old person* (SCORE2-OP)^19^, que ha sido calibrado para cuatro regiones de Europa (bajo, moderado, alto y muy alto) según las tasas de mortalidad vascular, perteneciendo España a los países de bajo RV.

Esta herramienta permite el cálculo del riesgo de morbimortalidad vascular en los próximos 10 años (IAM, ictus y mortalidad vascular) en hombres y mujeres entre 40 y 89 años. Se pueden usar las tablas coloreadas que aparecen en las guías (utilizan PAS, edad, sexo, tabaco y colesterol no-HDL) o bien la App de la *European Society of Cardiology* (ESC) o la herramienta disponible en la web que permite la entrada del colesterol total y c-HDL^20^. Mediante estas aplicaciones es posible calcular también el RV de por vida (*LIFE-CV model*) y los beneficios del tratamiento en términos de años de vida ganados sin EV. Además, hay herramientas específicas para cálculo del riesgo en personas con EV establecida (*SMART RISK score o SMART REACH model*). Recientemente se han publicado modelos específicos para diabetes^21^ y para insuficiencia renal crónica^22^.

Estas herramientas estiman el riesgo en pacientes que no están en tratamiento antihipertensivo o hipolipemiante o también en aquellos en tratamiento estable por varios años. La derivación del SCORE2 se hizo a partir de 45 cohortes de 11 países europeos, de EE. UU. y Canadá (casi 70.000 individuos) entre 1990 y 2009. De España participaron cohortes de estudio Dieta y riesgo de enfermedad cardiovascular en España (DRECE)^23^ y estudio Zaragoza^24^. Para la validación externa se utilizaron 25 cohortes de 15 países europeos (más de 1,1 millones de individuos). De España se utilizaron cohortes del estudio *Multinational Monitoring of Trends and Determinants in Cardiovascular Disease* (MONICA) Cataluña II^25^, ^26^ y del estudio *European Prospective Investigation into Cancer and Nutrition and Cardiovascular Disease* (EPIC-CVD)^27^*.*

También se hizo la recalibración para cuatro regiones de Europa utilizando datos de mortalidad vascular de la OMS, aplicando unos factores multiplicativos extraídos de datos de cohortes y registros, calculando la incidencia de eventos fatales y no fatales. Un riesgo de un 6% en un país de bajo riesgo equivale a un riesgo de un 14% en un país de muy alto riesgo.

#### Categorías de riesgo vascular según SCORE2/SCORE2-OP en personas aparentemente sanas

Se establecen diferentes umbrales de riesgo dependiendo de la edad, como se muestra en la tabla 2, a diferencia de las versiones anteriores, que establecían un único umbral de riesgo, al objeto de evitar el infratratamiento en jóvenes y el sobretratamiento en mayores, ya que el beneficio a largo plazo del tratamiento de los FRV es mayor en pacientes jóvenes. En la tabla 3 se presenta la definición de las categorías de riesgo.

**Tabla 2:** Categorías de riesgo vascular según grupos de edad*

	< 50 años	50-69 años	≥ 70 años
Bajo a moderado riesgo vascular	< 2,5%	< 5%	< 7,5%
Alto riesgo vascular	2,5 a < 7,5%	5 a < 10%	7,5 a < 15%
Muy alto riesgo vascular	≥ 7,5%	≥ 10%	≥ 15%

* Riesgo en los próximos 10 años. Modificada de ^5^.

**Tabla 3:** Definición de las categorías de riesgo

Riesgo muy alto	**• Enfermedad vascular documentada**, ya sea clínicamente o a través de imágenes, incluyendo infarto de miocardio, síndrome coronario agudo, revascularización coronaria o de otras arterias, ictus y accidente vascular transitorio, y enfermedad vascular periférica, aneurisma de aorta, así como la presencia de placas en la arteriografía coronaria o en la ecografía carotídea. No incluiría aumento del grosor de la íntima media carotídea **•** Pacientes con **DM con enfermedad vascular o con lesión severa de órgano diana,** la presencia de filtrado glomerular estimado (FGe) < 45 mL/min/1,73 m^2^ o FGe 45-59 mL/min/1,73 m^2^ e índice albúmina/creatinina (IAC) 30-300 mg/g o IAC > 300 mg/g; de igual manera la presencia de complicaciones microvasculares en tres sitios diferentes (MA, retinopatía, neuropatía), confiriendo una situación de muy alto RV (afectación órgano diana grave) **• Enfermedad renal crónica (ERC) grave,** FGe < 30 ml/min/1,73m^2^ o FGe 30-44 ml/min/1,73m^2^ e índice albúmina/creatinina (IAC) > 30 mg/g o FGe 45-59 ml/min/1,73 m^2^ e IAC > 300 mg/g (Fig. 4) **• Puntuación SCORE2** ≥ 7,5% (< 50 años), ≥ 10% (50-69 años), ≥ 15% (≥ 70 años).
Riesgo alto	**• Enfermedad Renal Crónica (ERC) moderada,** FGe 30-44 ml/min/1,73m2 e IAC < 30 mg/g o FGe 45-59 ml/min/1,73 m2 e IAC 30-300 mg/g o FGe >60 ml/min/1,73 m2 e IAC >300 mg/g. **• Valores muy elevados de colesterol** (> 8 mmol/l (310 mg/dl), **o de PA** > 180/110 mmHg **•** Pacientes con **DM sin enfermedad vascular ni afectación de órganos diana** que no cumplan criterios de riesgo moderado. **•** Pacientes con **hipercolesterolemia familiar** **• Puntuación SCORE2** 2.5 - < 7.5% (< 50 años), 5 - < 10% (50-69 años), 7,5 - < 15% (70 años).
Riesgo de bajo a moderado	**•** Pacientes con **DM bien controlados de menos de 10 años de evolución**, sin afectación de órganos diana ni la presencia de otros factores de riesgo. **• Puntuación SCORE2** < 2.5% (< 50 años), < 5% (50-69 años), < 7,5% (70 años).

Se recomienda tratar a toda persona de muy alto riesgo y debería considerarse el tratamiento para las de alto riesgo, en función de los modificadores de riesgo, el riesgo de por vida, los beneficios del tratamiento y las preferencias personales.

**Figura 1:**
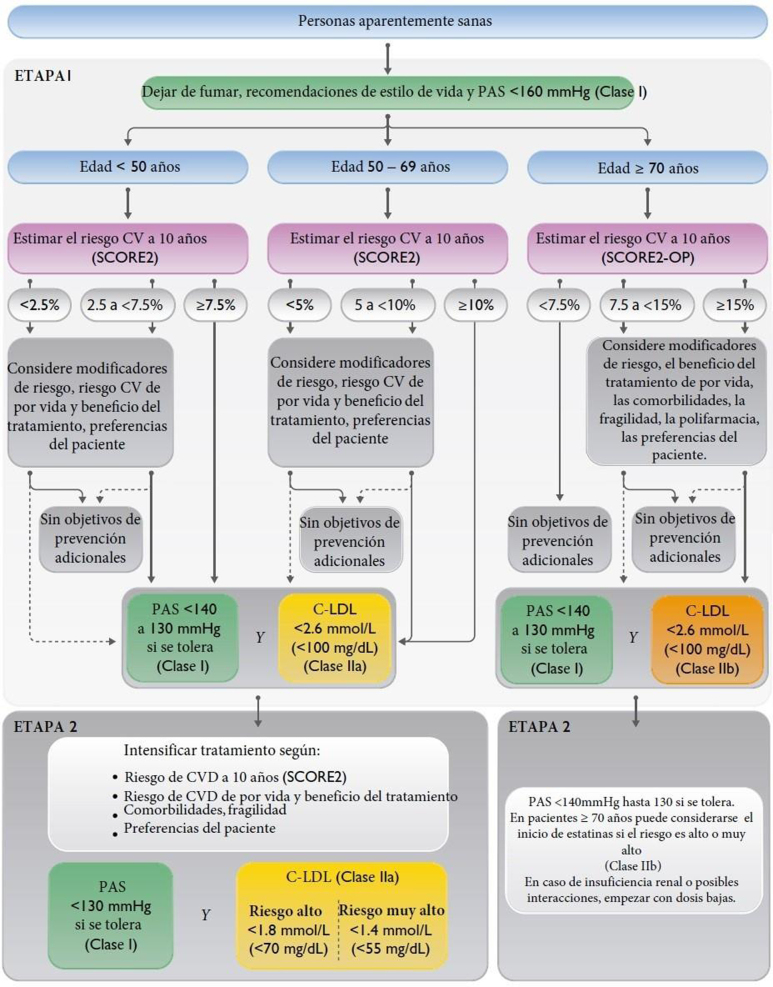
Cálculo del RV y tratamiento de los factores de RV para personas aparentemente sanas. Modificado de ^5^.

**Figura 2:**
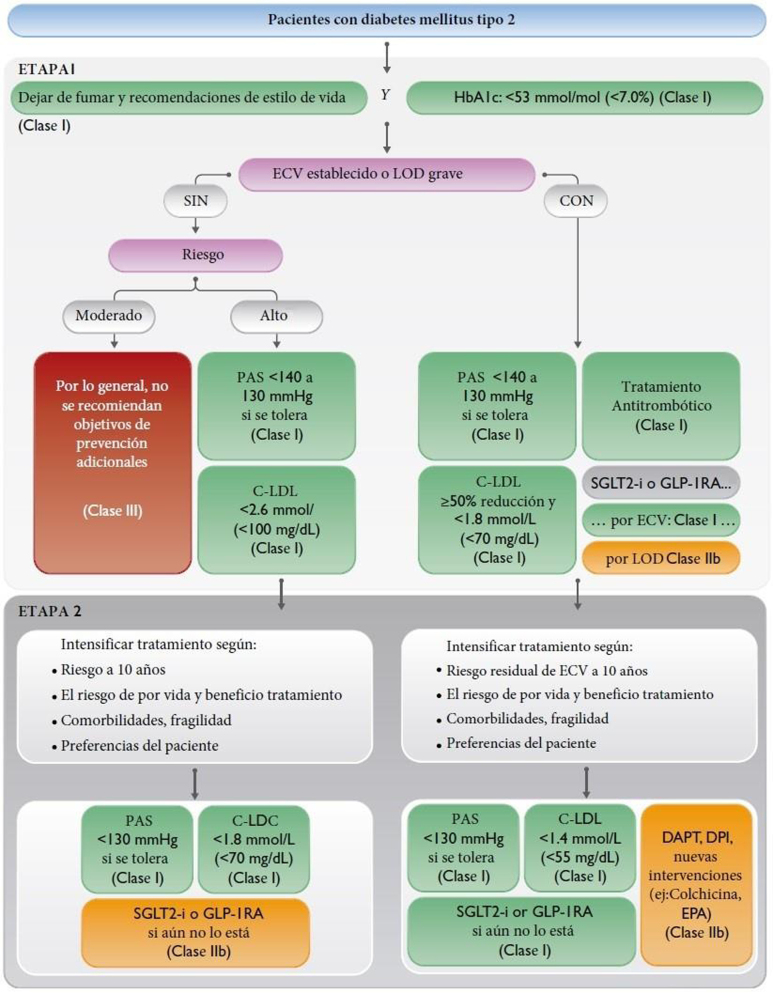
Cálculo del RV y tratamiento de los factores de RV para personas con diabetes mellitus tipo 2. Modificado de ^5^.

**Figura 3:**
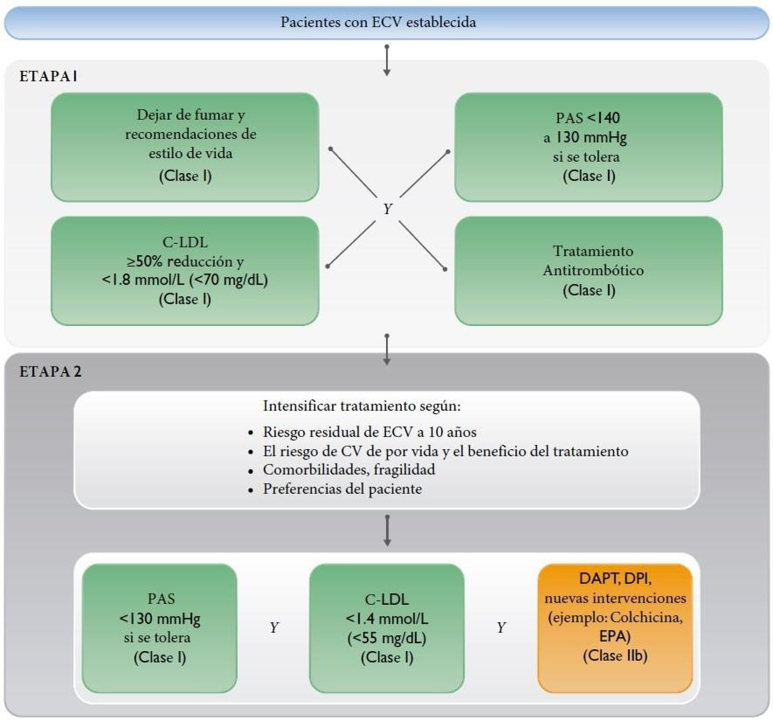
Cálculo del RV y tratamiento de los factores de RV para personas con enfermedad vascular aterosclerótica. Modificado de ^5^.

En las Figura 1, Figura 2, Figura 3 se exponen los algoritmos de cálculo del RV y tratamiento de los FRV para personas aparentemente sanas, pacientes con diabetes y pacientes con EV aterosclerótica. Los pacientes con enfermedad renal crónica (ERC) se considerarán de riesgo alto o muy alto según la tasa del filtrado glomerular (FG) y el índice albúmina/creatinina (IAC). Los pacientes con hipercolesterolemia familiar se consideran de riesgo alto.

A destacar que en los pacientes igual o mayores de 70 años la evidencia de iniciar estatinas es más incierta, por lo que en estos pacientes, que fácilmente son de alto o muy alto riesgo, hay que tener en cuenta otros factores como la insuficiencia renal o las posibles interacciones con otros fármacos, y mejor empezar con dosis bajas.

Se resalta la importancia de la comunicación con el paciente, recomendándose tener una discusión informada sobre el riesgo y los beneficios terapéuticos, adaptados a las necesidades del individuo. Concretamente, se comenta la necesidad de utilizar el RV de por vida, sobre todo en los más jóvenes, o los beneficios de por vida tras la intervención o la edad vascular.

##### Factores modificadores del riesgo

Los factores que potencialmente pueden modificar el cálculo del RV deben cumplir criterios específicos para ser considerados como tales: mejoran la discriminación o reclasifican, tienen impacto sobre la salud pública, su medición es factible en la práctica diaria, se conoce como aumenta y disminuye el riesgo según la presencia o ausencia del factor de riesgo, y no existe sesgo de publicación en la literatura (tabla 4).

**Tabla 4:** Modificadores del riesgo

	Explicación
Estrés psicológico	Tiene efectos biológicos directos, pero también dependen del nivel socioeconómico y de factores de riesgo conductuales. El riesgo vascular aumenta con el estrés, desde la aparición de síntomas de estrés hasta eventos trágicos de la vida (RR 1,2 a 2,0). Existe un posible efecto favorable del cribado y tratamiento de la depresión en la reducción de eventos vasculares mayores
Etnia	En España, y el resto de Europa, hay grupos étnicos distintos resultantes de procesos migratorios. Los riesgos vasculares pueden variar de una etnia a otra, por lo que las calculadoras de riesgo vascular deberían ser corregidas por este factor. Cada país tendría que individualizar su propio *risk-score* de acuerdo a su población. Por ahora, esto solo se hizo en Reino Unido, en donde se corrigen los riesgos relativos al alza en asiáticos (excepto China), y a la baja en afrodescendientes y chinos
Fragilidad	Es un estado independiente de la edad y morbilidad que hace que una persona sea vulnerable al efecto de estresores. La capacidad de las medidas de fragilidad en mejorar la predicción del riesgo no se ha evaluado formalmente, por lo que no se recomienda su integración en la evaluación formal del riesgo de enfermedad CV. Se sugiere tener en cuenta este factor para el ajuste terapéutico individual
Historia familiar	Es una información simple y fácil de recoger. Los antecedentes familiares de enfermedad vascular prematura solo mejoran marginalmente la predicción del riesgo de enfermedad CV más allá de los factores de riesgo convencionales
Genética	Existe una falta de consenso sobre la utilidad de la genética y sobre la elección del marcador. La precisión de la predicción incremental es relativamente modesta y necesita una mayor evaluación tanto en hombres como en mujeres
Determinantes socioeconómicos	El bajo nivel socioeconómico y el estrés laboral se asocian de forma independiente con el desarrollo y pronóstico de enfermedad vascular aterosclerótica en ambos sexos
Exposiciones ambientales	Medir la exposición acumulada a la contaminación del aire y del suelo, así como a niveles de ruido por encima del umbral, constituye un reto, pero puede mejorar la valoración individual del paciente. Se recomienda evitar la exposición prolongada a lugares de alta contaminación
Composición corporal	Las asociaciones entre el IMC, la circunferencia abdominal y el índice cintura-cadera con las EV se mantiene tras ajustar por los factores de riesgo convencionales, aunque no hay evidencia que mejoren la reclasificación. Sin embargo, son medidas simples y fáciles de recoger
Técnicas de imagen	Existen diferentes pruebas de imagen que se pueden usar para valorar el riesgo vascular, pero la coronariografía para evidenciar el calcio coronario es el que mejor puede reclasificar a pacientes, aunque tienen que ser interpretados cuidadosamente, y no es una prueba fácilmente factible de realizar en la práctica clínica

CV: cardiovascular; EV: enfermedades vasculares; IMC: índice de masa corporal; RR: riesgo relativo.

Modificada de^5^.

Hay otros aspectos importantes a considerar en la evaluación del riesgo: primero, el cálculo del riesgo en los pacientes que ya hayan padecido una EV, y poder priorizar la intensidad de nuestra intervención en este grupo de pacientes ante la presencia de nuevos tratamientos, como los inhibidores de la proproteína convertasa subtilisina/kexina tipo 9 (PCSK9), el inclisirán, o el ácido bempedoico, porque los predictores de riesgo pueden ser muy diferentes a los de prevención primaria. Las nuevas guías europeas recomiendan el cálculo a través del *SMART RISK score o SMART REACH model* o el *EUROASPIRE Risk model*.

Segundo, las guías europeas recomiendan el modelo de cálculo de RV de por vida (LIFE-CVD), útil sobre todo en pacientes menores de 45 años, permitiendo calcular el riesgo hasta los 90 años, y también ver los efectos del tratamiento en la esperanza de vida. El modelo DIAbetes Lifetime perspective model (DIAL) 2 para pacientes diabéticos también ha incorporado el concepto de riesgo de por vida^28^. La *American Heart Association* (AHA) en EE. UU. recientemente ha publicado una nueva escala de riesgo que tiene en cuenta el riesgo cardiaco-renal-metabólico, y que permite el cálculo del riesgo a los 30 años en aquellos individuos entre 30 y 59 años^29^. En España se ha desarrollado un modelo, a partir de población laboral española (IBERLIFERISK), que permite calcular el riesgo de por vida hasta los 75 años, en individuos de entre 18 y 65 años^30^.

Tercero, el reto de la comunicación del riesgo y la toma de decisiones compartidas en la práctica clínica. Además de la edad vascular y el riesgo relativo (RR), se han publicado nuevos abordajes para calcular el beneficio a largo plazo y los años de vida ganados con fármacos para el control de la dislipemia y la HTA, antiagregantes y abandono del consumo de tabaco, que de una manera muy ilustrativa se puede observar con las nuevas calculadoras tanto en prevención primaria como secundaria. Y cuarto, la utilización de los datos basales de los estudios de cohortes que se han incluido para desarrollar los diferentes modelos es muy simplista, ya que la realidad es que todos los posibles predictores no son estáticos y van cambiando a lo largo del tiempo de seguimiento. Una línea de investigación abierta y con mucho futuro es lo que se denomina *Machine Learning*^31^, que permiten analizar la relación entre predictores y eventos de forma más ajustada con modelos más complejos que los basados en una simple relación lineal entre el valor basal y el evento 10 años después.

En la tabla 5 se recoge la calidad de la evidencia y la fuerza de la recomendación sobre el cálculo del RV.

**Tabla 5:** Recomendaciones para el cálculo del riesgo vascular

Recomendación	Calidad de la evidencia	Fuerza de recomendación
Se recomienda el cálculo del riesgo vascular a todos los adultos de ≥ 40 años que no tengan una enfermedad vascular o que, por sus características, no sean de alto riesgo, mediante SCORE2 o SCORE2-OP (70 y 90 años).	Moderada	Fuerte a favor
Las tablas de riesgo constituyen una información complementaria y útil para ayudar a estratificar el riesgo y a tomar decisiones en el tratamiento de la dislipemia y de la HTA	Moderada	Fuerte a favor

HTA: hipertensión arterial.

Elaboración propia.

## Hipertensión arterial

Estas recomendaciones sobre HTA actualizan las de 2022^32^, siguiendo fundamentalmente las guías europeas de 2023^33^, y otras guías^5^, ^11^, ^34^, ^35^, ^36^, ^37^, ^38^, ^39^, ^40^ y novedades publicadas desde entonces.

La tabla 6 presenta de forma resumida, las recomendaciones, con su fuerza y la calidad de la evidencia que las sustenta.

**Tabla 6:** Recomendaciones en hipertensión arterial

*Recomendación*	Calidad de la evidencia	Fuerza de recomendación
*Definiciones*		
Clasificar la PA en consulta como: óptima, normal, normal-alta, HTA grado 1, 2 o 3	Baja	Fuerte a favor
Distinguir la HTA en 3 estadios:	Baja	Fuerte a favor
**•** Estadio 1 o HTA no complicada (sin LOD, ni diabetes, ni EV, ni ERC estadio ≥3)		
**•** Estadio 2 (presencia de LOD, diabetes o ERC estadio 3)		
**•** Estadio 3 (EV establecida o ERC estadios 4 o 5)		

*Valoración del riesgo vascular en HTA*		
Uso de SCORE2 o SCORE2-OP en HTA sin alto riesgo	Moderada	Fuerte a favor

*Cribado y diagnóstico*		
Estadio 1 o HTA no complicada (sin LOD, ni diabetes, ni EV, ni ERC estadio ≥3)Siempre que sea posible, medición de la PA en cualquier edad (incluso en < 18 años) como parte de cualquier visita médica	Baja	Débil a favor
La prueba inicial de cribado de HTA es la toma de la PA, estandarizada, en la consulta	Alta	Fuerte a favor
Periodicidad anual en: ≥ 40 años o si hay factores de riesgo para el desarrollo de HTA (sobrepeso-obesidad, cifras de PA normal-alta, raza negra)	Baja	Fuerte a favor
Periodicidad del cribado cada 3-5 años en todas las demás situaciones	Baja	Fuerte a favor
Cribado para trastornos hipertensivos en mujeres embarazadas con mediciones de la PA a lo largo de la gestación	Moderada	Fuerte a favor
Si disponible y asequible, confirmar diagnóstico de HTA mediante MAPA; además, uso ocasional en seguimiento, y es también útil para detectar fenotipos hipertensos (HTA enmascarada, HTA de bata blanca, HTA resistente verdadera, alteraciones del *dipping*, HTA nocturna)	Alta	Fuerte a favor
Si disponible, confirmar diagnóstico de HTA mediante AMPA; además, uso regular en seguimiento. Valores de HTA equivalentes a PA clínica de 140/90 mmHg son: MAPA diurno o AMPA: 135/85; MAPA nocturno: 120/70; MAPA 24 h: 130/80	Moderada	Fuerte a favor
Si no MAPA o AMPA, diagnóstico con PA en consulta (o toma automática en habitación adyacente tranquila), estandarizada (aparatos validados, reposo, postura y manguito adecuados) y repetida (2-3 mediciones en al menos 2-3 visitas secuenciales a lo largo de 4 semanas); salvo si HTA grado 3 (mantenida) en primera visita	Moderada	Fuerte a favor

*Estilos de vida*		
Las siguientes recomendaciones reducen la PA y el riesgo cardiovascular		
Pérdida mantenida del exceso de peso: 1 kg menos reduce la PAS/PAS en 1/1 mmHg, mediante dieta baja en calorías y ejercicio. Meta: IMC de 18,5-24,9 kg/m^2^.		
Reducción del consumo de sal en la dieta: < 5 g/día (∼ 2 g sodio/d), mediante p.ej. reducir el consumo de alimentos procesados y sal al cocinar		
Incremento de ingesta de potasio (4,7 g/día en la dieta; salvo en ERC avanzada): mediante, por ejemplo, frutas, verduras, nueces.		
Ejercicio físico aeróbico regular: mínimo de 150 min/semana de ejercicio moderado		
Dieta mediterránea regularmente: globalmente, más alimentos basados-en-plantas y menos basados-en-animales; uso de aceite de oliva en la dieta española		
Reducción del consumo de alcohol (si ya se bebe): ≤ 2 bebidas/día en varones y ≤ 1 bebida/día en mujeres	Fuerte	Fuerte a favor
No fumar para evitar aumento de PA ambulatoria e incrementar la reducción del riesgo vascular		
Reducir el estrés, por ejemplo, con ejercicios respiratorios, meditación o *mindfulness*	Débil	Fuerte a favor

*Tratamiento farmacológico*		
*Inicio tratamiento monoterapia*		
Iniciar tratamiento farmacológico en pacientes con HTA grado 2, o si alto riesgo vascular o EV demostrada	Alta	Fuerte a favor
Iniciar tratamiento farmacológico en pacientes con riesgo bajo moderado en grado 1 si tras 3-6 meses de CEV no se consigue alcanzar el objetivo terapéutico	Baja	Débil a favor
Iniciar tratamiento de forma inmediata si PA ≥ 180/110 tras confirmación con varias tomas	Alta	Fuerte a favor

*Tratamiento combinado*		
Iniciar tratamiento farmacológico combinado si la PA es 20/10 mmHg superior al objetivo terapéutico	Moderada	Fuerte a favor
Iniciar tratamiento farmacológico combinado si PA ≥ 140/90 en población general	Baja	Débil en contra

*Objetivos terapéuticos*		
Objetivos terapéuticos en población general 18-65 años < 140/90 mmHg	Alta	Fuerte a favor
Objetivos terapéuticos en población general 18-65 años, si se tolera < 130/80	Baja	Débil a favor
Objetivos terapéuticos en alto riesgo vascular, diabetes ERC, o EV: < 130/80	Moderada	Fuerte A favor
Objetivo terapéutico en > 65 años sin fragilidad: < 140/90	Alta	Fuerte A favor
Objetivo terapéutico en > 65 años con fragilidad: < 150/90	Muy Baja	Débil a favor

*Elección de fármacos*		
En población general no parecen existir diferencias entre los 3 principales grupos farmacológicos (IECA/ARA2, DT -incluidos los DT-like-, y CA-DHP) respecto a la disminución de morbimortalidad general	Moderada	Fuerte a favor
En el momento actual la evidencia de utilizar DT-like, frente a los otros DT no parece sostenible (por la débil evidencia de su superioridad y su escasa disponibilidad en asociación)	Baja	Débil en contra
En los pacientes con diabetes-2 y en los pacientes con ERC, los fármacos de elección deben ser los IECA/ARA2, sobre todo en caso de micro/macroalbuminuria	Alta	Fuerte a Favor
Los BB podrían continuar siendo fármacos de primera elección, sobre todo en jóvenes y en pacientes con incremento de su actividad simpática	Muy baja	Débil a favor
En insuficiencia cardiaca los hipotensores de elección son los IECA/ARA2 y los BB	Alta	Fuerte a Favor

AMPA: auto-medición de la PA; CE: calidad de la evidencia; CEV: cambios en estilos de vida; DT: diuréticos tiazídicos; ERC: enfermedad renal crónica; EV: enfermedad vascular; FR: fuerza de la recomendación; HTA: hipertensión arterial; IMC: índice de masa corporal; LOD: lesiones de órgano diana; MAPA: monitorización ambulatoria de la PA; PA: presión arterial.

Elaboración propia.

En el momento de finalizar este documento PAPPS-CV-2024, se acaba de publicar la guía de la Sociedad Europea de Cardiología para el manejo de la HTA^41^. Una revisión somera de la misma, nos indica que las directrices generales de prevención, diagnóstico y tratamiento son consistentes con lo que ya se había consensuado en el grupo, y a su vez, con matices, con las Guías Europeas de la Sociedad Europea de Hipertensión de 2023. No se modifica el criterio diagnóstico de HTA (PAS/PAS ≥ 140/90 mmHg). No obstante, es demasiado reciente su publicación para haberlas analizado y considerado a fondo en el presente documento. Si se detectara alguna seria desviación o aporte sustantivo en relación con las recomendaciones de este documento PAPPS-CV 2024, enviaríamos a la Revista (Atención Primaria) un *addendum* al respecto.

### Importancia epidemiológica y clínica

La HTA se define convencionalmente como cifras de PA sistólica/diastólica (PAS/PAD), obtenidas de forma protocolizada en la consulta (PA clínica), repetidamente elevadas: ≥ 140/90 mmHg^33^, ^37^, ^41^.

La HTA es un importante problema de salud pública por su alta prevalencia, especialmente en personas mayores, y por el escaso control de la PA clínica en la mayoría de las poblaciones y ámbitos clínicos^42^.

La PA elevada y la HTA como parte de ella, son importantes factores de riesgo para el desarrollo de EV, ya sea en forma de EV (enfermedad isquémica cardiaca, insuficiencia cardiaca, FA, enfermedad arterial periférica, aneurisma aórtico abdominal), cerebrovasculares (ictus o accidente isquémico transitorio [AIT]) o ERC, y de mortalidad total, incluyendo muerte súbita^33^, ^43^, ^44^. La PAS elevada es el principal contribuidor aislado de la carga global de enfermedad en el mundo, incluyendo España^45^, ^46^.

Las cifras disponibles de prevalencia y manejo de la HTA en el conjunto de España datan de hace más de cinco años^46^, necesitando una actualización que probablemente vendrá del seguimiento del estudio clínico Identificación de la poBlación Española de RIesgo CArdiovascular y reNal (IBERICAN)^43^, ^47^ o de la cohorte nacional Infraestructura de Medicina de Precisión asociada a la Ciencia y la Tecnología (IMPaCT)^48^. El estudio IBERICAN en adultos atendidos en consultas de atención primaria (AP) en España desde 2014-2018 reporta una prevalencia de HTA de 48% y control del 58% en los hipertensos, estando en monoterapia el 45% de los tratados^49^. Poblacionalmente, los datos provienen de 2010 (prevalencia, 33%; conocimiento, 59%, tratamiento, 47%; control, 23%^50^, ^51^, ^52^. Entre las causas del escaso control de la HTA en España figuran: el fenómeno de bata blanca, el escaso uso de monitorización ambulatoria de la PA (MAPA) y de automedición domiciliaria de la PA (AMPA) para confirmar el diagnóstico de HTA, insuficiente adherencia terapéutica (farmacológica y no farmacológica), uso insuficiente de terapia farmacológica combinada y escasa estimación del RV^46^.

### Definición de hipertensión y clasificación de los niveles de presión arterial

Debido a su variabilidad intrapersonal e interpersonal, la PA se debe medir repetidamente para acercarnos a su valor usual en la vida de la persona^33^, ^36^, ^37^. Aunque el riesgo de eventos comienza desde PAS/PAD de 115/75 mmHg, los valores de PA en consulta para diagnosticar HTA siguen siendo valores repetidos ≥ 140/90 mmHg, que muestran que a partir de esas cifras se duplica el riesgo de EV^33^, ^43^, ^45^, ^53^, ^54^, ^55^, ^56^.

Sin embargo, algunas influyentes guías estadounidenses^57^, apoyándose principalmente en el ensayo Systolic Blood Pressure Intervention Trial (SPRINT)^53^, criticado por la forma de medir la PA (medición automática en consulta [u-AOBP]), en un lugar tranquilo sin la presencia de un observador sanitario, que obtienen PA algo inferiores a las de otros estudios (que toman la PA en la consulta), rebajan el dintel diagnóstico de la HTA a PA ≥ 130/80 mmHg. En esta controversia apoyamos las recomendaciones europeas^33^, por razones principalmente logísticas y económicas, aunque también por la escasa evidencia que genera centrarse en un solo estudio. Así, una simulación sugiere que si en España se implementaran las guías estadounidenses (PA ≥ 130/80 mmHg)^57^, en vez de las europeas (≥ 140/90 mmHg), el número de nuevos hipertensos aumentaría unos cinco millones, con 1,4 millones más de nuevos candidatos a tratamiento farmacológico, situación difícilmente asumible para nuestro sistema nacional de salud^58^.

La PA puede clasificarse por sus niveles de PA, y la HTA en grados y estadios según presencia de comorbilidades^33^, ^37^; además, la aparición de la MAPA y AMPA como métodos diagnósticos o confirmatorios de HTA permite también hablar de HTA ambulatoria o en el hogar (tabla 7).

**Tabla 7:** Clasificación de la PA clínica en categorías, de la HTA en grados de PA y estadios de complicaciones, y de la HTA fuera de la consulta

Cifras de PAS/PAD (mmHg)	Categorías de PA	Estadios de HTA	Condiciones
*PA en consulta*			
< 120 y < 80	Presión arterial óptima	ESTADIO 1 (HTA no complicada)	Sin LOD ni complicaciones
120-129 y/o 80-84	Presión arterial normal	ESTADIO 2	LOD o ERC estadio 3 o DM
130-139 y/o 85-89	Presión arterial normal-alta	ESTADIO 3	EV establecida o ERC estadios 4 o 5
140-159 y/o 90-99	Grado 1		
160-179 y/o 100-109	Grado 2		
> 180 y/o ≥ 110	Grado 3		
PAS ≥ 160 y PAD < 90	HTA sistólica aislada		
PAD ≥ 90 Y PAS < 140	HTA diastólica aislada		
**PA fuera de consulta**	**Diagnóstico de HTA**		
*MAPA*			
Media diurna (actividad)	≥ 135 y/o ≥ 85		
Media nocturna (sueño)	≥ 120 y/o ≥ 70		
Media 24 horas	≥ 130 y/o ≥ 80		
*AMPA*			
Automedida domiciliaria	≥ 135 y/o ≥ 85		

Los sujetos con PAS y PAD en categorías diferentes se clasifican en la categoría más alta.

AMPA: automedición domiciliaria de la PA; EV: enfermedad vascular; ERC: enfermedad renal crónica; LOD: lesiones de órganos diana; MAPA: monitorización ambulatoria de la PA; PA: presión arterial.

Modificado de^33^, ^37^.

**Figura 4:**
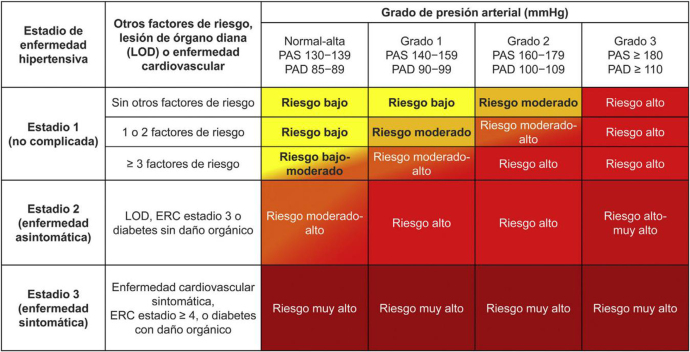
Riesgo vascular según categoría de presión y grado y estadio de hipertensión. Tomado de ^30^ y ^34^.

La HTA se asocia frecuentemente a otros factores de riesgo (p. ej., obesidad, dislipemia y DM2), que incrementan adicionalmente el RV global, como también lo hacen las lesiones de órganos diana (LOD), también llamado daño-orgánico-mediado-por-la-hipertensión; que son determinantes intermedios entre los factores de riesgo y la EV o la ERC avanzada^33^, ^36^, ^37^. Es importante determinar el riesgo global a 10 años en cualquier hipertenso (que no esté ya en alto riesgo) de cara al manejo de la HTA. Recomendamos las tablas de riesgo SCORE: SCORE2 (en edades: 40-69 años) y SCORE2-OP (70-89 años), que valora edad, sexo, PAS, tabaquismo y colesterol no-HDL (ver apartado «Tablas de riesgo vascular»), aunque esta herramienta de cálculo de RV no está exenta de cierto debate^59^. Además, hay tablas más detalladas que presentan el RV según los niveles de PA y estadios de HTA, que incorporan por tanto la PAD y las posibles LOD, EV y ERC^33^, ^36^, ^37^ (fig. 4).

### Cribado y pruebas diagnósticas

#### Cribado de hipertensión arterial

La PA tomada de forma estandarizada en la consulta sigue siendo el método de elección para el cribado (detección) de posible HTA^33^. Entre las pocas propuestas existentes sobre cribado, la *United States Preventive Services Task Force* (USPSTF)^60^, ^61^ recomienda el cribado oportunista de la HTA en adultos ≥ 18 años sin HTA conocida, mediante la medición de la PA en consulta. con una periodicidad anual en ≥ 40 años o en ≥ 18 años con riesgo alto de HTA (sobrepeso/obesidad, PA normal-alta, raza negra), y cada tres a cinco años para adultos de 18-39 años que no estén en riesgo de HTA y tengan PA previa normal^61^. La USPSTF también recomienda (año 2023) el cribado para trastornos hipertensivos en mujeres embarazadas con mediciones de la PA a lo largo de la gestación^62^. Aunque el USPSTF concluye que la evidencia actual es insuficiente para evaluar el balance beneficios/daños del cribado de HTA esencial en niños/adolescentes asintomáticos para prevenir EV posterior en la infancia o edad adulta^63^, las guías europeas^33^ recomiendan la medición de la PA en cualquier edad (incluso en < 18 años) como parte de cualquier visita médica, postura que, aunque con muy limitada evidencia, suscribimos siempre que sea posible en cada consulta en particular.

#### Diagnóstico de hipertensión arterial

Las mediciones fuera de la consulta mediante MAPA (generalmente durante 24 horas) o AMPA (dos a tres medidas en la mañana y en la noche durante tres a siete días, previo entrenamiento), tienen poder pronóstico predictivo de EV superior a las mediciones en consulta, y reducen el sobrediagnóstico relacionado con el fenómeno de bata blanca y el consiguiente probable sobretratamiento^33^, ^64^, ^65^, ^66^, ^67^, ^68^. Por ello, son útiles para la confirmación diagnóstica de HTA ante cifras elevadas de PA en consulta. Además, permiten el diagnóstico de HTA enmascarada (hipertensión en la vida diaria, y no en consulta), así como evaluar mejor la hipertensión resistente, o las presiones ocasional o persistentemente bajas en hipertensos tratados (no necesariamente inocuas), fenotipos que son detectables mediante estas técnicas de medida^33^, ^36^, ^37^, ^57^, ^65^, ^69^, ^70^, ^71^.

La MAPA permite además obtener cifras nocturnas de PA, fuertes predictores de riesgo; el estado *dipping* (grado de caída nocturna de la PA respecto al día); identificar la HTA resistente verdadera (pues excluye el efecto de bata blanca); examinar la variabilidad de la PA, y es la estrategia diagnóstica más costo-efectiva en AP^33^, ^36^, ^37^, ^57^, ^69^. Sin embargo, en AP la AMPA está más disponible^46^ y es un sustituto aceptable de la MAPA, en especial para monitorizar la terapia antihipertensiva, mejorar la adherencia terapéutica y optimizar el control^33^, ^36^, ^57^, ^72^; su uso como telemonitorización en situaciones en que el contacto personal es difícil (p. ej., pandemias o desastres naturales) es menos conocido^73^ pero prometedor. Las diferencias de accesibilidad para realizar MAPA o AMPA, podrían limitar sus indicaciones con inequidad en la actuación^74^.

Si los pacientes no dispusieran del dispositivo para la medición de la AMPA, podría valorarse la toma de PA en forma repetida con dispositivos de medición automática en la consulta (u-AOBP)^75^, en cuyo caso las cifras diagnósticas serían similares a las de la AMPA, aunque persistiría un pequeño efecto clínico; sin embargo, la u-AOBP solo es apoyada (con reducción de eventos) por el ensayo SPRINT^33^, ^53^, y no es considerada factible por muchos médicos de familia en España dado que no sería posible en AP disponer de una consulta aparte con un aparato automático programado para tomar la PA sin observador^76^. Otra modalidad es la *observer-measured home-BP*^46^, ^77^, en que no es el paciente sino otra persona entrenada quien le toma la PA en el hogar; no tiene efecto clínico, pero se añade un pequeño efecto observador.

Es de máxima importancia utilizar metodología estandarizada para medir la PA en la consulta o en domicilio. Es decir, seguir unas recomendaciones referentes a:

1. Información previa al paciente: con recomendaciones que deben facilitarse antes de la toma, ya sea en consulta o en domicilio: reposo previo, no consumo de tabaco previo, no ingesta de alimentos, café o alcohol en los 30 minutos previos a la medida y vejiga urinaria vacía antes de la medición.
2. A la técnica:
  a) Utilizar tensiómetros automáticos oscilométricos con manguito braquial, validados (si automáticos no disponibles, usar aneroides calibrados u otros métodos solventes -listas *online* de aparatos: http://www.stridebp.org^78^ ó dableducational.org^79^)^33^, ^36^, ^80^.
  b) Toma estandarizada: posición adecuada, espalda apoyada, brazo a la altura del corazón, no cruzar las piernas.
  c) Utilizar un manguito apropiado al perímetro braquial, y emplear el brazo dominante (si no se conoce el brazo con mayores cifras en primera visita).
  d) Repeticiones: nunca realizar un diagnóstico con una sola toma de PA en consulta; realizar dos a tres mediciones consecutivas con un intervalo de 1-2 minutos entre ellas (si tres tomas, promediar las dos últimas) en al menos dos consultas separadas una a cuatro semanas, a menos que la PA en primera visita registre HTA grado-3 o haya alto RV. Y, de cara al diagnóstico de HTA con AMPA: dos medidas (antes de desayuno y cena), durante un mínimo de tres, y preferiblemente siete días antes de la visita en consulta.
  e) En sospecha de hipotensión ortostática, tomar la PA 1 y 3 minutos tras estar de pie^33^, ^36^, ^37^.

Nuestra recomendación final es la confirmación de las cifras elevadas obtenidas en consulta, mediante MAPA o AMPA cuando estén disponibles, para el diagnóstico de la HTA e identificación de fenotipos hipertensivos.

### Tratamiento

#### Medidas no farmacológicas y estilos de vida

Los principales factores de riesgo modificables de HTA son: exceso de peso (IMC ≥ 25 kg/m^2^), ingesta alta de sal (> 4,6 g/día), ingesta elevada de alcohol (≥ 1 bebidas/d en mujeres y ≥ 2 en hombres), y baja actividad física (< 3 h/semana de actividad física moderada o vigorosa)^33^, ^37^, ^81^. Entre los no modificables: edad, sexo, historia familiar de enfermedad cardiovascular (ECV) o HTA precoces y susceptibilidad genética^82^. Por último, la exposición al ruido ambiental (p. ej., tráfico) y la contaminación atmosférica son estresores modificables en cierta medida, que aumentan la PA^5^, ^33^.

Las medidas de estilos de vida saludables reducen eficazmente la PA, complementan el tratamiento farmacológico de cualquier hipertenso, y reducen el número de medicamentos antihipertensivos, pues reducen la PA en ∼ 4-5 mmHg y el RV^5^, ^33^, ^83^, ^84^, ^85^, ^86^, ^87^, ^88^, ^89^.

- Pérdida de peso, mediante combinación de dieta baja en calorías y ejercicio (por cada kg de peso perdido, la PAS/PAD se reduce en 1/1 mmHg; también permite reducir la intensidad de fármacos antihipertensivos y los lípidos plasmáticos). Objetivo recomendado: IMC = 18,5-24,9 kg/m^2^.
- Reducción de la ingesta de sodio (reducir 75 mmol/d el sodio urinario -4,4 g de sal- reduce la PA en 4,2/2,1 mmHg, y también reduce la necesidad de fármacos antihipertensivos). Ingesta recomendada: < 6 g/d de cloruro sódico (ClNa) (sal); mediante opciones dietéticas (menos alimentos procesados) y reformulación de alimentos con menos contenido en sal. Un equivalente práctico es: 2,0 g de Na ∼ 87mmol y ∼ 5 g de ClNa. El consumo de sustitutivos de la sal (cloruro potásico, salicornia) aún no está suficientemente avalado por evidencias.
- Aumento de la ingesta de potasio (un suplemento de 75 mmol/d reduce la PA 3,1/2 mmHg y el riesgo de ictus). Ingesta recomendada: 4,7 g/día en la dieta (frutas, verduras, nueces).
- Moderación de la ingesta de alcohol (reducción a la mitad en bebedores de ≥ 6 bebidas/día, reduce la PA 5,5/4 mmHg). Recomendación: ≤ 2 bebidas/día en varones y ≤ 1 bebidas/d en mujeres (1 bebida ∼ 10 g).
- Actividad física aeróbica (≥ 3 sesiones de 30-60 min/semana reduce la PA 5,1/2,2 mmHg). Recomendación: 30 min/día, la mayoría de los días de la semana (≥ 150 min/semana).
- *Dietary Approaches to Stop Hypertension* (DASH): rica en frutas, verduras y lácteos bajos en grasa, y baja en grasa saturada y total, reduce la PA 5,5/3 mmHg vs. dieta control, en las dos primeras semanas del estudio; y redujo la PA 11,4/5,5 mmHg en hipertensos); recomendación: tomarla regularmente. En España, el patrón dietético más adecuado para prevenir y tratar la HTA sería la dieta mediterránea, similar a la DASH pero que incluye el uso de aceite de oliva^90^, ^91^.

Por último, una reciente revisión muestra que algunas terapias alternativas biológicas (consumo moderado de café) y algunas técnicas no biológicas (*mindfulness*, meditación, tai-chi), presentan relación riesgo/beneficio razonable en relación con la PA y la HTA; sin embargo, no obvian la necesidad de estilos de vida saludables y fármacos cuando estén indicados^92^.

Se recomienda firmemente el cese de este hábito al paciente hipertenso fumador para reducir su RV^5^, ^33^. Los programas para dejar de fumar reducen significativamente tanto la PAS como la diastólica. Los resultados fueron más significativos en el grupo de pacientes hipertensos que en el de no hipertensos^93^.

##### Adherencia al tratamiento antihipertensivo, papel de las profesiones implicadas y nuevas tecnologías

El punto más débil del tratamiento no farmacológico es la baja persistencia de las medidas prescritas, y un reto tecnológico es vigilar más efectivamente la adherencia terapéutica^33^, ^46^, ^69^, ^94^. Es muy importante detectar la falta de adherencia al tratamiento farmacológico antihipertensivo, causa frecuente del bajo nivel de control de la HTA^5^, ^33^.

El personal de enfermería tiene un prominente papel en el manejo de la HTA, en consultas de AP y hospitalaria^95^, y las farmacias comunitarias van asumiendo crecientemente responsabilidad y compromiso^96^.

Además, se han desarrollado nuevas tecnologías efectivas para mejorar la adherencia terapéutica y el control de la HTA, incluyendo automedición de la PA, guías de implementación, polipíldoras, *smart-phones* y *telehealth*^97^, ^98^, ^99^, ^100^. Por último, en la gestión (e investigación) de la HTA va emergiendo una combinación de inteligencia artificial (IA) y tecnologías portátiles como primera oportunidad real para una medicina cardiovascular de precisión. La futura atención de la HTA mejorada con IA podría fomentar la concienciación del paciente, el autocontrol y la adherencia al tratamiento, aunque no se han de olvidar las importantes limitaciones que aún presenta este enfoque^101^, ^102^. Ante la sospecha de HTA refractaria al tratamiento, y antes de plantear procedimientos diagnósticos costosos o terapias invasivas como la denervación renal; se ha propuesto incluir la observación directa (presencial), de la toma de medicación en consulta, seguida de una MAPA (que puede limitarse a unas horas) y complementarse con medidas de PA en consulta (p. ej., cada 1,5-2 horas)^103^, que permita descartar o confirmar si la falta de adherencia fuera la causa.

#### Tratamiento farmacológico

Los ensayos clínicos son el patrón de oro de la evidencia de calidad, pero es necesario tener en consideración estudios bien diseñados de cohortes y ecológicos, que se acercan más a la realidad cotidiana de las consultas de AP, y continuar apostando por recomendaciones Glycemia Reduction Approaches in Diabetes: a comparative Effectivenes (GRADE) que sí tienen en cuenta estos aspectos.

#### Inicio del tratamiento farmacológico

Todas las guías de práctica clínica consultadas^5^, ^11^, ^33^, ^34^, ^35^, ^36^, ^37^, ^38^, ^39^, ^40^, excepto la guía de la AHA^57^, indican iniciar tratamiento farmacológico en población general a partir de PA ≥ 140/90 mmHg si el RV del paciente es elevado.

La tabla 8 resume las recomendaciones sobre cuándo iniciar tratamiento farmacológico, los objetivos terapéuticos a alcanzar y las especificaciones respecto a determinados grupos de pacientes (edad, RV, co-morbilidades)^104^, ^105^, ^106^.

**Tabla 8:** Recomendaciones de cifras de presión arterial para iniciar tratamiento farmacológico y objetivos terapéuticos, según diversas guías clínicas de hipertensión

Guías de práctica clínica	Presión arterial para iniciar tratamiento y grupos de pacientes	Objetivos terapéuticos por grupos de edad (mmHg)
AHA 2017	Población general: 140/90 EV o alto RV: 130/80	< 130/80
EUROPEA-SEH-SEC 2018	Población general: 160/100 Alto RV o tras fracaso de estilos de vida: 140-159/90-99	Población general: 140/90 < 130 si se tolera 18-65 años: 120-129 si se tolera > 65 años: 130-139 si se tolera
EUROPEA HTA 2023	18-79 años: ≥ 140/90 ≥ 80 años: ≥ 160. Puede 140-160 en > 80 años en pacientes sin fragilidad. Si ECV: 130-139/80-89	18 a 64 años: 130/80 65-79 años: < 140/80; < 130/80 si si se tolera ≥ 80 años: 140-150/< 80, 130-139/< 80 si se tolera bien. No < 120/70
NICE 2019-2022	Bajo riesgo CV: 160/100 Si LOD, diabetes, ERC o RV > 10% (QRISK): 140/90 > 80 años: > 150/90	<65 años: 140/90 > 65 años: 150/90 ERC + macroalbuminuria: 130/80
CANADÁ 2020- 2022	Bajo riesgo: 160/100 LOD o FRV: 140/90 Diabetes: 130/80 Alto RV: 130	Bajo Riesgo: 140/90 LOD o FRV: 140/90 Diabetes 2: 130/80 Alto RV y EVA: 120
ISH 2020	Población general: 160/100 Alto RV, DM, ERC, EVA o si fallan CEV (3-6 meses): 140/90	Población general: 140/90 (bajar al menos 20/10 mmHg) < 65 años: Optima 130/80 > 65 años: 140/90
OMS 2021	Población general: 140/90 EVA: > 130 (Fuerte) DM, ERC, Alto RV: > 130 (condicional)	Población general: 140/90 EVA: < 130 (fuerte) EDM, ERC, alto RV: < 130 (condicional)
KDIGO 2O21	No definido. Presumible: > 120/80	Población general con ERC: < 120/80

CEV: cambios de estilo de vida; ERC: enfermedad renal crónica; EV: enfermedad vascular; EVA: enfermedad vascular aterosclerótica; FRV: factores de riesgo vascular; LOD: lesiones de órgano diana; RV: riesgo vascular.

Adaptado por los autores de las referencias 104, 106, 113. Y de las guías referenciadas en el texto.

Las guías europeas^11^, ^33^, ^37^, aunque recomiendan iniciar terapia antihipertensiva junto con las medidas de estilo de vida en todos los hipertensos (PA clínica ≥ 140/90 mmHg) de 18 a 79 años, especifican que si el paciente presenta cifras en consulta < 150/95 mmHg, ausencia de daño orgánico y bajo RV, se podría retrasar el tratamiento farmacológico hasta después de tres a seis meses de medidas de estilo de vida. Sin embargo, la guía AHA^57^ propone tratamiento farmacológico si la PA es ≥ 130/80 mmHg (HTA «estadio 1» en dicha guía), y comenzar con dos fármacos a partir de HTA «estadio 2» (140/90 mmHg). Esto se basa fundamentalmente en el estudio SPRINT^53^, ^107^ y varios metaanálisis^54^, ^108^. En SPRINT los pacientes tenían alto RV y se excluyeron los pacientes con diabetes y con ictus; el metaanálisis^54^ del grupo *Blood Pressure Lowering Treatment. Trialist Collaboration* (BPLTTC) estimó que una reducción de 5 mmHg de la PAS reduciría el riesgo de eventos un 10%, incluso con PAS < 120, lo que apoyaría la idea de tratar independientemente del RV; sin embargo, varias recientes revisiones^109^, ^110^ han rebatido dicha conclusión. El estudio HOPE-3^111^, en pacientes con PA media de 138,1 mmHg y con RV bajo-moderado no pudo demostrar beneficio a nivel de eventos vasculares, ni en un seguimiento a los tres años posteriores al mismo^112^.

En resumen, al igual que en revisiones anteriores del PAPPS^32^, seguimos recomendando iniciar tratamiento farmacológico inmediato de la HTA grado 2 (≥ 160/100 mmHg), y en grado 1 en caso de RV elevado o presencia de ECV; en caso de HTA grado 1 en RV moderado/bajo se debería iniciar tratamiento farmacológico si no se alcanzan cifras controladas tras unos meses con medidas de estilos de vida. La evidencia hasta la fecha sugiere que los umbrales terapéuticos basados en la PA no son tan eficientes como los umbrales basados en el RV, y, por tanto, seguimos apostando por considerar el tratamiento farmacológico en función del RV del paciente, y no exclusivamente en función de las cifras de PA. El lugar donde se establezca ese umbral de riesgo dependerá de varios factores y puede variar en una región a otra dependiendo de la disponibilidad de recursos y prioridades, aunque tampoco deberíamos descartar la posibilidad de reducir la PA simplemente porque el nivel de PA del paciente sea normal cuando el paciente podría beneficiarse de una importante reducción del riesgo de EV en el futuro^113^.

##### Elección del tratamiento farmacológico

Actualmente existe el convencimiento de que los beneficios del tratamiento antihipertensivo se originan en la reducción de la PA *per se*, sin constatar diferencias importantes en cuanto a los diferentes grupos farmacológicos. Por ello, la mayoría de las guías^5^, ^11^, ^32^, ^33^, ^34^, ^35^, ^36^, ^37^ establecen que los diuréticos tiacídicos (DT) (especialmente los llamados DT-*like*: clortalidona e indapamida), los inhibidores de la enzima convertidora de la angiotensina (IECA), los antagonistas de los receptores AT1 de la angiotensina II (ARA 2) y los antagonistas del calcio dihidropiridínicos (ACA-DHP) serían los fármacos de elección. Las guías europeas^11^, ^33^, ^37^ también incluyen a los betabloqueadores (BB) como fármacos de primera elección, lo que ha sido argumentado en una reciente revisión^113^. Una excepción a esta regla proviene de la guía National Institute for Health and Care Excellence (NICE)^40^, que sigue apostando en < 55 años de raza blanca por un IECA (ARA 2 si intolerancia o raza negra); o si tienen > 55 años o son de origen familiar africano-negro o africano-caribeño (sin diabetes) optan por ACA-DHP, siendo los DT una alternativa si no se toleran los anteriores. La evidencia parece haber demostrado que la mejor opción terapéutica al utilizar fármacos antihipertensivos es combinar aquellos con diferentes mecanismos de acción^114^. En cualquier caso, podría extrapolarse de dichos resultados un efecto positivo específico de protección renal y de hipertrofia ventricular izquierda (HVI) para los IECA/ARA2, más allá del efecto hipotensor intrínseco de cada uno de ellos.

Respecto a una supuesta superioridad de los DT frente a otros grupos farmacológicos varias publicaciones, entre las que destaca una reciente revisión^115^, un seguimiento del estudio Antihypertensive and Lipid-Lowering Treatment to Prevent Heart Attack Trial (ALLHAT)^116^; así como una publicación Cochrane^117^, no han podido objetivar un descenso de la mortalidad, aunque sí de otros eventos cardiovasculares (como ictus en DT frente a IECA).

Por último, aunque se ha especulado que posiblemente los DT-*like* (clortalidona e indapamida), con mayor vida media y un número mayor de estudios^118^, puedan ser superiores en eficacia a los clásicos (hidroclorotiazida), aspectos pragmáticos, como la falta de disponibilidad en asociación, limitan su uso^119^. Por tanto, hasta que se demuestre lo contrario, la importancia de estas diferencias sigue siendo especulativa, y los médicos probablemente deberíamos asumir que los eventos cardiovasculares se reducirán de manera similar entre los agentes cuando se produzca una reducción equivalente de la PA^119^.

##### Combinación frente a monoterapia de inicio

Algunas revisiones^120^, ^121^, ^122^ y varias guías recientes de práctica clínica^11^, ^33^, ^34^ enfatizan la elección de un tratamiento antihipertensivo en combinación a dosis bajas, preferentemente en una sola píldora, frente a monoterapia, incluso en estadios iniciales de HTA, aunque dichas combinaciones generalmente son recomendadas si la PA está 20/10 mmHg por encima de su objetivo terapéutico, o en grado 2 de HTA^121^. De hecho, la terapia combinada de inicio tiene una evidencia muy limitada. Una revisión Cochrane^123^, analizó la existencia de ensayos clínicos con al menos 12 meses de duración y con 50 o más pacientes en cada brazo, que comparasen la monoterapia inicial con terapia combinada de inicio, y no pudo encontrar ningún estudio que cumpliera con estos criterios; además, aunque sí objetivó menores cifras de PA en el tratamiento combinado, no se demostró que el tratamiento con terapia combinada fuera superior a la monoterapia en reducción de mortalidad total, mortalidad cardiovascular o eventos cardiovasculares. Ello a pesar de que el tratamiento inicial combinado suele ser bien tolerado por los pacientes, y puede mejorar la adherencia terapéutica y el control de la PA^124^, lo cual es refrendado por las guías europeas^11^, ^33^, ^37^. Además, las pocas evidencias existentes proceden de pacientes con alto riesgo cardiovascular (ensayo SPRINT)^107^, ^108^ o con diabetes (ensayo Action to Control Cardiovascular Risk in diabetes [ACCORD])^125^, no generalizables a sujetos de menor riesgo.

El tratamiento combinado de entrada podría suponer una excesiva medicalización de los pacientes hipertensos. Por ello, consideramos necesaria más evidencia antes de una pronunciación categórica para recomendar la combinación de fármacos como tratamiento inicial de la HTA. Los hipertensos en los que estaría indicada la combinación de inicio, por tanto, serían aquellos con hipertensión complicada y aquellos cuya PAS y PAD está más de 20/10 mmHg, respectivamente, por encima de su PA objetivo. Esta fue la línea que mantuvimos en la anterior versión de estas recomendaciones, y la que seguimos manteniendo en la actualidad.

Actualmente existen numerosos estudios comparando la monoterapia con doble terapia, triple y cuádruple terapia en combinación a bajas dosis^126^, ^127^, ^128^, ^129^, ^130^, estos adolecen de gran heterogeneidad y escasa duración, sin posibilidad de objetivar disminución en desenlaces de morbimortalidad, aunque sí parece consensuarse entre expertos que ocurre un mayor descenso de la PA, y disminución de la inercia terapéutica, con su uso.

### Estrategias terapéuticas

#### Problemas con la tasa de control y la implementación de las guías

Algunas editoriales recientes^98^, reafirman la idea de que está empeorando el control de la HTA en muchos países, incluido EE.UU. y, por ende, la implementación de las guías de práctica clínica. Es un debate universal valorar cuáles podrían ser las causas, pero parece estar demostrándose que el uso de protocolos que incluyan promoción de la salud, mediciones correctas, equipos de atención médica bien formados, junto a protocolos simples y prácticos que incluyan combinaciones de medicamentos en una sola pastilla para mejorar la cumplimentación, están siendo efectivos^131^, ^132^.

#### Objetivos terapéuticos

En población general, existe amplio consenso en recomendar cifras de PA < 140/90 mmHg como objetivo terapéutico en todos los hipertensos, y que debería dirigirse hacia cifras de 130/80 mmHg o incluso inferiores (120-129 mmHg), sobre todo en pacientes de alto RV y en diabetes^5^, ^11^, ^33^, ^34^, ^35^, ^36^, ^37^, ^38^, ^39^, ^40^, si se toleran. Sin embargo, no todas las revisiones han encontrado estos efectos positivos sobre la mortalidad total o cardiovascular para objetivos terapéuticos más rígidos en EV^133^. El objetivo terapéutico de la PAD es alcanzar un rango de 70-79 mmHg en todos los pacientes. El límite de seguridad de la PA, por debajo del cual el riesgo supera al beneficio, está en torno a los 120/70 mmHg, incluso independientemente de la edad^134^. Estas consideraciones afectan a todos los hipertensos, incluyendo aquellos con o sin diabetes^110^, EV previa^133^ o ictus^135^.

Es importante individualizar el objetivo de PAS basado en una variedad de factores, incluida la presencia de condiciones comórbidas subyacentes, RV, posibles efectos adversos efectos asociados con los medicamentos antihipertensivos, así como consideraciones inherentes al precio de los medicamentos y de las preferencias del paciente^104^.

#### Cronoterapia

Actualmente no existe evidencia sólida y consensuada que justifique un mayor beneficio en objetivos clínicos relevantes (mortalidad total o de origen cardiovascular, episodios cardiovasculares mayores [MACE]) al utilizar dosis nocturnas de hipotensores frente a dosis diurnas^136^. Por tanto, a menos que pretendamos reducir específicamente la PA nocturna, los medicamentos antihipertensivos deberían tomarse en el momento del día que sea más conveniente, optimizando la adherencia y minimizando los efectos indeseables^137^. Una reciente editorial^98^ concluía lo siguiente: existen datos limitados y se han documentado defectos de los diseños en ensayos de cronoterapia, por lo que no existe evidencia adecuada para determinar qué régimen de terapia farmacológica con dosificación horaria (mañana versus noche) tiene más efectos beneficiosos sobre los resultados cardiovasculares o los eventos adversos. La dosificación nocturna de fármacos antihipertensivos no es más ni menos efectiva que la administración matutina para reducir la PA durante 24 horas.

### Situaciones especiales en hipertensión arterial

#### Deterioro cognitivo e ictus

Se ha relacionado la elevación de las cifras de PA con el desarrollo de deterioro cognitivo y déficit mnésicos a medio y largo plazo^138^. Tanto la HTA como la diabetes y el tabaquismo se han asociado con un deterioro cognitivo acelerado en edades medias de la vida^139^. Se ha objetivado que, en comparación con la normotensión, los individuos con hipertensión tratada y controlada, no tratada, y tratada pero no controlada tienen un 83%, 97% y 162% más de riesgo de desarrollar un accidente cerebrovascular^140^. Asimismo, un reciente estudio en vida real^141^ ha concluido que objetivos de control más estrictos disminuyen la reincidencia de ictus, y, varios meta-análisis y estudios previos (ACCORD)^125^ objetivaron que el mayor beneficio obtenido en la disminución más estricta de PAS fue para el ictus.

#### Diabetes

Respecto a los objetivos terapéuticos más estrictos, frente a más laxos, en DM2, no todas las revisiones han encontrado asociaciones significativamente beneficiosas para el control más estricto, habiéndose documentado un menor número de ictus y de macroalbuminuria pero no de mortalidad o de beneficios a nivel cardiovascular^142^. No hay que olvidar que, en este, y en la mayoría de estudios con objetivos de control de PA más estrictos, aumentaban considerablemente los efectos secundarios. Ello no es óbice para según argumentan varias revisiones^143^, ^144^, considerar un objetivo terapéutico < 130/80 mmHg para los pacientes diabéticos. Incluso, una reciente revisión sistemática^145^ viene a replantearse si no se debería ser más rígido aún en el objetivo de control de PAS en diabetes; en ella niveles de PAS de 120-124 mmHg disminuyeron el riesgo de EV grave respecto a las cifras superiores a 135 mmHg fundamentalmente a expensas de la reducción de ictus, aunque no fueron significativos los descensos de mortalidad, total, mortalidad de origen cardiovascular, el IAM o la insuficiencia cardiaca.

#### Enfermedad renal crónica

Las últimas guías Kidney Disease: Improving Global Outcomes (KDIGO) recientemente publicadas^146^ indican la necesidad de alcanzar objetivos terapéuticos de 120 mmHg de PAS, cuando sean tolerados, individualizando este objetivo en función de fragilidad, expectativas de vida y presencia de ortostatismo. En caso de pacientes con alta fragilidad, riesgo de caídas y fracturas, limitada expectativa de vida o hipotensión ortostática sintomática, considerar objetivos de control menos intensivos (alrededor de 130/80 mmHg según tolerancia). Existen algunas críticas recientes que no aceptan este objetivo tan estricto^147^, ^148^.

Respecto a la elección de los hipotensores, el patrón oro siguen siendo los IECA/ARA2 en los pacientes con ERC, con o sin diabetes, con mayor evidencia (1 B) en pacientes con diabetes con moderada o grave albuminuria (A2-A3) o sin diabetes con albuminuria de alto grado (A3); y algo menos de evidencia (2 C) en ERC sin diabetes y moderado incremento de albuminuria (A2).

#### Hipertensión sistólica aislada y personas mayores

La dificultad básica comienza a la hora de estipular cuál es la edad que define el concepto de anciano (o persona mayor). Desde 60-65, 70 e incluso 80 años, son valores que se establecen cuando se trata de la HTA en el «paciente mayor o anciano»^149^. En líneas generales, las guías de práctica clínica establecen un objetivo terapéutico igual que en población general (< 140/90 mmHg), intentando llegar a 130 mmHg si se tolera por haber demostrado igualmente la disminución de los MACE^150^. Uno de los puntales más importantes para apoyar esta tesitura de control < 130 mmHg parte del estudio SPRINT^151^ y el estudio chino Strategy of Blood Pressure Intervention in the Elderly Hypertensive Patients (STEP)^152^. Probablemente la excepción a estas recomendaciones, estén relacionadas con la existencia de pluripatología y, sobre todo, fragilidad, más que la edad en sí de los pacientes.

Respecto a los hipotensores, exceptuando la guía NICE^40^, que sigue preconizando el uso de ACA-DHP en pacientes mayores de 55 años o de raza negra, y diuréticos, el resto de las guías actuales no indican una predilección por un grupo farmacológico, excepto para limitar el uso de BB en este grupo de edad (excepto la guía europea)^33^.

En la HTA sistólica aislada (HSA) existe la misma posibilidad de conseguir efectos positivos con el descenso de la PA que con el resto de grupos. Una reciente revisión concluía que los pacientes con HSA en grados 1 y 2 deben ser tratados, siendo el tratamiento eficaz y seguro hasta niveles objetivo de PAS inferiores a 140 mmHg, y posiblemente incluso inferiores a 130 mmHg si se toleran^153^.

#### Enfermedad arterial coronaria

Aunque clásicamente se pueda incluir como prevención secundaria a los pacientes con enfermedad arterial coronaria (EAC) y, por tanto, en las guías consultadas se acepta un objetivo terapéutico de 130/80 mmHg, no existen muchos estudios al respecto para evidenciar este objetivo. De hecho, una reciente revisión del grupo SPRINT^154^ no pudo encontrar un resultado positivo en los pacientes coronarios frente a los no coronarios. Respecto a la clase de tratamiento farmacológico, estarían indicados los IECA (y ARA 2 si intolerancia), BB (sobre todo en fracción de eyección [FE] deprimida), y ACA-DHP (sobre todo en angor pectoris)^33^. En caso de una frecuencia cardiaca elevada (> 80 lpm) podrían considerarse, además de los BB, los Calcioantagonistas (CA) no DHP^33^.

#### Insuficiencia cardiaca

**Figura 5:**
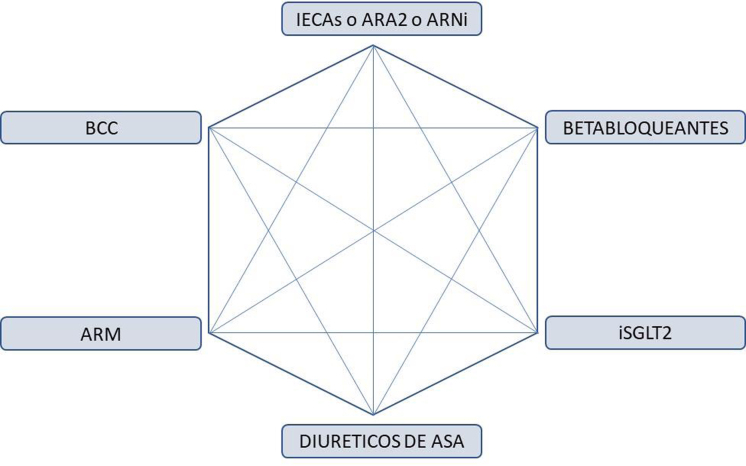
Hexágono terapéutico de fármacos indicados en el paciente hipertenso con insuficiencia cardiaca. IECAs: inhibidores de la enzima conversora de angiotensina; ARA2: antagonista de receptores de angiotensina; ARNi: inhibidores de receptores de angiotensina/neprilisina; BCC: bloqueantes de canales del calcio; ARM: antagonistas de receptores de mineralcorticoides; ISGLT2: inhibidores del cotransportador de sodio-glucosa tipo 2. Adaptado de ^149^.

Más del 90% de los pacientes que desarrollan insuficiencia cardiaca tienen HTA^155^. En la insuficiencia cardiaca las guías^156^, al igual que en la cardiopatía coronaria, recomiendan un objetivo terapéutico de 130/80 mmHg. Las clases farmacológicas indicadas para el tratamiento específico de la HTA serían los IECA/ARA2, BB y diuréticos tiazídicos-*like* (sobre todo en prevención de insuficiencia cardiaca) más diuréticos de asa (para disminuir la disnea)^155^. A estos grupos hay que añadir los tratamientos que de forma sistemática deberían ir incluidos en todo paciente con insuficiencia cardiaca, como serían los inhibidores del cotransportador de sodio-glucosa tipo 2 (iSGLT2), o los antagonistas de aldosterona e inhibidores del receptor de angiotensina-neprilisina (ARNi) en caso de insuficiencia cardiaca con FE deprimida. Una forma gráfica de definir el tratamiento de HTA en la insuficiencia cardiaca es el hexágono clásico del tratamiento combinado en HTA, reeditado para esta ocasión^157^ (fig. 5).

## Dislipemia

### Importancia epidemiológica

En el año 2020, el valor medio de colesterol no-HDL en España fue de 3,2 mmol/L (123,55 mg/dL) para las mujeres y 3,3 mmol/L(127.41 mg/dL) para los hombres. Estos valores están por debajo de la media europea, que es de 3,4 mmol/L (131,27 mg/dL) para las mujeres y 3,5 mmol/L (135,14 mg/dL) para los hombres, y han ido en descenso desde los años 80^158^.

Por otro lado, el valor medio de c-HDL en el 2020 en España fue de 1,6 mmol/L (61,78 mg/dL) para las mujeres y 1,2 mmol/L (46,33 mg/dL) para los hombres. Frente a los valores europeos, las mujeres españolas presentaron un valor superior al promedio europeo de 1,5 mmol/L (57,92 mg/dL), mientras que los hombres tuvieron un valor inferior a la media europea de 1,3 mmol/L (50,19 mg/dL). Estos valores de c-HDL se han mantenido estables en España en los cinco años previos al 2021^158^.

### Cribado y pruebas diagnósticas

Las pruebas recomendadas para el cribado de la dislipemia es la determinación del colesterol total, c-LDL, c-HDL y triglicéridos. Aunque las cifras de colesterol total, c-LDL y c-HDL se interpretan en función de la estratificación del riesgo cardiovascular, se suele considerar como hipercolesterolemia una cifra de colesterol total > 200 mg/dL o cifras de c-LDL > 130 mg/dL, mientras que el efecto protector del c-HDL se considera a partir de los 50 mg/dL y actuaría como factor de riesgo por debajo de los 45 mg/dL. La hipertrigliceridemia se define como triglicéridos > 150 mg/dL.

No hay evidencias suficientes para establecer un rango de edad y una periodicidad determinados para determinar el colesterol sérico en población sana, por lo que la actitud más razonable es incluirlo en cualquier análisis de sangre solicitado al paciente, con una periodicidad mínima de cuatro años y a partir de los 18 años. Sobre los parámetros a incluir en un perfil lipídico básico, y ampliado; así como las recomendaciones para su determinación e informes en los laboratorios clínicos españoles se puede consultar un consenso reciente de Arrobas et al.^159^.

No se recomienda determinar de manera rutinaria fracciones lipídicas como la apolipoproteína B o la liproproteína (a) (Lp[a]), aunque existen algunos estudios observacionales que han mostrado una asociación entre niveles altos de Lp(a) y aumento de la morbimortalidad cardiovascular. La Lp(a) viene determinada genéticamente, por lo que una única determinación en la vida de la persona sería suficiente y podría aportar información adicional sobre el riesgo residual de los pacientes. Actualmente, hay ensayos clínicos fase III en marcha que evaluarán la eficacia de nuevos fármacos que reducen los niveles de Lp(a).

### Tratamiento farmacológico

#### Estatinas

Las estatinas son, entre los diferentes fármacos hipolipemiantes, los que más evidencias disponen en la reducción de la enfermedad cardiovascular y con una excelente relación riesgo/beneficio en población de riesgo^160^, ^161^. Existe una relación lineal entre los miligramos de c-LDL reducidos con estatinas y la reducción de la enfermedad cardiovascular, y se ha cuantificado que descensos de 1 mmol/L (39 mg/dL) de c-LDL determinan una reducción de los MACE (IAM e ictus mortal o no mortal y recibir un tratamiento de revascularización) de un 21%; y se sugiere que reducciones de 2-3 mmol/L reducirían el riesgo un 40-50%^162^.

Además, de los MACE, las estatinas también reducen la mortalidad total, la mortalidad cardiovascular, la mortalidad coronaria y los ictus isquémicos^163^, pero sin resultados en la prevención de los ictus hemorrágicos^164^. Se ha demostrado que los beneficios cardiovasculares ocurren en diferentes grupos de población. Entre estos se encuentran los pacientes con o sin enfermedad cardiovascular, con ictus, personas con diabetes, en varones y mujeres, en mayores de 65 años e, incluso, en pacientes con riesgo cardiovascular bajo^163^, ^165^, ^166^. La extensa población en la que las estatinas han demostrado su eficacia hace que, prioritariamente, se recomiende el tratamiento en los grupos con mayor riesgo cardiovascular: pacientes con enfermedad cardiovascular o pacientes de riesgo muy alto, pacientes con diabetes, pacientes con riesgo alto mediante tablas de riesgo o pacientes con un c-LDL muy elevado.

#### Inhibidores de PCSK9, otros hipolipemiantes, evolocumab, alirocumab

En general, las estatinas constituyen la piedra angular del tratamiento de la dislipemia, y los otros fármacos hipolipemiantes tienen su principal indicación cuando existe intolerancia a las estatinas o combinados con estas.

La combinación de estatinas con fibratos o niacina no incrementa el beneficio conseguido con la estatina sola. Sin embargo, en el estudio Improved Reduction of Outcomes: Vytorin Efficacy International Trial (IMPROVE-IT)^167^, la adición de ezetimiba a simvastatina produjo beneficios adicionales tras un síndrome coronario agudo y redujo el riesgo de la variable principal de resultado (compuesta por muerte cardiovascular, IAM, angina inestable, revascularización coronaria o ictus) en un 6% a los siete años de seguimiento. Un reciente análisis de subgrupos del IMPROVE-IT mostró que el mayor beneficio se observa en pacientes con diabetes y en pacientes de muy alto RV^168^.

Los estudios de intervención con los inhibidores de la PCSK9 han demostrado una reducción añadida de episodios vasculares no fatales, consistentes con sus efectos reductores en c-LDL y la duración de los ensayos clínicos^169^, ^170^.

En el *Further Cardiovascular Outcomes Research with PCSK9 Inhibition in Subjects with Elevated Risk* (FOURIER)^169^, el tratamiento con evolocumab^171^ en combinación con estatinas de moderada/alta intensidad en pacientes con EV establecida, consiguió una reducción del 15% en el objetivo primario compuesto por muerte, IAM, ictus, ingreso por angina inestable o revascularización coronaria en aproximadamente dos años de seguimiento, con independencia de la concentración basal de c-LDL.

Posteriormente, en el *Evaluation of Cardiovascular Outcomes After an Acute Coronary Syndrome During Treatment With Alirocumab* (ODYSSEY)^170^ se observó en pacientes con un síndrome coronario agudo reciente un descenso similar del objetivo primario compuesto por muerte coronaria, IAM no fatal, ictus isquémico fatal/no fatal o angina inestable que requiere hospitalización en el brazo de tratamiento con alirocumab, con un mayor beneficio absoluto en los pacientes con c-LDL > 100 mg/dL.

Diferentes subanálisis de los estudios FOURIER y ODYSSEY han aportado nuevas evidencias de los beneficios vasculares de los inhibidores de PCSK9 en diferentes situaciones clínicas como el paciente poli vascular, especialmente con enfermedad arterial periférica^172^, ^173^ y con concentraciones elevadas de Lp(a)^174^, ^175^. Cabe destacar que los inhibidores de PCSK9, también han demostrado efectos positivos en la composición y regresión de la placa de ateroma^171^, no incrementan la incidencia de diabetes, ni empeoran el metabolismo hidrocarbonado^176^, ni presentan efectos adversos en la función cognitiva^177^, ^178^ ni aumentan el riesgo de cataratas^179^ o de cáncer^180^. Sin embargo, su perfil de seguridad deberá confirmarse en estudios con un seguimiento a largo plazo. Actualmente, las indicaciones financiadas con cargo al Sistema Nacional de Salud de los inhibidores de la PCSK9 son en los siguientes grupos:

• Pacientes con EV establecida (cardiopatía isquémica, enfermedad cerebrovascular isquémica y enfermedad arterial periférica) no controlados con la dosis máxima tolerada de estatinas (c-LDL > 100 mg/dL).
• Pacientes con hipercolesterolemia familiar no controlados con la dosis máxima tolerada de estatinas (c-LDL > 100 mg/dL).
• Cualquiera de los pacientes de los grupos anteriores que sean intolerantes a las estatinas o en los que las estatinas están contraindicadas y cuyo nivel de c-LDL > 100 mg/dL.
• Los inhibidores del PCSK9 también incluyen aquellos que controlan la producción de la proteína PCSK9 vía *small interfering* RNA (Inclisiran). Todavía no existe evidencia de la eficacia del Inclisirán en la reducción de EV^181^, pero sí que inclisirán ha demostrado una reducción del 50-55% en los niveles de c-LDL cuando se aplica subcutáneamente cada seis meses. Las indicaciones de financiación aprobadas recientemente por el Sistema Nacional de Salud son coincidentes con las de los iPCSK9^182^.

#### Ácidos grasos omega-3

El efecto de los ácidos grasos omega-3 en la prevención vascular es controvertido, existiendo evidencias a favor y en contra^183^, ^184^, ^185^, ^186^, y se podrían dar diferentes explicaciones a cada una de las posiciones. En el *Reduction of Cardiovascular Events with Icosapent Ethyl–Intervention Trial* (REDUCE-IT)^187^ el uso de altas dosis de icosapento de etilo (4 g/día) en comparación con placebo se acompañó de una reducción significativa del RR del 25% de episodios vasculares graves en sujetos con EV estable o diabetes y concentraciones de c-LDL < 100 mg/dL y de triglicéridos entre 150 y 499 mg/dL.

Aunque el Sistema Nacional de Salud ha emitido resolución de no financiación, ha sido autorizado para reducir el riesgo de eventos cardiovasculares en pacientes adultos tratados con estatinas con riesgo cardiovascular alto con triglicéridos elevados (HTG) (≥ 150 mg/dL [≥ 1,7 mmol/L]) y una enfermedad cardiovascular diagnosticada, o diabetes y, al menos, otro factor de RV.

Antes de iniciar el tratamiento se debe descartar causas secundarias de HTG, optimizar la dieta, las medidas de estilo de vida y el control glucémico (en diabéticos); así como valorar individualizadamente los potenciales beneficios y riesgos (mayor incidencia de sangrado y FA, frente a placebo en el ensayo REDUCE-IT: 11,8 vs. 9,9% y 5,8 vs. 4,5%, respectivamente; siendo más frecuentes en pacientes con tratamiento antitrombótico y antecedente de FA^188^.

#### Ácido bempedoico

Otra opción terapéutica es el ácido bempedoico, que actúa inhibiendo la adenosina trifosfato-citrato liasa (ACL) y consecuentemente la biosíntesis del colesterol, lo que induce el aumento de la expresión de receptores LDL, incrementando el aclaramiento de c-LDL. A diferencia de las estatinas, su potencial efecto miotóxico es prácticamente inexistente. Ha sido aprobado recientemente como fármaco hipolipemiante en combinación con dieta, con estatinas o con otros fármacos hipolipemiantes en pacientes con hipercolesterolemia, dislipemia mixta, con intolerancia a estatinas o bien que estas estén contraindicadas. Se ha observado que el ácido bempedoico redujo significativamente el c-LDL en la semana 12 en comparación con placebo (-18,1%; IC 95% -16,1% a -20; p < 0,001)^189^. Recientemente se han publicado los resultados del estudio Cholesterol Lowering via Bempedoic Acid, an ACL-Inhibiting Regimen (CLEAR) outcomes donde se demuestra que el ácido bempedoico comparado con placebo en pacientes de alto riesgo cardiovascular e intolerantes a las estatinas reduce los eventos vasculares en un 13% (HR, 0,87; IC 95% 0,79-0,96; p = 0,004)^190^. Además, un subanálisis de los pacientes que no tenían EV mostró incluso mayor beneficio y reducción estadísticamente significativa del riesgo de muerte por causa cardiovascular^191^.

El Sistema Nacional de Salud restringe su financiación^192^, mediante visado, a pacientes con:

- Hipercolesterolemia familiar heterocigótica (HFHe) no controlados con la dosis máxima de estatina + ezetimiba, o con ezetimiba en caso de intolerancia o contraindicación a estatinas.
- Pacientes con EV aterosclerótica (EVA, que incluye la presencia de placas carotídeas) no controlados con la dosis máxima de estatina + ezetimiba, o con ezetimiba en caso de intolerancia o contraindicación a estatinas.
- Hay que tener en cuenta el incremento de niveles de ácido úrico y gota frente a placebo (5,5 vs. 1,5%), por lo que es preciso valorar su uso en pacientes con dichos antecedentes.

#### Inclisirán

Se trata de un RNA silenciador que inhibe la producción hepática de PCSK9. Su principal ventaja, frente los anticuerpos monoclonales que inhiben la actividad de esta proteasa, es su pauta de administración. Concretamente se administra una dosis subcutánea, seguida de otra a los tres meses y posteriormente cada seis meses.

### Objetivos de control

En las Figura 1, Figura 2, Figura 3 se exponen los algoritmos de cálculo del RV y objetivos de control de lípidos para personas aparentemente sanas, pacientes con diabetes y pacientes con EV aterosclerótica. El Comité Español para la Prevención Vascular (CEIPV) elaboró una adaptación a nuestro entorno de las Guías Europeas de Prevención Vascular^193^.

Se aconseja un abordaje escalonado de intensificación del tratamiento en personas aparentemente sanas de alto o muy alto RV, en pacientes con EV, y en pacientes diabéticos, teniendo en cuenta el RV, el beneficio del tratamiento, los modificadores del riesgo, las comorbilidades y las preferencias personales. En pacientes que han padecido una EV, para conseguir llegar a los objetivos terapéuticos de c-LDL lo antes posible, se recomienda pasar directamente a la etapa 2.

Se recomiendan las estatinas de alta intensidad en personas de muy alto riesgo o con EV, y si no se consiguen los objetivos de c-LDL, se debería añadir ezetimiba, y si aún no se consiguen los objetivos, se debería añadir un inhibidor del PCSK9. Si bien, como se ha comentado previamente, el ácido bempedoico, puede ser una opción previa añadido a estatinas y ezetimiba. Aunque esta recomendación está en línea con la consecución de los objetivos en dos etapas, es difícil conseguir reducciones ≥ 50% en c-LDL, excepto con las máximas dosis de atorvastatina y rosuvastatina. Las evidencias disponibles permiten cambiar la terminología de estatinas de alta potencia por la de terapias hipolipemiantes de alta intensidad^194^. De esta forma, la primera opción en pacientes de alto o muy alto RV podría ser utilizar dosis no máximas de estatinas (atorvastatina 40 mg o rosuvastatina 10 mg) asociadas a ezetimiba, que facilitan la consecución de los objetivos terapéuticos con una mejor tolerancia y adherencia. Se podría considerar añadir ácidos grasos n-3 (icosapento de etilo 2 x 2 g/día) al tratamiento con estatinas en pacientes de alto o muy alto RV con hipertrigliceridemia leve/moderada (a partir de niveles de triglicéridos superiores a 150 mg/dL). En la tabla 9 se recoge, de forma resumida, la calidad de la evidencia y la fuerza de la recomendación sobre el manejo de la dislipemia.

**Tabla 9:** Recomendaciones en dislipemia

Recomendación	Calidad de la evidencia	Fuerza de la recomendación
*Prueba diagnóstica*		
Las pruebas recomendadas para el cribado son: colesterol total, cLDL, cHDL y triglicéridos.	Moderada	Fuerte a favor
Periodicidad del cribado cada cuatro años	Baja	Fuerte a favor
Recomendada a partir de los 18 años en ambos sexos	Baja	Fuerte a favor

*Tratamiento farmacológico*		
Las estatinas son los fármacos de elección para el tratamiento de la dislipidemia	Alta	Fuerte a favor
En caso de no alcanzar los objetivos terapéuticos con estatinas a dosis plenas o de intolerancia a las estatinas se recomienda la ezetimiba	Moderada	Fuerte a favor
En caso de pacientes con enfermedad cardiovascular establecida o pacientes con HFHo o con HFHe, todos ellos con la dosis máxima tolerada de estatinas y un cLDL > 100 mg/dL, o bien cualquiera de esos pacientes con intolerancia a las estatinas y con cLDL > 100 mg/dL, se recomienda la utilización de inhibidores del PCSK9 (evolocumab o alirocumab) (criterios del Sistema Nacional de Salud)	Moderada	Fuerte a favor
Los objetivos y los criterios para introducir los fármacos hipolipemiantes dependen del cLDL y de la estratificación del riesgo cardiovascular	Moderada	Fuerte a favor

cHDL: colesterol unido a lipoproteínas de alta densidad; cLDL: colesterol unido a lipoproteínas de baja densidad; HFHo: hipercolesterolemia familiar homocigota; HFHe: hipercolesterolemia familiar heterocigota.

Elaboración propia.

## Obesidad

### Importancia epidemiológica y clínica del problema

El sobrepeso y la obesidad constituyen importantes problemas de salud tanto en personas jóvenes como en adultos. En la actualidad, se debe considerar la obesidad como una enfermedad crónica, compleja, multifactorial y recidivante; evitando los prejuicios y estigmas sociales, que pueden llegar a afectar la calidad de la atención médica. En este sentido, puede existir una tendencia a culpabilizar al paciente de falta de disciplina y voluntad, lo que tiene un impacto negativo en su bienestar emocional, psicológico y social^195^. Sabemos que la obesidad se relaciona con patologías como la hipertensión, la diabetes, patología osteomuscular, neurológica, pulmonar, gastrointestinal y hepática así como determinados tipos de cáncer: mama, colorrectal, riñón, hígado, ovario y mieloma^195^, ^196^, ^197^. La obesidad su fisiopatología influyen, por una parte, el acúmulo de tejido adiposo en diversos órganos y tejidos, provocando su disfunción por un proceso de lipotoxicidad y, por otra, cambios en el perfil secretor de adipoquinas en el tejido adiposo disfuncional, lo que contribuye al desarrollo de un proceso inflamatorio crónico de bajo grado. Este estado inflamatorio crónico junto a los procesos de lipotoxicidad, conducen a una alteración de los mecanismos homeostáticos y, en consecuencia, a los trastornos asociados con la enfermedad. La obesidad está asociada con un aumento en la morbilidad y mortalidad vascular en ambos sexos, incluyendo afecciones como la enfermedad coronaria, cerebrovascular, FA, insuficiencia cardiaca y muerte súbita cardiaca^198^, ^199^. A este mayor riesgo podría contribuir la influencia adversa de la obesidad sobre múltiples factores de RV, como la HTA, dislipemia, resistencia a la insulina (RI), síndrome de apneas-hipopneas del sueño y, particularmente, la DM2. Por todo ello, su manejo engloba desde los equipos de Atención Primaria a las Unidades hospitalarias multidisciplinares.

#### Prevalencia en España

La prevalencia del sobrepeso y la obesidad ha ido en aumento en las últimas décadas, en todo el mundo y en todos los grupos de edad, incluidos niños y adolescentes^200^. En 2023 se publicó el quinto Atlas Mundial de la Obesidad, con las estimaciones de los niveles nacionales de prevalencia de la obesidad y las tendencias de obesidad infantil y los niveles de obesidad en el mundo^201^. En este atlas se publica también el impacto económico del sobrepeso y la obesidad y proyectan cómo cambiará el impacto económico para 2035. Las estimaciones presentadas en este Atlas sugieren que, si se mantienen las tendencias actuales, el sobrepeso y la obesidad costarán a la economía mundial más de 4 billones de dólares en 2035, casi el 3% del actual producto interior bruto (PIB) mundial. En España, los datos aportados por el Atlas sugieren que en 2035 la prevalencia de obesidad será muy alta, del orden del 37% en adultos, con una previsión de aumento de la incidencia anual de obesidad en adultos desde el año 2020 al año 2035 del 1,9%, lo que lo sitúa en nivel medio. En niños, se prevé un aumento de obesidad alto, del 2,5% anual de los años 2020 a 2035, siguiendo la tendencia a nivel mundial. Respecto a las intervenciones que se llevan a cabo en nuestro país para frenar esta tendencia, España ocupa el puesto 51 de 183^6^. En un estudio español reciente^196^, ^197^ se ha estimado una alta prevalencia de obesidad en adultos de 65 y más años, concretamente del 40,1% en mujeres y del 32% en hombres. Esta prevalencia de obesidad en personas de 65 años o más aumenta en personas con menor nivel educativo y en la región sur de España respecto al este, norte-este y centro.

### Tratamiento

#### Medidas no farmacológicas

El abordaje no farmacológico y las intervenciones sobre estilos de vida deben estar presentes en todo el proceso de manejo de la obesidad. Idealmente estas estrategias deberían de llevarse a cabo por parte de un equipo multidisciplinar y deberían incluir terapia conductual, prescripción de dieta y de ejercicio.

La terapia conductual es uno de los pilares fundamentales en los programas de intervención en personas con obesidad. El objetivo de la intervención conductual es incrementar la autoestima y el autocontrol de los pacientes con obesidad con el fin de adoptar y mantener estilos de vida saludables para la mejora de la salud y calidad de vida. Los cambios en dieta y ejercicio físico son difíciles de implementar y mantener a largo plazo; y se han identificado múltiples barreras que limitan el cambio conductual, como son la baja motivación, el tiempo limitado, los pensamientos intrusivos de negación y culpabilidad, el estrés o las presión social y económica, entre otros^202^. Las intervenciones conductuales se plantean dependiendo de las características y necesidades del paciente y presentan distintos formatos que incluyen visitas de tipo individual o grupal, presencial o a distancia o mediante apoyo en plataformas web, aplicaciones u otros recursos digitales^203^.

La prescripción de dieta debe ser una decisión compartida con el paciente e individualizada que permita la elección de unas pautas que puedan adaptarse a los hábitos, cultura y horarios del paciente, elementos que serán clave en la adherencia a largo plazo. La reducción de la ingesta calórica total debe ser el componente principal de cualquier intervención dietética y el objetivo inicial será conseguir una reducción energética en la dieta de 500-1.000 kcal al día. Esta práctica puede producir una pérdida de peso de entre 0,5 y 1 kg/semana, equivalentes a más de un 5% de pérdida ponderal en un periodo promedio de seis meses^204^.

El ejercicio físico debe buscar potenciar la pérdida de peso conseguida con los cambios en la dieta y además aporta beneficios a nivel vascular y de salud mental. En la prescripción de ejercicio siempre hay que tener en cuenta las características del paciente, comorbilidades y preferencias. Es importante que los ejercicios prescritos se adecúen a las limitaciones físicas de los pacientes, así como que el paciente pueda elegir aquel que se adapte más a sus preferencias.

La intervención inicial en prescripción de ejercicio físico debe ir dirigida a reducir el sedentarismo y después se debería prescribir ejercicio físico dirigido, incluyendo tanto ejercicio aeróbico como de fuerza o resistencia.

En la prescripción de actividad física aeróbica, las guías clínicas sugieren iniciar de manera progresiva en cuanto a tiempo e intensidad sesiones con el objetivo de llegar a realizar 150 minutos de actividad moderada semanales repartidos en tres a cinco sesiones semanales. Estas recomendaciones para la fase inicial de pérdida de peso se aumentarían hasta 300 minutos por semana en la fase de mantenimiento^204^, ^205^. La combinación de ejercicio aeróbico con tandas de ejercicios de fuerza que incluyan series de repeticiones en distintos grupos musculares permite potenciar la pérdida de masa grasa y también la preservación de la masa muscular.

#### Tratamiento farmacológico

Se recomienda iniciar el tratamiento farmacológico para la obesidad cuando la alimentación saludable y la actividad física por sí solas han sido ineficaces, insuficientes o sin un beneficio sostenido en el objetivo de disminuir el peso y optimizar la salud. El tratamiento farmacológico está indicado en aquellos pacientes con obesidad (IMC ≥ 30 kg/m^2^) o con sobrepeso grado II (IMC ≥ 27 kg/m^2^) si existen comorbilidades mayores asociadas.

En España existen en la actualidad cuatro fármacos autorizados y comercializados para el control de la obesidad:

- Orlistat, 120 mg vía oral, tres veces al día (tomado antes, durante o hasta una hora después de las comidas). Está indicado junto con una dieta hipocalórica para el tratamiento de personas con un IMC mayor o igual a 30 kg/m^2^, o pacientes con sobrepeso grado II (IMC ≥ 27 kg/m^2^) y comorbilidades. Si en una comida no se toma o no contiene grasa, debe omitirse la dosis de orlistat. El tratamiento con orlistat debe interrumpirse después de 12 semanas si los pacientes no han perdido al menos el 5% del peso corporal al inicio del tratamiento^206^. Los datos de eficacia disponibles para orlistat 120 mg tres veces al día muestra que un 54% de tratados con el fármaco vs. 33% con el placebo lograron una pérdida ≥ 5%; llegando a ser ≥ 10% en el 26% vs. 14% de pacientes (diferencias del 21 y 12%). La pérdida media de peso fue del 2,9%^207^, ^208^.
- Liraglutida 3,0 mg: vía subcutánea, una vez al día. La dosis para iniciar el tratamiento con liraglutida es de 0,6 mg al día, con un aumento de la dosis de 0,6 mg cada semana hasta alcanzar el objetivo de 3,0 mg. o la dosis máxima tolerada. La asociación de liraglutida 3,0 mg junto con dieta y ejercicio logró una pérdida de peso del 8,0% al año, en comparación con el 2,6% con placebo. El 63,2% de los pacientes tratados con liraglutida perdieron ≥ 5% del peso al año, en comparación con el 27,1% en el grupo de placebo. El porcentaje de pacientes que perdieron más del 10% de su peso fue del 33,1% con liraglutida 3,0 y del 10,6% con placebo^209^, ^210^.
- Semaglutida 2,4 mg: vía subcutánea, una vez a la semana, actúa sobre los receptores de los GLP1 con efectos sobre el páncreas (más importantes en personas con DM2), en el sistema nervioso central, regulando el apetito y la ingesta, y en el aparato digestivo, enlenteciendo el vaciado gástrico. De esta forma se favorece la pérdida de peso. El tratamiento se inicia con 0,25 mg por semana, aumentando la dosis cada cuatro semanas a 0,5 mg, 1 mg, 1,7 mg, y finalmente, la dosis máxima de 2,4 mg por semana. En adultos sin diabetes, semaglutida 2,4 mg, junto con modificaciones en la alimentación y ejercicio, consigue una pérdida de peso de 14,9%, en comparación con 2,4% con placebo. Pérdidas ≥ 5% del peso corporal se consiguen en el 86,4% de los pacientes con semaglutida frente al 31,5% con placebo El 69,1% de los pacientes pierde ≥ 10% del peso corporal con semaglutida y el 12% de los pacientes con placebo^211^. El ensayo STEP 8 comparó la semaglutida (dosis de 2,4 mg semanales) con liraglutida (3 mg diarios) en pacientes con obesidad, IMC medio de 37,5 ± 6,8 kg/m^2^. La pérdida de peso corporal total fue mayor con semaglutida que con liraglutida, con una diferencia media del porcentaje de pérdida de peso corporal total del 9,4 ± 6,8-12,0% a las 68 semanas^212^. Más recientemente, el estudio Semaglutide Effects on Heart Disease and Stroke in Patients with Overweight or Obesity (SELECT) demostró una reducción significativa de complicaciones cardiovasculares y mortalidad por cualquier causa en pacientes con IMC > 27 kg/m^2^ sin diabetes tratados con semaglutide (2,4 mg semanales) frente al placebo^213^.

Como para cualquier otra medicación, es necesario estar atentos a la información de las agencias de medicamentos sobre seguridad. Posibles efectos adversos, incluso raros, recogidos por la experiencia en estudios observacionales, requieren de estudios más rigurosos para establecer su causalidad^214^.

- Tirzepatida: vía subcutánea. Dosis inicial de 2,5 mg una vez por semana. Después de cuatro semanas, se debe aumentar a 5 mg una vez a la semana, pudiéndose titular hasta una dosis máxima de 15 mg. Tirzepatida es un agonista incretínico dual (de GLP-1 y GIP). En el estudio Study of Tirzepatide in Participants With Type 2 Diabetes Not Controlled With Diet and Exercise Alone (SURPASS) 2, un ensayo clínico aleatorizado en pacientes con DM2, se evidenció que, tras 40 semanas de tratamiento, la reducción del peso con todas las dosis de tirzepatida (5, 10 y 15 mg/semana) era mayor que la lograda con 1 mg/semana de semaglutida. Así la pérdida de peso era de 7,6, 9,3 y 11,2 kg respectivamente, con las dosis de 5, 10, y 15 mg de tirzepatida, y de 5,7 kg con la dosis de 1 mg de semaglutida. En cuanto al porcentaje de pérdida de peso, la reducción era del 8,5, 11,0 y 13,0% con cada una de las dosis de tirzepatida y del 6,7% con semaglutida^215^. Al tratarse de un estudio de diabetes, la dosis de comparación de semaglutida empleada era de 1 mg/semana y no de 2,4 mg/semana, la dosis aprobada para obesidad^216^. En el ensayo Tirzepatide once weekly for the treatment of obesity in people with type 2 diabetes (SURMOUNT) 1 (dentro del programa de desarrollo del fármaco para pacientes con obesidad, SURMOUNT) se compararon diferentes dosis de tirzepatida (5, 10, y 15 mg/semana) con placebo en pacientes con obesidad y sin DM2. Tras 72 semanas, la pérdida de peso media resultó ser significativamente mayor con las diferentes dosis de tirzepatida: 15, 19 y 21% respectivamente, en comparación con el 3% en el grupo placebo^216^.

#### Cirugía bariátrica

Los criterios para la indicación de la cirugía bariátrica (CB) dependen fundamentalmente del nivel de IMC, pero también de otros factores.

a) Criterios de IMC:
  - IMC ≥ 40 kg/m^2^. En estos pacientes el beneficio de la CB presenta una evidencia alta^217^, ^218^.
  - IMC ≥ 35 y < 40 kg/m^2^ con una o más complicaciones graves: DM2 (evidencia alta)^219^, ^220^, alto riesgo de DM2, HTA de difícil control, hígado graso no alcohólico, síndrome de apnea obstructiva del sueño (evidencia moderada), osteoartritis de rodilla y cadera, o incontinencia urinaria. Otras complicaciones que pueden considerarse son: obesidad con hipoventilación, hipertensión intracraneal idiopática, reflujo gastroesofágico, estasis venoso severo, movilidad reducida debido a obesidad; y la alteración importante de la calidad de vida (evidencia baja). También se incluyen el síndrome de ovario poliquístico que cause infertilidad, esteatohepatitis con sugerencia de fibrosis 3-4, o pacientes en los que la pérdida ponderal sea prioritaria.
  - IMC 30-34,9 kg/m^2^ donde la pérdida ponderal sea prioritaria: DM2 con mal control a pesar de tratamiento intensificado y presencia de otras complicaciones graves, o en sujetos no diabéticos con complicaciones graves que no se controlen adecuadamente y supongan una disminución importante de la calidad de vida (evidencia moderada). Debe recordarse que la CB no está exenta de riesgos y complicaciones^221^, pero en la mayoría de pacientes con este IMC, la obesidad se seguirá tratando en Atención Primaria y serán remitidos a la especialidad correspondiente para valorar si es candidato a CB en función de la patología subyacente: DM2, obesidad secundaria (enfermedad de Cushing, acromegalia) o sospecha de obesidad sindrómica (obesidad desde la infancia, asociación con hipogonadismo, hiperfagia exagerada, facies característica), indicación de prótesis de cadera/rodilla, trasplantes, síndrome de apnea-hipopnea del sueño, infertilidad.
b) Otros factores. Además del criterio de IMC, el paciente adulto candidato a CB debe cumplir los siguientes requisitos.
  - Edad entre 18 y 65 años. La CB en pacientes con edades más extremas deberá evaluarse individualmente y según la experiencia del centro.
  - Respuesta inadecuada al tratamiento médico previo en Atención Primaria. Antes de remitir un paciente a valoración para CB deben haberse agotado los recursos de tratamiento habituales.
  - Buena motivación del paciente y capacidad de adherencia a los cambios de estilos de vida necesarios en el post operatorio inmediato y en el seguimiento posterior.
  - Estabilidad psicológica. Los candidatos deben ser evaluados para identificar, y en su caso indicar, la necesidad de seguimiento por la unidad de salud mental para facilitar el proceso de adaptación a los cambios médicos, psicológicos y sociales tras la cirugía.
  - Ausencia de contraindicaciones importantes: muy alto riesgo quirúrgico, expectativa de vida limitada por cualquier enfermedad, cirrosis severa o abuso de alcohol y/o otras drogas.
  - Ausencia de enfermedad endocrinológica tratable como causa de la obesidad.
  - Compromiso de no gestación durante el año posterior a la CB.

## Diabetes

### Importancia epidemiológica y clínica del problema

En el mundo, 537 millones de personas entre 20-79 años padecen DM, un 10,5% de la población^222^. La DM2 es el tipo más común de diabetes y representa el 90% de los casos de diabetes en todo el mundo^222^. En España la prevalencia de DM en población mayor de 18 años se sitúa en el 10-13%, de la cual, solo el 54% es conocida (4,7-6% de prevalencia de DM no diagnosticada)^223^, ^224^. El estudio sobre nutrición y riesgo cardiovascular en España (ENRICA)^225^ realizado con glucemia basal, observó una prevalencia de 6,9%, mayoritariamente conocida (79,5%); y un estudio poblacional en Cataluña^226^, revisando 286.791 historias clínicas, observó una prevalencia de diabetes registrada del 7,6%. Un metaanálisis de 55 estudios europeos^227^ ha descrito un 5,46% (IC 95% 4,7-6,1) de diabetes no diagnosticada. Por tanto, los datos concuerdan que la prevalencia de DM se sitúa en torno al 12% y la diabetes no conocida se sitúa en torno al 5-6% de la población (40-50% del total).

En relación a la incidencia de DM2 en población española mayor de 18 años, es de 11,6 casos/1.000 personas-año (IC 95% 11,1–12,1), con una incidencia de DM no conocida de 7,9 casos/1.000 personas-año (IC 95% 5,3–8,1)^228^. Los factores de riesgo asociados a una mayor incidencia de DM2, son: glucemia basal alterada (GBA), tolerancia alterada a la glucosa (TAG), presencia de ambas (TAG + GBA), obesidad central (perímetro de cintura ≥ 94 cm en hombres y ≥ 80 en mujeres), obesidad (IMC > 30 kg/m^2^), ser varón, y tener historia familiar de DM^228^.

#### Comorbilidad

La DM2 es una patología que suele asociarse con otras comorbilidades que aumentan el riesgo cardiovascular^226^: HTA (77,8%), colesterol LDL > 100 mg/dL (62%), obesidad (45,4%), tabaquismo (15,4%) o prevención secundaria cardiovascular (23%). También se ha observado que las personas con nivel socioeconómico bajo presentan más prevalencia de comorbilidades en comparación con las de nivel más alto: cardiopatía isquémica (24 vs. 21%), enfermedad pulmonar obstructiva crónica (EPOC) (11 vs. 4%), dolor crónico (28 vs. 14%), depresión (21 vs. 13%), ansiedad (10 vs. 7%)^229^. Por ello, el abordaje de la DM2 debe ser integral y el médico de familia desempeña un papel fundamental en esta patología, por su visión integral del paciente y el abordaje bio-psico-social.

### Grado de control

Aunque en la mayoría de pacientes se recomienda un objetivo de hemoglobina glicosilada A1c (HbA1c) < 7% (para pruebas con valor de normalidad 5,7%), es preferible individualizar el objetivo, recomendando HbA1c < 6,5% en pacientes jóvenes sin complicaciones y con buena expectativa de vida, HbA1c < 8% en pacientes mayores con complicaciones y años de evolución de la DM2, incluso HbA1c < 9% o simplemente ausencia de síntomas en pacientes con expectativa de vida muy limitada^230^, ^231^. Una extensión del estudio United Kingdom Prospective Diabetes Study (UKPDS) a 25 años de seguimiento ha evidenciado que lograr valores cercanos a la normoglucemia inmediatamente después del diagnóstico podría ser esencial para minimizar el riesgo de por vida de complicaciones relacionadas con la diabetes. Así, a pesar de que los pacientes, una vez finalizado el estudio, recibieron una práctica clínica habitual, en el grupo con tratamiento intensivo en el momento del diagnóstico de diabetes, se observó, una reducción 10% (IC 95% 2-17; p = 0,015) para muerte por cualquier causa, 17% (IC 95% 6-26; p = 0,002) para IAM, y 26% (IC 95%14-36; p = 0,000) para enfermedad microvascular^232^. En el grupo de tratamiento intensivo con metformina se observó una reducción 20% (IC 95% 5-32; p = 0,010) para muerte por cualquier causa y 31% (IC 95% 12-46; p = 0,003) para IAM. A este beneficio a largo plazo del buen control inicial en el momento del diagnóstico se le denomina efecto legado (*legacy effect*). Por ello, se recomienda intentar conseguir una HbA1c < 6,5% en el momento del diagnóstico.

En España, el estudio ENRICA^233^ describe un 69% de pacientes con HbA1c < 7%, y en otro estudio nacional^234^ el porcentaje de control fue de 56,1% sin individualizar objetivos de HbA1c y 60,5% individualizando. Otro gran estudio poblacional en práctica clínica^235^, en el periodo 2007-2013, analizando respectivamente 257.072 y 343.969 personas con DM2, describe que la proporción de pacientes con HbA1c < de 7% fue de 54,9% en 2007 y de 55,2% en 2013, sin observar mejoras en el periodo de tiempo analizado. A nivel internacional, comparando el grado de control (HbA1c < 7%) en AP, en 2016, en ocho países se observa amplia variabilidad, desde el 42% (Reino Unido) o 47,3% (Australia) hasta 54,3% (Italia) o 59,1% (Francia)^236^. En el estudio realizado en España sobre Diabetes (DIAMOND2), el más reciente estudio español (año 2022) realizado en AP, se observa un 57,7% de control que aumenta al 62,3% si se aplican criterios individualizados de control^237^. Puede concluirse, que alrededor del 40-50% de las personas con diabetes no presentan buen control glucémico. Se ha descrito también una importante variabilidad entre médicos de una misma área de salud, variando entre 30 y 80% el grado de control entre 113 médicos de familia de la misma área, así como en la misma Comunidad Autónoma entre las diferentes áreas de salud^238^.

Si se analiza el control integral de tres factores de riesgo, solo un 12,9% de los pacientes diabéticos en prevención primaria cardiovascular, tenía buen control (HA1c ≤ 7%, PA ≤ 130/80 mmHg y c-LDL < 130 mg/dL) y un 12,1% en prevención secundaria (HA1c ≤ 7%, PA ≤ 130/80 mmHg y c-LDL < 100 mg/dL)^226^. Esta dura situación requiere de nuevas estrategias que consigan mejores resultados por lo que es preciso realizar estudios para analizar las causas de este pobre control en factores de riesgo conocidos y para los que hay tratamientos eficaces.

### Cribado y pruebas diagnósticas

#### Diagnóstico de prediabetes y diabetes

El término prediabetes, tal como se definen actualmente, también denominado hiperglucemia intermedia (HI), incluye la GBA y la intolerancia a la glucosa (ITG), y son categorías de riesgo que pueden evolucionar a DM2. Existen pruebas que confirman que la DM2 puede prevenirse modificando el estilo de vida y en algunos casos con medicación, en personas con ITG diagnosticada mediante glucosa plasmática (GP) 2 h después de una sobre carga oral de glucosa (SOG) de 75 g^239^. Sin embargo, la Federación Internacional de Diabetes (IDF) propone cambiar la SOG de 2 h por la SOG de 1 h con un punto de corte de glucemia ≥ 155 mg/dL pues este valor es altamente predictivo para detectar la progresión a DM2, complicaciones micro y macrovasculares, apnea obstructiva del sueño, DM relacionada con fibrosis quística, enfermedad hepática esteatósica asociada a disfunción metabólica (MAFLD) y mortalidad en individuos con factores de riesgo. El resultado obtenido tras la SOG de 1 h ≥ 209 mg/dL también es diagnóstico de DM2. El PAPPS asume esta recomendación^240^.

El diagnóstico de prediabetes y diabetes se puede realizar mediante la medición de la glucemia basal en ayunas, la medición a la 1 h tras SOG o mediante la medición de la HbA1c (tabla 10).

**Tabla 10:** Criterios diagnósticos de prediabetes y diabetes

	Glucemia basal (mg/dL)	HbA1c (%)	Glucemia al azar (mg/dL) + sintomas	SOG-1 h (mg/dL)
Prediabetes	100-125 (1)	5,7-6,4	NC	≥ 155 (2)
Diabetes mellitus	≥ 126	≥ 6,5	≥ 200	≥ 209

HbA1c: hemoglobina glicosilada A1c; NC: no considerado; SOG-1 h: sobrecarga oral de glucosa o medición de glucemia a 1 h tras 75 g de glucosa.

(1) Glucemia basal alterada (GBA)

(2) Tolerancia alterada a la glucosa (TAG).

Elaboración propia. Modificada de^245^.

La HbA1c, comparada con la glucemia basal, proporciona pequeñas mejoras en la predicción del RV en pacientes no diagnosticados de diabetes, y en las personas con diabetes la HbA1c presenta una asociación más fuerte con el riesgo de microangiopatía (retinopatía y nefropatía) y EV que la media de las glucemias^241^.

Para el diagnóstico de diabetes son necesarios dos valores alterados de la misma prueba en momentos diferentes (dos glucemias alteradas en dos analíticas diferentes) o de diferentes pruebas en el mismo momento (p. ej., HbA1c y glucemia alteradas en la misma analítica).

#### Cribado

Existen varias estrategias para el cribado de la DM2, como el cribado poblacional general o en población de riesgo o el oportunista del paciente que acude a consulta. Puede realizarse mediante la determinación de la glucemia, o mediante cuestionarios o escalas de riesgo. Una revisión sistemática concluyó que el cribado en población general no reduce la mortalidad en un seguimiento a 10 años, y subraya la necesidad de más estudios que determinen la efectividad del tratamiento de la diabetes detectada por cribado^242^. El estudio Anglo-Danish-Dutch Study of Intensive Treatment in People with Screen-Detected Diabetes in Primary Care (ADDITION) observó que el cribado frente al no cribado no fue superior en la reducción del riesgo de mortalidad total (HR: 1,06; IC 95%, 0,90-1,25), mortalidad cardiovascular (HR: 1,02; IC 95%, 0,75-1,38) o mortalidad relacionada con la diabetes (HR: 1,26; IC 95%, 0,75-2,10)^243^. Sin embargo, algunas guías recomiendan el cribado en grupos poblacionales amplios: de 35 a 70 años con un IMC de 25 o más (USPSTF, 2021)^244^; o de 35 años o más o un IMC de 25 o más y uno o más más factores de riesgo^245^. Según estos criterios debería realizarse cribado entre 43-82% de la población general^246^. Otra revisión sistemática^247^, que incluyó 138 estudios, concluye que la HbA1c no tiene suficiente sensibilidad ni especificidad como prueba de cribado y que la glucemia en ayunas es específica pero poco sensible (tabla 11).

**Tabla 11:** Validez de las pruebas de cribado en diabetes tipo 2

	Glucemia basal (mg/dL)		HbA1c (%)	
	Todos los estudios (n = 23)	Estudios con bajo riesgo de sesgo	Todos los estudios (n = 21)	Estudios con bajo riesgo de sesgo
Sensibilidad	0,25 (0,19-0,32)	0,24 (0,17-0,32)	0,49 (0,40-0,58)	0,47 (0,37-0,58)
Especificidad	0,94 (0,92-0,96)	0,95 (0,93-0,97)	0,79 (0,73-0,84)	0,81 (0,74-0,86)
Falsos negativos	0,75 (0,68-0,81)	0,76 (0,86-0,83)	0,51 (0,42-0,60)	0,53 (0,42-0,63)
Falsos positivos	0,06 (0,04-0,08)	0,05 (0,03-0,07)	0,21 (0,16-0,27)	0,19 (0,14-0,26)
Área bajo la curva	0,72	0,73	0,71	0,71

Elaboración propia. Modificada de^247^.

Las diferentes pruebas empleadas en el cribado identifican poblaciones muy diferentes y sus indicadores de validez no son buenos, por lo que muchas personas estarán innecesariamente tratadas o falsamente despreocupadas, dependiendo de la prueba empleada. Al no existir una evidencia clara a favor del cribado mediante glucemia, no puede tampoco recomendarse una periodicidad.

Se han publicado evidencias de que el tratamiento (estilo de vida o farmacológico) sobre los estados de pre diabetes diagnosticados por cribado se asocia a reducción del riesgo de progresión a diabetes^248^, ^249^, ^250^, ^251^, ^252^, ^253^, ^254^. En una revisión Cochrane^254^, no se demuestra que la dieta sola o el ejercicio físico por sí solo, modifiquen la incidencia de diabetes o sus complicaciones en pacientes con riesgo de desarrollar diabetes. Pero la adición de dieta y ejercicio si previene o retrasa la incidencia de DM2 en pacientes con TAG. La metformina comparada con el placebo o la dieta y el ejercicio redujo o retrasó el riesgo de DM2 en personas con mayor riesgo de desarrollar DM2 (evidencia de calidad moderada)^254^. Sin embargo, la metformina comparada con la dieta y el ejercicio intensivos no redujo ni retrasó el riesgo de DM2 (evidencia de calidad moderada). Asimismo, la combinación de metformina, dieta intensiva y ejercicio en comparación con la dieta intensiva y el ejercicio no mostró una ventaja con respecto al desarrollo de la DM2 (evidencia de muy baja calidad). Los datos sobre resultados importantes para los pacientes, como la mortalidad, las complicaciones diabéticas macro y micro vasculares y la calidad de vida relacionada con la salud, fueron escasos.

Con estas evidencias no puede recomendarse el cribado poblacional de DM2 mediante glucemia, salvo en pacientes con factores de riesgo asociados o con antecedentes familiares de diabetes, recomendando realizar la prueba aprovechando la petición de una analítica por cualquier motivo. Por ejemplo, la glucemia suele añadirse habitualmente en el contexto de la detección o seguimiento de otros FRV.

Sin embargo, el uso de escalas o cuestionarios, por su sencillez y eficiencia sí podría recomendarse para identificar personas de riesgo. El test Finnish Diabetes Risk Score (FINDRISC) permite identificar sujetos de alto riesgo de padecer DM2 si la puntuación es mayor de 15 puntos evitando la glucemia como prueba de cribado (estudio Diabetes in Europe—Prevention using Lifestyle, Physical Activity and Nutritional intervention [DE-PLAN])^255^ y ha sido validado en español^256^.

## Tratamiento

### Medidas no farmacológicas

#### Alimentación

Los programas de educación/consejo y los programas de sustitución de alimentos en AP reducen la HbA1c en pacientes con DM2 entre -0,37 (IC 95% -0,57 -0,17) y -0,54 (IC 95% -075,-0,32)^257^. En un metaanálisis incluyendo 52 ECA que comparaban nueve enfoques dietéticos (bajo en grasas, vegetariano, mediterráneo, alto en proteínas, moderado en carbohidratos, bajo en carbohidratos, control, bajo índice glucémico/carga glucémica y dieta paleolítica) que incluían a 5.360 pacientes con DM2, la dieta mediterránea fue el enfoque dietético más eficaz para controlar la dislipemia diabética^258^. Se recomienda el uso del cuestionario cualitativo de 14 ítems sobre la dieta mediterránea del estudio Prevención con Dieta Mediterranea (PREDIMED)^259^ (tabla 12). Además, diferentes estudios prospectivos muestran una asociación beneficiosa del consumo de aceite de oliva con la prevención de la enfermedad CV, la DM2 y la mortalidad por todas las causas^260^.

**Tabla 12:** Cuestionario de valoración del grado de adherencia a la dieta mediterránea

Criterio	Valoración	Puntos
1. ¿Usa usted el aceite de oliva como principal grasa para cocinar?	Sí = 1 punto	
2. ¿Cuánto aceite de oliva consume en total al día (incluyendo el usado para freír, comidas fuera de casa, ensaladas, etc.)?	4 o más cucharadas = 1 punto	
3. ¿Cuántas raciones de verdura u hortalizas consume al día? (las guarniciones o acompañamientos = 1/2 ración) 1 ración = 200 g	2 o más (al menos una de ellas en ensalada o crudas) = 1 punto	
4. ¿Cuántas piezas de fruta (incluyendo zumo natural) consume al día?	menos de 1 al día = 1 punto	
5. ¿Cuántas raciones de carnes rojas, hamburguesas, salchichas o embutidos consume al día? (ración: 100-150 g)	menos de 1 al día = 1 punto	
6. ¿Cuántas raciones de mantequilla, margarina o nata consume al día? (porción individual: 12 g)	menos de 1 al día = 1 punto	
7. ¿Cuántas bebidas carbonatadas y/o azucaradas (refrescos, colas, tónicas, *bitter*) consume al día?	menos de 1 al día = 1 punto	
8. ¿Bebe usted vino? ¿Cuánto consume a la semana?	7 o más vasos a la semana = 1 punto	
9. ¿Cuántas raciones de legumbres consume a la semana? (1 plato o ración de 150 g)	3 o más a la semana = 1 punto	
10. ¿Cuántas raciones de pescado-mariscos consume a la semana? (1 plato pieza o ración: 100- 150 de pescado o 4-5 piezas o 200 g de marisco)	3 o más a la semana = 1 punto	
11. ¿Cuántas veces consume repostería comercial (no casera) como galletas, flanes, dulces o pasteles a la semana?	menos de 2 a la semana = 1 punto	
12. ¿Cuántas veces consume frutos secos a la semana? (ración 30 g)	3 o más a la semana = 1 punto	
13. ¿Consume usted preferentemente carne de pollo, pavo o conejo en vez de ternera, cerdo, hamburguesas o salchichas? (carne de pollo: 1 pieza o ración de 100-150 g)	Sí = 1 punto	
14. ¿Cuántas veces a la semana consume los vegetales cocinados, la pasta, arroz u otros platos aderezados con salsa de tomate, ajo, cebolla o puerro elaborada a fuego lento con aceite de oliva (sofrito)?	2 o más a la semana = 1 punto	

Tomado de ^259^

#### Ejercicio

Las directrices internacionales actuales recomiendan tanto ejercicios aeróbicos, como de resistencia y preferentemente combinados para mejorar el control glucémico, de la PA y de los lípidos^261^, ^262^. Se recomiendan 150 minutos o más a la semana de ejercicio aeróbico. Los beneficios del ejercicio disminuyen a las 72 horas de dejar de practicarlo^263^, por lo que se recomienda realizarlo al menos tres días por semana, a días alternos. El tipo de ejercicio debe adaptarse a las circunstancias de cada paciente (físicas, económicas). Se recomienda reducir la cantidad de tiempo dedicado a conductas sedentarias a un máximo de 30 minutos, poniéndose de pie brevemente, caminando o realizando otras actividades físicas ligeras^264^, ^265^. Participar en actividades de ocio y evitar periodos prolongados de sedentarismo puede ayudar a prevenir la DM2 en las personas de riesgo y también puede ayudar a controlar la glucemia en las personas diabéticas. El ejercicio es tan importante como la alimentación saludable y ambos deben recomendarse en cualquier estadio de evolución de la diabetes.

#### Tratamiento farmacológico

Se dispone de al menos siete grupos farmacológicos con mecanismos de acción diferentes cuyos principales beneficios y riesgos se detallan en la tabla 13. El fármaco ideal debería ser eficaz en la reducción de la glucemia, pero además no producir hipoglucemias, favorecer la reducción de peso y la PA y reducir el riesgo de los eventos vasculares, y de progresión de la enfermedad renal; así como de la mortalidad cardiovascular y total. Los tres grupos que más se acercan a estos requerimientos son la metformina, los inhibidores del cotransportador de sodio-glucosa tipo 2 y los agonistas del péptido similar al glucagón tipo 1 (arGLP1) y deben ser los fármacos de uso prioritario en el tratamiento de la DM2^266^. La mayoría de pacientes requerirán de una combinación de estos fármacos para alcanzar un buen control. Las otras familias terapéuticas quedarían indicadas en caso de contraindicación, eventos adversos o intolerancia a los fármacos descritos, así como la insulina cuando, por la propia progresión de la enfermedad, se requiera su empleo para conseguir un control adecuado.

**Tabla 13:** Valoración de los fármacos para el tratamiento de la diabetes

	Reducción HbA1c	Seguridad (hipoglucemias)	Reducción peso	Coste reducido	Reducción eventos CV	Reducción nefropatía	Reducción mortalidad
Metformina	++	+	+	++	+	-	+
iSGLT2	++	+	++	-	++	++	++
arGLP1	+++	+	+++	–	++	+	++
arGLP1-GIP	++++	+	++++	–	++	+	++
Insulina	+++	–	-	–	-	±	±
iDPP4	+	+	±	-	±	±	±
Sulfonilureas/ Glinidas	++	–	-	++	-	±	-
Pioglitazona	++	+	–	+	+	±	+

+ efecto positivo; **-** efecto negativo; ± efecto neutro; iSGLT2: *sodium-glucose cotransporter-2 inhibitors* o inhibidores del cotransportador de sodio-glucosa tipo 2; arGLP1: *glucagón-like peptid-1 receptor agonists* o agonistas del péptido similar al glucagón tipo 1; GIP: *gastric inhibitory polypeptide* o polipéptido insulinotrópico dependiente de glucosa; arGLP1-GIP: agonista dual del receptor de polipéptido insulinotrópico dependiente de glucosa (GIP) y de GLP-1; iDPP4: inhibidores de la dipeptidil peptidasa 4.

Los iSGLT2 tienen, además, un efecto protector sobre la insuficiencia cardiaca.

Elaboración propia.

En un metaanálisis incluyendo 38 ECA con siete clases diferentes de tratamientos reductores de la glucosa, los ISGLT2 y los arGLP1 mostraron una reducción del riesgo de un objetivo compuesto de tres (3P-MACE), MACE (ISGLT2 [RR 0,90; IC 95% 0,84-0,96; número de pacientes necesarios a tratar {NNT}, 59], arGLP1 [RR 0,88; IC 95% 0,83-0,93; número de pacientes necesarios a tratar {NNT}, 50]), muerte cardiovascular, mortalidad por todas las causas, resultado compuesto renal y macroalbuminuria. En el mismo estudio, los iSGLT2 también mostraron una reducción del riesgo de hospitalización por insuficiencia cardiaca, enfermedad renal terminal, lesión renal aguda, duplicación de la creatinina sérica y disminución de la tasa de filtración glomerular. Los arGLP1 se asociaron a un menor riesgo de ictus, mientras que el uso de glitazona se asoció a un mayor riesgo de insuficiencia cardiaca^267^. Otro estudio en Inglaterra incluyendo 75.739 adultos con DM2 que iniciaron tratamiento antidiabético oral de segunda línea con una sulfonilurea, un inhibidor de la DPP-4 o un inhibidor de la SGLT-2 añadido a metformina, descubrió que los iSGLT-2 eran más eficaces que las sulfonilureas o los iDPP-4 en la reducción de HbA1c, IMC y PAS; así como en la reducción de los riesgos de ingreso hospitalario por insuficiencia cardiaca y de progresión de la enfermedad renal^268^.

Si bien no ha habido un gran estudio diseñado para evaluar los efectos de la metformina sobre la morbimortalidad CV, si se han publicado numerosos metaanálisis mostrando su beneficio en prevención vascular^269^, ^270^, ^271^. En un metaanálisis sobre 40 estudios incluyendo 1.066.408 pacientes en prevención secundaria CV, metformina redujo un 33% la mortalidad por cualquier causa, un 19% la mortalidad CV y un 17% la incidencia de nuevos eventos CV^270^. No existen evidencias del beneficio CV de las sulfonilureas. Un metaanálisis sobre 82 ensayos controlados aleatorios mostró un mayor riesgo de mortalidad por todas las causas y de mortalidad cardiovascular para las SU en comparación con todos los demás tratamientos combinados CV^271^. Sin embargo, existen limitaciones metodológicas, y en los ensayos clínicos (Action in Diabetes and Vascular disease; preterAx and diamicroN-MR Controlled Evaluation [ADVANCE], Cardiovascular Outcome Trial of Linagliptin vs Glimepiride in Type 2 Diabetes [CAROLINA], Thiazolidinediones or Sulfonylureas and Cardiovascular Accidents Intervention Trial [TOSCA.IT], GRADE) se ha mostrado la seguridad cardiovascular de las nuevas SU frente a otros tratamientos. Por ello, no se recomienda su uso salvo para pacientes que ya las toman y no presentan contraindicaciones o riesgo de hipoglucemias; o en caso de no existir otra alternativa. En España actualmente la proporción de pacientes con SU es menor del 10%.

Los estudios en monoterapia frente a placebo, con inhibidores de la dipeptidil peptidasa 4 (iDPP4), Trial Evaluating Cardiovascular Outcomes with Sitagliptin (TECOS) (sitagliptina)^272^, Examination of Cardiovascular Outcomes with Alogliptin vs Standard of Care (EXAMINE) (alogliptina)^273^ y Saxagliptina assessment of vascular outcomes recorded in patients with diabetes (SAVOR-TIMI) (saxagliptina)^274^, Cardiovascular and Renal Microvascular Outcome Study With Linagliptin (CARMELINA) (linagliptina)^275^ muestran una no inferioridad en seguridad cardiovascular, si bien en el SAVOR-TIMI (saxagliptina) se observó un aumento de la hospitalización por insuficiencia cardiaca^274^.

En relación a los iSGLT2, el estudio Empagliflozin Cardiovascular Outcome Event Trial in Type 2 Diabetes Mellitus Patients–Removing Excess Glucose (EMPA-REG) (empagliflozina)^276^ en pacientes en prevención secundaria CV mostró una reducción significativa de la morbimortalidad cardiovascular (HR 0,86; IC 95% 0,74-0,99) al igual que el estudio Canagliflozin and Cardiovascular and Renal Events in Type 2 Diabetes (CANVAS) (canagliflozina)^277^ (HR 0,86; 0,75-0,97), si bien este con un 35% de pacientes en prevención primaria CV. En el estudio Dapagliflozin Effect on CardiovascuLAR Events (DECLARE)^278^, dapagliflozina no alcanzó una reducción significativa del objetivo cardiovascular principal (8,8% vs. 9,4%; HR 0,93; 0,84-1,03; p = 0,17), aunque sí alcanzó el criterio de no inferioridad frente a placebo. Igualmente, ertugliflozina^279^ alcanzó el resultado de no inferioridad en prevención vascular, sin reducción de eventos CV. Empagliflozina, además demostró un beneficio de reducción de muerte CV (HR 0,62; 0,49-0,77) y muerte por cualquier causa (HR 0,68; 0,57-0,82)^276^. Todos los iSGLT2 (empagliflozina, canagliflozina, dapagliflozina, ertugliflozina) redujeron de forma significativa, el riesgo de ingreso hospitalario por insuficiencia cardiaca (HR respectivos 0,65; 0,67; 0,73; 0,70). Empagliflozina, canagliflozina y dapagliflozina redujeron de forma significativa, el riesgo de progresión de la enfermedad renal^280^, ^281^, ^282^.

En relación a los arGLP1^283^, ^284^, ^285^, los estudios Liraglutide Effect and Action in Diabetes: Evaluation of Cardiovascular Outcome Results (LEADER) (liraglutida), Trial to Evaluate Cardiovascular and Other Long-term Outcomes with Semaglutide in Subjects with Type 2 Diabetes (SUSTAIN) (semaglutida) y Researching Cardiovascular Events With a Weekly Incretin in Diabetes (REWIND) (dulaglutida) mostraron reducción significativa del MACE de incidencia de morbimortalidad CV entre 12% (dulaglutida) y 26% (semaglutida). Liraglutida, observó una reducción del 22% de la muerte vascular (HR 0,78; IC 95% 0,66-0,93) y del 15% de la mortalidad de cualquier causa (HR 0,85; IC 95% 0,74-0,97). Aunque la mayoría de los arGLP1 son inyectables, se comercializa una formulación oral de semaglutida con efectos similares. El estudio Peptide InnOvatioN for Early DiabEtes TReatment (PIONEER) 6^286^ con semaglutida oral observó una reducción del 51% de la muerte cardiovascular (HR 0,49; IC 95% 0,27- 0,92; p = 0,03) y del 49% de la mortalidad de cualquier causa (HR 0,51; IC 95% 0,31-0,84; p = 0,008).

La tirzepatida es un nuevo agonista dual de los receptores del péptido insulinotrópico dependiente de la glucosa (GIP) y del arGLP1. En un metaanálisis comparando tirzepatida con arGLP1, insulina o placebo^287^, tirzepatida fue más eficaz en la reducción del peso corporal; frente a los arGLP1. La incidencia de hipoglucemia con tirzepatida fue similar a la del placebo e inferior a la de la insulina basal. Se observó una superioridad dosis-dependiente en la reducción de la HbA1c con las tres dosis de tirzepatida frente a todos los comparadores, con diferencias medias de -1,62% a -2,06% frente a placebo, -0,29% a -0,92% vs. arGLP1, y -0,70% a -1,09% frente a insulina basal^287^. En otro metaanálisis^288^ se incluyeron 13 ECA (9 y 4 sobre arGLP1 y tirzepatida, respectivamente) con 65878 pacientes con DM2. En comparación con placebo, los arGLP1 o los arGIP/GLP-1 redujeron los MACE (OR: 0,87; IC 95% 0,81-0,94; p < 0,01), la mortalidad por todas las causas (OR: 0,88; IC 95% 0,82-0,96; p < 0,01); y la mortalidad vascular (OR: 0,88; IC 95% 0,80-0,96; p < 0,01), sin diferencias entre los arGLP1 vs. arGIP/GLP-1. Además, los arGLP1 redujeron las probabilidades de ictus (OR: 0,84; IC 95% 0,76-0,93; p < 0,01) y de ictus no mortal (OR: 0,85; IC 95% 0,76-0,94; p < 0,01), mientras que no se descubrió ninguna asociación entre el ictus mortal y los arGLP1 (OR: 0,80; IC 95% 0,61-1,05; p = 0,105). Actualmente están realizándose un ensayo clínico específico con terzepatida sobre seguridad cardiovascular y prevención de eventos.

Otro metaanálisis^289^ revisando ocho estudios con iSGLT2 o arGLP1 incluyendo 77.242 personas con diabetes, se observó una reducción en la progresión de la enfermedad renal tanto con iSGLT2 (HR: 0,62; IC 95% 0,58-0,67; p < 0,001) como con arGLP1 (HR: 0,82; IC 95% 0,75-0,89; p < 0,001) pero solo los iSGLT2 redujeron el riesgo de empeoramiento del FG, enfermedad renal terminal o muerte de causa renal (HR: 0,55; IC 95% 0,48-0,64; p < 0,001). Los iSGLT2 redujeron la hospitalización por insuficiencia cardiaca un 31% (HR; 0,69; IC 95% 0,61-0,79; p < 0,001), pero no así los arGLP1-RA (HR 0,93; IC 95% 0,83-1,04; p = 0,20). Otro metaanálisis^290^ revisando 27 estudios incluyendo 56.004 pacientes, incluyendo asimismo los estudios Evaluation of Lixisenatide in Acute Coronary Syndrome (ELIXA), LEADER, SUSTAIN-6, Exenatide Study of Cardiovascular Event Lowering (EXSCEL), Effect of Albiglutide, When Added to Standard Blood Glucose Lowering Therapies, on Major Cardiovascular Events in Subjects With Type 2 Diabetes Mellitus (HARMONY), REWIND y PIONEER 6, concluye que los arGLP1 tiene beneficios demostrados en la prevención de indicadores de enfermedad renal en pacientes con DM2 (HR: 0,88 [IC 95% 0,81-0,96; p = 0·003]); y una reducción del 17% (0,83, 0,78-0,89; p < 0·0001) del objetivo compuesto de prevención de daño renal, fundamentalmente debido a la reducción de la excreción urinaria de albumina. No hubo incremento del riesgo de hipoglucemias graves, pancreatitis o cáncer de páncreas. En otro metaanálisis los arGLP-1 redujeron el riesgo de muerte por causas vasculares (RR: 0,90; IC 95% 0,83-0,97; p = 0,004) y de accidente cerebrovascular mortal o no mortal (RR: 0,85; IC 95% 0,77-0,94; p = 0,001) en comparación con los controles de placebo^291^. Otro metaanálisis comparando el uso de iDPP4 vs. arGLP1 o iSGLT2, concluye que los inhibidores de iSGLT2 y los arGLP1 son superiores a los iDPP-4 en la reducción del riesgo de la mayoría de los resultados cardio-renales^292^.

La insulina es un tratamiento muy eficaz para conseguir el control glucémico, pero asumiendo un mayor riesgo de hipoglucemias y de incremento de peso; y la necesidad de una educación terapéutica y del uso de tiras reactivas para determinación de glucemia capilar, o dispositivos de monitorización continua de glucosa (MCG). No tiene contraindicaciones para su uso en cualquier circunstancia, pero rquiere ajuste de dosis en pacientes con ERC o riesgo de hipoglucemia grave. Los análogos basales de insulina de acción más prolongada (Glargina U300 o Degludec), ofrecen menos riesgo de hipoglucemia nocturna^293^. En la mayoría de los pacientes, y especialmente en aquellos con obesidad, debería ser una opción terapéutica solo tras tratamiento previo con arGLP1 y/o iSGLT2^294^.

En los individuos en los que se intensifica el tratamiento con insulina, se ha demostrado que el tratamiento combinado con un arGLP1 o un AR dual de GIP y GLP-1 tiene una mayor eficacia, y durabilidad del efecto del tratamiento glucémico; así como beneficios en cuanto al peso y la hipoglucemia, que la intensificación del tratamiento solo con insulina^295^, ^296^.

Un metaanálisis sobre el RV asociado con el tratamiento de insulina en pacientes con DM2 (26 estudios, 24348 pacientes) concluye que el tratamiento con insulina basal no incrementa el riesgo de eventos vasculares o muerte por cualquier causa, mortalidad vascular, IAM o ictus. Sí se observó un incremento del riesgo de hipoglucemia grave (RR 2,98; IC 95% 2,47-3,61)^297^. Respecto al tipo de insulina, aunque los efectos sobre la HbA1c fueron comparables, el tratamiento con insulina glargina e insulina detemir hizo que menos participantes experimentaran hipoglucemia en comparación con la insulina NPH^297^, ^298^.

#### Intervención intensiva sobre la glucemia

En relación con los posibles beneficios vasculares tras un control glucémico intensivo, distintos metaanálisis observaron que el control intensivo de la glucemia (HbA1c < 7%) redujo el riesgo de eventos vasculares en un 9% (HR: 0,91; IC 95%, 0,84-0,99), fundamentalmente gracias a la reducción del 15% en el riesgo de IAM; y no se observó disminución de la mortalidad total ni de la vascular^299^, ^300^. Una cohorte española de 5.016 pacientes con DM2 mayores de 70 años observó que los pacientes con HbA1c < 7% presentaron menor incidencia de eventos vasculares (14%) que los que tenían HbA1c 7-8% (19%) o mayor de 8% (21%)^301^. Otro metaanálisis en pacientes mayores de 60 años o con fragilidad observó que el control intensivo se asoció a reducciones de las complicaciones microvasculares (0,73, IC 95% 0,68-0,79) y macro vasculares (0,84, IC 95% 0,79-0,89), pero el riesgo de hipoglucemia grave aumentó (2,45, IC 95% 2,22-2,72)^220^, y no se observaron diferencias en mortalidad. Los estudios más recientes con menor uso de antidiabéticos con riesgo de hipoglucemia producen mejores resultados preventivos vasculares con el control intensivo^302^.

En un estudio de cohortes retrospectivas^303^, con inclusión de 34.737 pacientes, los pacientes que tenían un HbA1c inferior a 6,5% en el primer año tras el diagnóstico de DM2, presentaron a los 13 de años de seguimiento, un menor riesgo de eventos microvasculares y macrovasculares; y los pacientes con HbA1c ≥ 7% presentaron un mayor riesgo de mortalidad: un aumento del 29% en pacientes con una HbA1c entre 7-8% y del 32% si la HbA1c era ≥ 9. En el seguimiento a 10 años después de finalizar el estudio UKPDS, a pesar de una pérdida temprana de las diferencias glucémicas entre los grupos con tratamiento intensivo o convencional al finalizar el estudio, se observó una reducción del riesgo microvascular y del riesgo de IAM y muerte por cualquier causa a los 10 años de seguimiento posterior al ensayo^304^. Estos resultados se han mantenido en un nuevo estudio con un seguimiento de 24 años de estos pacientes, confirmando la importancia de un control estricto en el momento del diagnóstico y sus efectos beneficiosos de por vida^232^.

### Estrategias terapéuticas

Las evidencias actuales en prevención cardio-renal, reducción de peso, mejora del RV y ausencia de hipoglucemias, posicionan a los iSGLT2 y arGLP1 en primera línea de tratamiento junto con la metformina. En pacientes en prevención secundaria vascular deberán priorizarse los iSGLT2 (empagliflozina, dapagliflozina, canagliglozina) y los arGLP1 con beneficios comprobados sobre los MACE (liraglutida, semaglutida subcutáneo y dulaglutida) frente a metformina, así como en pacientes con ERC (empagliflozina, canagliglozina, dapagliflozina). En pacientes con insuficiencia cardiaca los iSGLT2 (empagliflozina, canagliglozina, dapagliflozina, ertugliflozina) serán los fármacos a priorizar. En los pacientes con obesidad se deben priorizar los fármacos más eficaces en la reducción de peso (tirzepatida, semaglutida). En estos escenarios, los ArGLP1 e iSGLT2 deben utilizarse asociados a la modificación del estilo de vida e independientemente del control glucémico y del uso de metformina u otros fármacos hipoglucemiantes.

La creciente apreciación de la interrelación fisiopatológica de factores de riesgo metabólicos como la obesidad y la diabetes, la ERC y las EV ha llevado a la conceptualización del síndrome metabólico cardiovascular-renal^305^. La caracterización de este síndrome no hace más que reforzar la necesidad de un abordaje integral de los pacientes que generalmente presenta multimorbilidad y donde el papel del médico de familia es insustituible.

La guía del *Primary Care Diabetes Europe* (PCDE)^306^ recomienda inicio de tratamiento combinado para evitar la inercia y mejorar el grado de control siguiendo los resultados del estudio Vildagliptin Efficacy in combination with metfoRmIn For earlY treatment of type 2 diabetes (VERIFY)^307^; y la guía de la Sociedad Española de Medicina de Familia y Comunitaria (semFYC)^308^ recomienda no esperar más de tres meses, tras el inicio o modificación terapéutica, para realizar una analítica e intensificar el tratamiento si no se alcanza el control y la adherencia es adecuada. Esta recomendación (semFYC) se adecua mejor a las indicaciones reconocidas en ficha técnica que no recogen la terapia combinada de inicio.

### Objetivos y grado de control

En líneas generales, se debe observar un objetivo de control menos estricto en pacientes ancianos o con comorbilidades asociadas o con pobre calidad de vida, entendiendo por un control estándar una HbA1c del 7-8%.

A pesar de los avances terapéuticos, el grado de control no presenta grandes modificaciones. Así, en un estudio poblacional sobre registros en historia clínica electrónica en práctica clínica en Cataluña^309^, en el periodo 2007-2018 analizando, respectivamente, 299.855 y 394.266 personas con diabetes, la proporción de pacientes con HbA1c menor de 7% fue de 54,9% en 2007 y de 55,9% en 2018. Se observó una mejoría en el control de la PA (PA< 140/90 mmHg) desde el 55% en 2007 al 71,8% en 2018; así como en el control de LDL colesterol (< 100 mg/dL) desde el 33,4% al 48,4%. El control integral de estos tres factores de riesgo mejoró de 12,5% a 20,1% en la población total con DM2 y desde el 24,5% al 32,2% en las personas con DM2 en prevención secundaria. Otro estudio en Reino Unido^310^ realizado en el periodo 2012-2016, en 164 centros de AP, mostraba un 46,7% de pacientes con HbA1c menor de 7%.

Entre las causas^311^ destacan la falta de adherencia terapéutica por parte del paciente y la inercia clínica^312^ o falta de intensificación del tratamiento por parte del profesional sanitario. Diversos criterios se emplean para valorar la adherencia, como son la persistencia en la prescripción de la medicación, o el porcentaje entre las dosis de fármaco dispensadas y las dosis necesarias en un periodo de tiempo (*Medication Possesion Ratio* [MPR])^313^. Una revisión sistemática^314^ mostró una MPR de 75,3% (IC 95% 68,8%-81,7%) con una proporción de pacientes con buena adherencia de 67,9% (IC 95% 59,6%-76,3%).

Un estudio en Alemania analizando 1201 consultas de AP, observó un mayor porcentaje de discontinuidad del tratamiento en los pacientes tratados con sulfonilureas que en los tratados con iDPP4 (49 vs. 39%; HR 0,74 IC 95% 0-71-0,76) con una incidencia de eventos vasculares un 26% menor en los pacientes tratados con iDPP4^315^. Otro metaanálisis, muestra como en personas con DM2, una buena adherencia se asocia con una reducción del riesgo de hospitalización; así como de la mortalidad por todas las causas^316^.

Debe destacarse que la adherencia a las guías de práctica clínica en AP es baja^317^. Entre las causas de falta de adherencia destacan la falta de conocimiento sobre la enfermedad, sobre el tratamiento o sobre las recomendaciones de autocuidado; el carecer de apoyo social, o la comorbilidad como la depresión o el deterioro cognitivo. Los fármacos con más efectos adversos pueden comprometer la adherencia terapéutica, así como los altos costes de los mismos; y la buena relación profesional sanitario-paciente y una adecuada educación sanitaria favorecen una mejor adherencia^318^. En un estudio cualitativo^319^ para identificar medidas de mejora de la adherencia, desde el ámbito de la AP, la soluciones propuestas fueron simplificar los tratamientos, medir la adherencia, revisar la medicación de forma periódica, uso de terapia combinada a dosis fijas, y potenciar la educación terapéutica y la entrevista motivacional. En otro estudio portugués, las estrategias de facilitación más útiles incluían la reducción de las dosis diarias, la revisión de las opciones terapéuticas y las intervenciones de motivación. Sin embargo, menos de la mitad de los médicos reconocieron preguntar a sus pacientes si se tomaban la medicación^320^.

La inercia terapéutica o falta de intensificación del tratamiento a pesar de no alcanzar los objetivos establecidos es otra causa importante de falta de control glucémico. Una revisión sistemática revisando 53 artículos^312^, concluyó que el tiempo medio para la intensificación terapéutica fue de un año (rango 0,3-7,2), hubo mayor inercia cuando el número de fármacos era mayor, y menor cuando los niveles de HbA1c eran más altos. Un estudio español^321^ observó que no se realizaba intensificación en uno de cada cinco pacientes con mal control y que el 26% de los pacientes con HbA1c mayor de 7% permanecían sin intensificar su tratamiento después de cuatro años de seguimiento.

**Figura 6:**
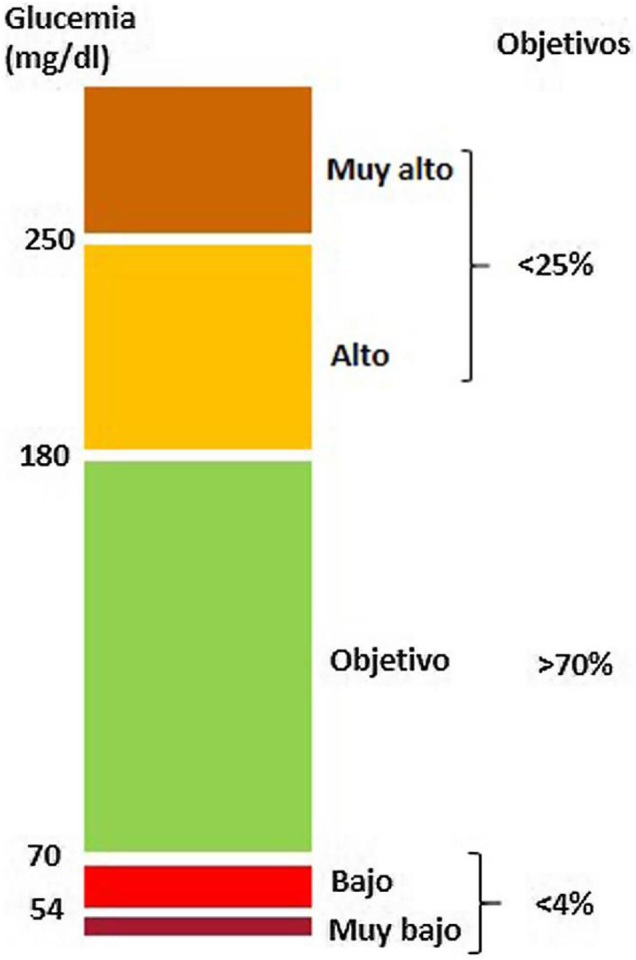
Criterios de buen control para dispositivos de monitorización continua de glucosa. Adaptado de ^214^.

La MCG puede ser una solución interesante en pacientes con DM2 tratados con múltiples dosis de insulina para mejorar el grado de control glucémico y reducir las hipoglucemias. Un reciente metaanálisis muestra como la MCG se asocia a niveles de glucosa en sangre más bajos (HbA1c -2,69, IC 95% -4,25, -1,14, p < 0,001) que el método tradicional de autotest de glucosa en sangre capilar y la incidencia de hipoglucemia grave se reduce significativamente (RR = 0,52, IC 95%: 0,35-0,77, y p = 0,001^322^. La MCG valora parámetros diferentes a los empleados en práctica clínica habitual (glucemia, HbA1c), siendo el tiempo en rango el más empleado y se considera buen control un valor > = 70%^323^; valor que se aproxima al objetivo tradicional de Hba1c < 7%. Aunque se puede ser más específico describiendo además el tiempo con valores glucémicos muy altos o muy bajos (fig. 6). La MCG está financiada por las comunidades autónomas en España para pacientes con DM2 y múltiples dosis de insulina y la prescripción se recomienda que se gestione donde el paciente recibe la atención (AP y hospitalaria), por lo que es recomendable la formación sobre estos nuevos dispositivos tanto en medicina de familia como en enfermería.

En la tabla 14 se recoge, de forma resumida, la calidad de la evidencia y la fuerza de la recomendación sobre el manejo de la DM.

**Tabla 14:** Recomendaciones en diabetes tipo 2

*Prueba diagnóstica y control glucémico*	Calidad de la evidencia	Fuerza de la recomendación
Realizar cribado poblacional de DM2	Alta	Fuerte en contra
La prueba de cribado recomendada es la glucemia basal	Moderada	Fuerte a favor
La HbA1c es preferible para monitorizar el control de la glucemia en la persona con DM2	Moderada	Fuerte a favor
Se recomienda como buen control una HbA1c < 7% en la mayoría de los pacientes siempre que sea posible alcanzarlo sin causar hipoglucemias	Alta	Fuerte a favor
Deben calcularse objetivos individualizados, más estrictos en pacientes jóvenes sin complicaciones (HbA1c < 6,5%) y menos estrictos en pacientes mayores con comorbilidades importantes (8%). Se recomienda el uso de la app para smartphones (*A1c Calculator Application*) para el cálculo de objetivos individualizados	Moderada	Débil a favor

*Tratamiento farmacológico*	Calidad de la evidencia	Fuerza de la recomendación
Los 3 grupos farmacológicos de uso prioritario en el tratamiento de la DM2 son la metformina, los iSGLT2 y los arGLP1, solos o en combinación.	Alta	Fuerte a favor
En pacientes con insuficiencia cardiaca y diabetes, los ISGLT2 deben formar parte del tratamiento	Alta	Fuerte a favor
En pacientes con diabetes y enfermedad renal crónica, los ISGLT2 y/o arGLP1 deben formar parte del tratamiento	Alta	Fuerte a favor
En pacientes con diabetes y prevención secundaria CVx, los ISGLT2 y/o arGLP1 deben formar parte del tratamiento	Alta	Fuerte a favor
La glibenclamida no debe emplearse por el mayor riesgo de hipoglucemias respecto a otras opciones	Alta	Fuerte a favor
Debe evitarse la asociación de iDPP4 y arGLp1, por compartir el mismo mecanismo de acción.	Moderada	Débil a favor
Se recomienda alcanzar un grado de control óptimo (HbA1c < 6,5%) en el momento de diagnóstico de DM y meses posteriores por el «efecto legado».	Alta	Fuerte a favor
Se recomienda emplear insulinas (glargina u 300, degludec) con menor riesgo de hipoglucemia nocturna.	Alta	Fuerte a favor

*Recomendaciones con menor evidencia*		
La periodicidad mínima de la determinación de la glucemia en población de riesgo es cada cuatro años	Baja	Débil a favor
El test FINDRISC permite evitar la glucemia en el cribado de la prediabetes y diabetes partir de un punto de corte (≥ 15 puntos)	Baja	Débil a favor
Deben usarse insulinas con bajo riesgo de hipoglucemias.	Baja	Débil a favor

*No existe suficiente evidencia para su recomendación*		
Control intensivo de glucemia a todos los pacientes	Baja	Débil a favor

arGLP1: agonistas del péptido similar al glucagón tipo 1 (*Glucagón-like peptid-1 receptor agonists)*; iSGLT2: Inhibidores del cotransportador de sodio-glucosa tipo 2 (*Sodium-glucose cotransporter-2 inhibitors.*

Actualmente, ya se dispone de una *app* para *smartphones* (A1c: *calculator application*), que es gratuita^408^.

Elaboración propia.

## Enfermedad renal crónica

### Importancia epidemiológica y clínica

La ERC afecta al 11% de la población mundial y se prevé aumento de la prevalencia a nivel mundial, debido al envejecimiento de la población y a la exposición a factores de riesgo de ERC como la DM2, la obesidad o la HTA^324^. La importante morbilidad, mortalidad y costes sanitarios asociados a esta enfermedad hacen que la ERC sea un problema de salud pública mundial. Se están destinando grandes esfuerzos a nivel internacional para aumentar el conocimiento y la sensibilización sobre el impacto de la ERC; y realizar políticas preventivas de la enfermedad. Europa y EE. UU. han declarado la década 2021-2030 como la década del riñón^325^.

#### Prevalencia en España de la enfermedad renal crónica

En el mundo, hay en torno a 850 millones de personas con ERC^326^ y en España, casi 7 millones^327^. La prevalencia de la ERC en sus fases más avanzadas en España ha crecido casi un 30% en los últimos 10 años, pasando de 1.054 pacientes por millón de población en 2011, a 1,363 en 2020^327^. En población general la prevalencia de ERC en España es de un 15%, mientras que en personas mayores de 65 años, la prevalencia es del 37%. A pesar de ello, se calcula que tan solo un 4,9% ha recibido un diagnóstico sobre su enfermedad^327^.

### Cribado y pruebas diagnósticas

La ERC es una enfermedad progresiva, asintomática en las fases iniciales. El diagnóstico de la ERC suele producirse en estadios más avanzados, en los que el daño renal del paciente es más severo y suele ir acompañado de otras complicaciones. Un diagnóstico tardío de la ERC está directamente relacionado con un peor pronóstico, debido al mayor avance del daño renal, al incremento de la mortalidad vascular, comienzo tardío del tratamiento y mayor riesgo de complicaciones derivadas de la administración de algunos fármacos^328^.

Es necesario identificar a los individuos con riesgo de ERC y a los que se encuentran en las primeras fases de la ERC, para permitir el inicio precoz del tratamiento, evitando así la progresión a insuficiencia renal y reduciendo el riesgo de ECV, hospitalización y mortalidad.

La ERC es definida por el Grupo de Trabajo KDIGO sobre ERC basándose en los siguientes criterios presentes durante más de tres meses: tasa de filtración glomerular estimada (TFGe) < 60 mL/min/1, 73 m^2^ o marcadores de daño renal (una o más anomalías estructurales o funcionales, albuminuria (relación albúmina/creatinina en orina [UACR] ≥ 30 mg/g), anomalías del sedimento urinario, hematuria persistente, anomalías electrolíticas y de otro tipo debidas a trastornos tubulares, anomalías detectadas por histología, anomalías estructurales detectadas por imagen o antecedentes de trasplante renal)^146^. Siguiendo esta definición, el hallazgo incidental de un FG < 60 mL/min/1,73 m^2^ y/o un índice albúmina/ creatinina ≥ 30 mg/g, obliga a repetir la determinación en un plazo de al menos tres meses para confirmar el diagnóstico^138^. Seguir esta recomendación es especialmente importante en la población más joven y de mediana edad (población en edad laboral), en la que una segunda determinación del FG ha demostrado reducir la prevalencia de ERC a la mitad^329^.

**Figura 7:**
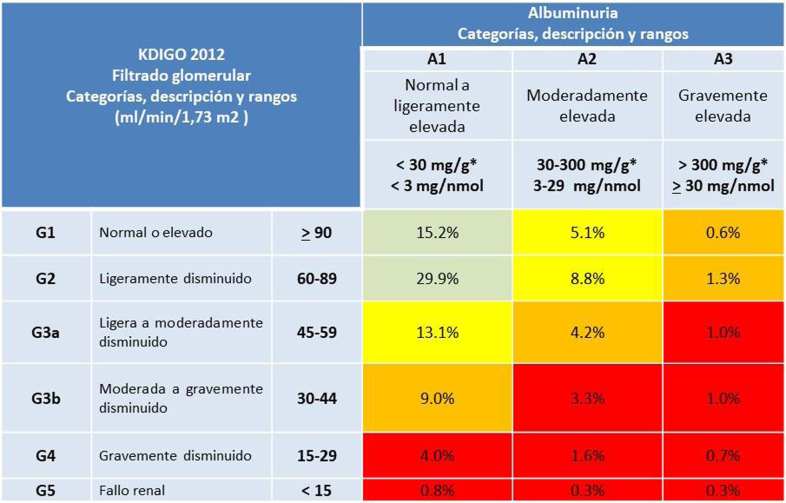
Clasificación de la enfermedad renal crónica según filtrado glomerular y albuminuria. Prevalencias en España y riesgo. Tomado de 313, 315, 316. La albuminuria se expresa como índice albúmina/creatinina. Los porcentajes expresan la proporción de pacientes esperados en España en cada casilla según el estudio. Áreas en color verde: situaciones sin enfermedad renal crónica ni riesgo de referencia; áreas en color amarillo: riesgo moderadamente aumentado; áreas en color naranja: riesgo alto; área en color rojo: riesgo muy alto. KDIGO: *Kidney Disease: Improving Global Outcomes*.

La clasificación de la ERC contempla una división de seis categorías de riesgo en función del FG (G1-G5), que se complementan con tres categorías de riesgo según el índice albúmina creatinina (CAC) (A1-A3) (fig. 7).

La disminución del FG, al igual que el aumento del CAC, se asocian a un aumento de eventos adversos (mortalidad global, mortalidad vascular, fracaso renal tratado con diálisis o trasplante, fracaso renal agudo y progresión de la enfermedad renal). La coexistencia de una disminución del FG y un CAC aumentado multiplica el riesgo.

En España el 80% de los sujetos con ERC (80,6%; 12,2% del total de la muestra) se corresponden con situaciones de riesgo moderado de complicaciones cardiorrenales, lo que establece un importante margen para la prevención tanto vascular como renal, y sitúan al médico de familia en una posición protagonista^330^.

### Tratamiento

#### Medidas no farmacológicas

En el tratamiento de la ERC tenemos que recomendar cambios en el estilo de vida para controlar el peso, aumentar el ejercicio, dejar de fumar, ajustar la dieta y asegurar más de seis horas de sueño por noche. Debemos también tratar las comorbilidades, incluyendo la optimización del control de la HbA1c, la hipertensión y las alteraciones lipídicas, y el control de la insuficiencia cardiaca y la apnea del sueño^146^.

#### Tratamiento farmacológico

##### Inhibidores del *Renin-angiotensin-aldosterone system* (RAAS)

Se recomienda tratar con IECA o ARA-II a dosis máximas toleradas como de primera línea de tratamiento en individuos con DM2 con HTA y albuminuria, ya que reducen la PA y disminuyen el riesgo de progresión de la ERC, basándose en los resultados de ensayos controlados aleatorizados^331^. Losartán en el estudio Reduction of End Points in Non-Insulin-Dependent Diabetes With the Angiotensin II Antagonist Losartan (RENAAL), redujo el riesgo de la variable de valoración renal compuesta (duplicación de la creatinina sérica, ERC estadio terminal o muerte) en un 16% en personas con DM2 y nefropatía^332^. Con irbesartán se observó una reducción del 20% del riesgo del criterio de valoración renal compuesto en individuos con hipertensión y nefropatía debida a la DM2^333^.

##### Inhibidores SGLT-2 (iSGLT2)

Se recomienda un iSGLT2 con beneficio renal o vascular demostrado como segundo pilar del tratamiento de la ERC en individuos con DM2. En el estudio Empagliflozin in Patients with Chronic Kidney Disease (EMPA-KIDNEY), empagliflozina redujo la progresión de la ERC o la muerte por causas vasculares en un 36% y la hospitalización por insuficiencia cardiaca o muerte vascular en un 16% en comparación con placebo en pacientes con diabetes^280^. Dapagliflozina ralentizó la progresión de la ERC (compuesto de un descenso sostenido del FGe de al menos el 50%), ERC estadio terminal o muerte por causas renales o vasculares en un 39% en participantes con ERC, con o sin DM2, en Dapagliflozin in Patients with Chronic Kidney Disease (DAPA-CKD). El compuesto de muerte por causas vasculares u hospitalización por insuficiencia cardiaca se redujo en un 29%, y la muerte por todas las causas se redujo en un 31% con dapagliflozina en comparación con placebo^282^. En el ensayo clínico Canagliflozin and Renal Events in Diabetes with Established Nephropathy Clinical Evaluation (CREDENCE), canagliflozina mejoró los resultados renales (enfermedad renal terminal [diálisis, trasplante o un FGe sostenido < 15 mL/min/1,73 m^2^], una duplicación del nivel de creatinina sérica o la muerte por causas renales o vasculares) en un 30% y los resultados vasculares (muerte vascular, IAM o ictus) en un 20% en los participantes con DM2 y ERC albuminúrica que fueron tratados con bloqueo del SRAA máximo tolerado^281^.

##### ARGLP1

Una metaanálisis de ocho ensayos clínicos con un total de 60.080 personas con DM2 demostró que el tratamiento con un ARGLP1 reducía el criterio de valoración renal compuesto (macroalbuminuria, duplicación de la creatinina sérica o disminución ≥ 40% de la TFGe, tratamiento renal sustitutivo o muerte por enfermedad renal) en un 21% en comparación con placebo^290^. Posteriormente, un ensayo específico de resultados renales, el Evaluate Renal Function with Semaglutide Once Weekly (FLOW), demostró que la semaglutida ralentizaba el descenso del FGe, reducía la progresión de la ERC (combinación de la aparición de insuficiencia renal, al menos una reducción del 50% del FGe con respecto al valor basal o la muerte por causas renales o vasculares) en un 24% y los acontecimientos vasculares graves en un 18% en comparación con placebo en personas con ERC y DM2^334^.

##### Antagonistas del receptor mineralcorticoide no esteroideos

Finerenona es el único antagonista del receptor mineral (ARM) no esteroideo aprobado para ralentizar la progresión de la ERC en individuos con ERC diabética; y enlentece la progresión de la ERC al actuar sobre la inflamación y la fibrosis^335^, por sus efectos hemodinámicos^336^. En el ensayo clínico Efficacy and Safety of Finerenone in Subjects With Type 2 Diabetes Mellitus and Diabetic Kidney Disease (FIDELIO-DKD), la finerenona redujo la aparición de la variable principal de valoración renal compuesta (insuficiencia renal, disminución sostenida de ≥40% de la TFGe con respecto al valor basal o muerte por causas renales) en un 18% en comparación con placebo en participantes con diabetes y ERC^337^. En el estudio Efficacy and Safety of Finerenone in Subjects With Type 2 Diabetes Mellitus and the Clinical Diagnosis of Diabetic Kidney Disease (FIGARO-DKD), la finerenona redujo el compuesto de muerte por causas vasculares, IM no mortal, ictus no mortal u hospitalización por insuficiencia cardiaca en un 13% y la incidencia de insuficiencia cardiaca de nueva aparición en un 32% en comparación con placebo en participantes con diabetes y ERC^19^. En un análisis conjunto (FIDELITY) de los datos de FIDELIO-DKD y FIGARO-DKD, la finerenona redujo los resultados vasculares en un 19% y el resultado renal compuesto (insuficiencia renal, disminución sostenida > 57% del FGe o muerte relacionada con el riñón) en un 23% en comparación con placebo en todo el espectro de gravedad de la ERC^336^. No se recomienda su uso con FG < 25 mL/min/1,73 m^2^ ni con niveles de potasio > 5 mEq/L; y es preciso ajuste de dosis en función de estos parámetros^338^.

Por otro lado, en el estudio FIDELIO, el uso de finerenona en pacientes con ERC y DM2, dio lugar a una reducción del 18% (RR = 0,82; IC 95% 0,73-0,93; p = 0,001) del riesgo de progresión de la ERC y de eventos vasculares^337^. Entre los pacientes con DM2 y ERC de estadio 2 a 4 con albuminuria moderadamente elevada o ERC de estadio 1 o 2 con albuminuria gravemente elevada, el tratamiento con finerenona mejoró los resultados vasculares en comparación con el placebo^339^.

### Conclusiones

La ERC es muy prevalente en las consultas de Atención Primaria y probablemente a día de hoy, todavía, está infradiagnosticada.

Debemos tener un manejo adecuado del FG (CKD-EPI) y el IAC para su detección, control y seguimiento evolutivo.

El paciente con ERC es un paciente de alto o muy alto RV lo que implica un conocimiento de las peculiaridades del control de los FRV clásicos en esta población.

## Antiagregación

### Importancia epidemiológica y clínica del problema

Se ha documentado que la utilización de ácido acetilsalicílico (AAS) en prevención secundaria alcanza un grado de utilización razonablemente alto, alrededor del 94%^340^, en consonancia con la abundante evidencia existente. Sin embargo, en prevención primaria existen incertidumbres sobre su balance beneficio-riesgo y las recomendaciones de las guías de práctica clínica se han modificado según han ido apareciendo nuevas evidencias, lo que se traduce en mucha heterogeneidad en el uso de AAS en pacientes que no han presentado una EV, incluidos los pacientes de alto RV y los pacientes diabéticos. En el estudio European survey of cardiovascular disease prevention and diabetes (EUROASPIRE) III en prevención primaria, el tratamiento antiagregante se había prescrito en el 22% de los pacientes de alto riesgo sin EV y en el 28,2% de los diabéticos^341^. Los antiagregantes por vía oral actualmente disponibles en España son los siguientes: los inhibidores de la síntesis de tromboxano: AAS (el más utilizado) y el trifusal; inhibidores de la fosfodiesteresa: dipiridamol, y los inhibidores de la activación plaquetaria mediada por *adenosine di phosphate* (ADP) (inhibidores del receptor P2Y12) ticlopidina, clopidogrel, prasugrel, ticagrelor.

### Revisión de la evidencia

#### Prevención secundaria

El tratamiento antiagregante con AAS a una dosis de entre 75 y 150 mg/día en pacientes con EV previa (prevención secundaria) produce una reducción significativa de todos los episodios vasculares mayores y de la mortalidad vascular y total^342^. En pacientes con alergia o intolerancia al AAS, el clopidogrel es la alternativa de elección^343^. En el caso específico de la prevención de la recurrencia del ictus, se puede recomendar como primera línea de tratamiento tanto AAS 50-300 mg como clopidogrel 75 mg, trifusal 300 mg o la combinación AAS (25 mg)/dipiridamol (200 mg en liberación retardada), dos veces al día (en España solo está comercializado dipiridamol de liberación rápida)^344^. En un reciente metaanálisis ticagrelor fue ligeramente superior a clopidogrel o AAS en la reducción del riesgo de ictus en personas con antecedentes de EV^345^.

#### Prevención primaria

Se han publicado diferentes metaanálisis en prevención primaria de la EV. Baigent et al. mostraron una reducción relativa de un 12% de todos los EV (AAS 0,51% frente a control 0,57% por año; p = 0,0001), que se debió, en su mayoría, a una reducción relativa de un 23% para el IAM no fatal (AAS 0,18% vs. control 0,23% por año; p < 0,0001)^342^.

De Berardis et al. analizaron los resultados según el sexo, y observaron que el AAS reduce significativamente el riesgo de IAM en varones en un 43%, sin encontrar diferencias en mujeres; mientras que el AAS reducía el riesgo de ictus en mujeres en un 25% de una manera significativa, sin encontrar diferencias en varones^346^. No hay evidencia de que el AAS reduzca significativamente la mortalidad por EV ni la mortalidad total, aunque, en un metaanálisis, la reducción observada alcanzó el límite de la significación (RR: 0,94; IC 95%, 0,88-1,00; p = 0,05)^347^.

Tres ensayos clínicos (ASPirin in Reducing Events in the Elderly [ASPREE] en ancianos, A Study of Cardiovascular Events iN Diabetes [ASCEND] en personas con diabetes y Aspirin to Reduce Risk of Initial Vascular Events [ARRIVE] en personas con RV moderado) añaden nuevas evidencias en prevención primaria. ASPREE (19.114 personas mayores de 70 años seguidos 4,7 años) no observó reducción significativa de riesgo CV (RR 0,95, 0,83-1,08) y sí incremento significativo de hemorragias (1,38; 1,18-1,62)^348^. El estudio ASCEND (15.480 personas con diabetes seguidas una media de 7,4 años) observó una reducción significativa de EV (RR 0,88; IC 95% 0,79-0,97) así como un incremento significativo de sangrados (RR 1,29; IC 95% 1,09-1,52)^349^. El estudio ARRIVE (12.546 personas con riesgo CV moderado seguidas durante 60 meses) no observó reducción significativa de EV (HR 0,96; IC 95% 0,81-1,13) y sí un incremento significativo de hemorragias (HR 2,11; IC 95% 1,36-3,28)^350^. Un metaanálisis más reciente que incluye dichos estudios y que en conjunto analiza 164.225 personas asocia el uso de AAS en prevención primaria con una reducción significativa de EV (HR 0,89; IC 95% 0,84-0,95) con un NNT de 265, así con un incremento significativo de sangrado (HR 1,43; IC 95% 1,30-1,56), con un NNH de 210^351^.

#### Hemorragias

En el metaanálisis de Baigent et al., se observó un aumento significativo de riesgo de hemorragia mayor extracraneal con AAS (RR: 1,54; IC 95%, 1,30-1,82; p < 0,0001), en su mayoría no fatal^342^. Otro metaanálisis mostró aumento significativo del riesgo de ictus hemorrágico (RR: 1,36; IC 95%, 1,01-1,82; p = 0,04), hemorragia mayor (RR: 1,66; IC 95%, 1,41-1,95; p < 0,00001) y hemorragia gastrointestinal (RR: 1,37; IC 95%, 1,15- 1,62; p = 0,0003)^346^, mientras que otro mostró el mismo incremento de riesgo, tanto para el ictus hemorrágico como para la hemorragia mayor^352^. El metaanálisis de Seshasai et al. mostró un exceso de riesgo del 70% del total de las hemorragias y del 30% de las hemorragias no menores^353^. Varios metaanálisis sobre eficacia y seguridad de la AAS en prevención primaria subrayan el incremento significativo de hemorragias: sangrado principal (RR 1,47, IC 95% 1,31-1,65) y hemorragia intracraneal (RR 1,33, IC 95% 1,13-1,58)^354^. En el metaanálisis de Zheng et al. observa un incremento de sangrado principal del 43% (HR 1,43, 1,30- 1,56), de sangrado intracraneal del 34% (HR 1,34, 1,14-1,57) y de sangrado gastrointestinal del 56% (HR 1,56, 1,38-1,78)^351^.

#### Ácido acetilsalicílico y diabetes

Un metaanálisis, que incluye el ASCEND, observa en 34.227 participantes seguidos durante cinco años que AAS aporta una reducción significativa de episodios vasculares MACE (RR 0,89, 0,83-0,95) con un NNT de 95 para prevenir un episodio MACE en cinco años; hubo una reducción de ictus con dosis ≤ 100 mg/día (0,75, 0,59-0,95). No hubo resultados significativos en la reducción de la mortalidad por cualquier causa. No hubo significación estadística en el riesgo de sangrado (1,30, 0,92- 1,82), aunque algunas de las estimaciones fueron imprecisas^355^.

#### Ácido acetilsalicílico e insuficiencia renal

Un análisis de subgrupos post hoc del estudio *Hypertension Optimal Treatment* (HOT) mostró que el beneficio del AAS era mayor en pacientes con hipertensión y ERC, con un aumento progresivo del beneficio en la reducción de los eventos vasculares y la mortalidad total en los pacientes con un FG < 45 mL/min/1,73 m^2^ 356, 357.

Un metaanálisis sobre el uso de antiagregantes en pacientes con ERC analizó el efecto del AAS en el contexto de un síndrome coronario agudo y también en pacientes con enfermedad coronaria estable o pacientes de alto riesgo. Concretamente, en este último grupo se observó una reducción significativa del IAM fatal o no fatal, no así en el ictus o en la mortalidad vascular o por todas las causas, con un aumento de las hemorragias menores. Los resultados fueron inciertos por la baja calidad de los estudios incluidos^358^.

#### Pacientes tratados mediante revascularización coronaria

Respecto a la doble antiagregación tras una revascularización coronaria, la estrategia a seguir dependerá del contexto clínico, de la urgencia y del tipo de intervención (intervención coronaria percutánea [ICP] o cirugía de *bypass*), así como del tipo de *stent* implantado en su caso^359^, ^360^. El *stent* metálico, sin embargo, es el dispositivo de elección, independientemente de la duración prevista para la doble antiagregación.

*Pacientes coronarios estables tratados mediante una intervención coronaria percutánea electiva.* Se recomiendan seis meses de doble antiagregación con AAS y clopidogrel, independientemente del tipo de *stent* metálico implantado. El AAS se recomienda como tratamiento de por vida.

*Pacientes que han padecido un síndrome coronario agudo sin elevación del ST (SCASEST) tratados mediante una intervención coronaria percutánea*. Se recomienda doble antiagregación durante 1 año con AAS 75-100 mg más un inhibidor del receptor plaquetario P2Y12, siempre y cuando no exista una contraindicación formal (p.ej., exceso de riesgo de hemorragia). Se recomienda ticagrelor 90 mg, dos veces al día, en pacientes con un moderado o alto riesgo isquémico (como haber tenido elevación de troponinas), independientemente del tratamiento inicial. Se recomienda prasugrel 10 mg, 1 dosis diaria, en los pacientes con anatomía coronaria conocida tratados con ICP. Se recomienda clopidogrel 75 mg diarios en los pacientes que no pueden recibir ticagrelor o prasugrel, o que requieran adicionalmente anticoagulación oral (triple terapia).

*Pacientes que han padecido un infarto agudo de miocardio con elevación del ST (IAMCEST) tratados mediante una intervención coronaria* percutánea^361^, ^362^. Doble antiagregación durante un año con AAS 75-100 mg más un inhibidor del receptor plaquetario P2Y12, siempre y cuando no exista una contraindicación formal (p. ej., exceso de riego de hemorragia). Se puede utilizar cualquiera de estas opciones terapéuticas: clopidogrel, ticagrelor o prasugrel. Un metaanálisis que comparó los resultados de los pacientes que recibieron doble antiagregación durante < 12 meses, 12 meses exactos o más de 12 meses^363^ mostró que los pacientes que descontinuaron antes de los 12 meses después de una ICP con *stent* liberador de fármacos tenían significativamente menos riesgo de hemorragia sin un mayor riesgo de eventos isquémicos que los que alcanzaban 12 o más meses. Por contra, los pacientes que seguían un tratamiento con doble antiagregación más de 12 meses observaron una reducción de las complicaciones isquémicas, especialmente la trombosis del *stent* y la incidencia de nuevos infartos; sin embargo, aumentaban las complicaciones hemorrágicas con riesgo de muerte.

*Pacientes con revascularización miocárdica mediante cirugía (bypass coronario)*. Se recomienda el AAS a dosis bajas de forma indefinida, o el clopidogrel en caso de intolerancia al AAS, en los casos de cardiopatía isquémica estable. Por el contrario, en los pacientes con SCA, la doble antiagregación ha probado su eficacia en reducir el riesgo isquémico con independencia de la forma de revascularización. La doble antiagregación en los pacientes con EV crónica y estable, ya sea coronaria o de otra localización, no es más eficaz que el AAS solo en la reducción de nuevos episodios vasculares^364^.

*Utilización de los inhibidores de la bomba de protones*. Se recomienda la utilización de inhibidores de la bomba de protones a todos los pacientes con doble antiagregación.

#### Pacientes con diagnóstico de fibrilación auricular y enfermedad coronaria estable

*Pacientes con un CHA2DS2-VASc >* *2 y un riesgo bajo de hemorragia (HAS-BLED <* *2).* Implantación de *stent* no farmacoactivo: doble antiagregación y un anticoagulante oral (triple terapia), durante un mes, seguido de doble terapia con un anticoagulante oral y un antiagregante (AAS o clopidogrel) durante un año. Después un anticoagulante oral de por vida.

*Implantación de stent farmacoactivo*. Doble antiagregación y un anticoagulante oral (triple terapia) de tres a seis meses, dependiendo del tipo de *stent*, seguido de doble terapia con un anticoagulante oral y un antiagregante (AAS o clopidogrel) durante un año. Después un anticoagulante oral de por vida.

*Pacientes con un CHA2DS2-VASc >* *2 y un riesgo alto de hemorragia (HAS-BLED >* *3).* Se recomienda colocar un *stent* no farmacoactivo y triple terapia de 2-4 semanas, y después solo anticoagulante de por vida.

#### Pacientes con diagnóstico de fibrilación auricular y síndrome coronario agudo.

En los pacientes con un HAS-BLED ≤ 2 se debe considerar la triple terapia durante seis meses, seguida de un anticoagulante oral más AAS o clopidogrel hasta 12 meses.

En los pacientes con un HAS-BLED ≥ 3 se debe considerar la triple terapia durante un mes, seguida de AAS o clopidogrel y un anticoagulante oral.

En la tabla 15 se recoge, de forma resumida, la calidad de la evidencia y la fuerza de la recomendación sobre antiagregación.

**Tabla 15:** Recomendaciones sobre antiagregación

	Calidad de la evidencia	Fuerza de la recomendación
AAS en prevención secundaria. El tratamiento con AAS a dosis bajas se debe utilizar en todos los pacientes diagnosticados de enfermedad coronaria, ictus o accidente isquémico transitorio, o enfermedad arterial periférica sintomática, de forma indefinida.	Alta	Fuerte a favor
AAS en prevención primaria. No se recomienda el uso de AAS de forma sistemática enprevención primaria, incluidas las personas con diabetes. De forma individualizada, y valorando la preferencia del paciente, se podría valorar su utilización en ausencia declaras contraindicaciones	Baja	Débil a favor
Clopidogrel como alternativa al AAS. El tratamiento con clopidogrel está indicado en casos de alergia o intolerancia al AAS moderada	Moderada	Fuerte a favor
Doble antiagregación en pacientes coronarios estables sometidos a ICP electiva. Se recomienda AAS y clopidogrel durante un mes si se trata de un stent convencional (*bare metalstent*), y durante seis meses si se trata de un stent liberador de fármaco (*drug-eluting stent*). Después de este periodo se recomienda AAS de forma indefinida	Alta	Fuerte a favor
Doble antiagregación en pacientes que han padecido un SCASEST tratados mediante una ICP con stent liberador de fármaco. Se recomienda AAS 75-100 mg y un inhibidor del receptor plaquetario P2Y12 durante 12 meses:- ticagrelor 90 mg dos veces al día, en pacientes de moderado-alto riesgo isquémico- Prasugrel 10 mg, una dosis diaria en pacientes que van a proceder a la realizaciónde revascularización percutánea- clopidogrel 75 mg, una dosis diaria en pacientes que no pueden recibir ticagrelor oprasugrel, o que requieran anticoagulación oral adicional (triple terapia).	Alta	Fuerte a favor
Doble antiagregación en pacientes que han padecido un IAMCEST tratados medianteuna IPC con stent liberador de fármaco. Se recomienda AAS 75-100 mg y un inhibidordel receptor plaquetario P2Y12 durante 12 meses. En este caso se puede utilizarcualquiera de las opciones terapéuticas (clopidogrel, ticagrelor o prasugrel). En caso de necesitar anticoagulación oral adicional se debe utilizar clopidogrel	Alta	Fuerte a favor
Inhibidores de la bomba de protones. Se recomiendan en los pacientes tratados con doble antigregación y con un riesgo alto de padecer una hemorragia gastrointestinal	Alta	Fuerte a favor
Triple terapia (anticoagulante y doble antiagregación). Se recomienda en los pacientes con firme indicación de anticoagulación oral (como la HA) durante un tiempo limitado, que dependerá del riesgo de hemorragia	Alta	Fuerte a favor

AAS: ácido acetilsalicílico; FA: fibrilación auricular; IAMCEST: infarto agudo de miocardio con elevación del ST; ICP: intervención coronaria percutánea; SCASEST: síndrome coronario agudo sin elevación del ST.

Elaboración propia.

## Fibrilación auricular

### Importancia epidemiológica y clínica

La FA es una taquicardia supraventricular con una activación atrial eléctrica no coordinada que resulta en una contracción atrial ineficiente. La FA puede ser de dos tipos, clínica (con electrocardiograma [ECG] que documente la FA, con o sin síntomas) o subclínica (sin síntomas atribuibles a la FA y sin FA en el ECG previamente detectada). Esta última incluye los episodios auriculares de alta frecuencia (AHRE)^365^. En muchas ocasiones, la arritmia es silente o episódica y tarda tiempo en diagnosticarse, de modo que en muchos casos se reconocen de forma casual en pacientes asintomáticos o con escasos e inespecíficos síntomas^366^.

La FA afecta al 2-4% de la población general^5^, ^365^. Se ha estimado, a través de un amplio estudio poblacional de la Sociedad Española de Cardiología^367^, que la prevalencia de FA en la población española mayor de 40 años es de 4,4%, lo que supone que unos 2 millones de personas en España padece FA en sus distintas formas clínicas.

Se estima que el riesgo de por vida actual de padecer FA en la población europea es de 1 de cada 3 en individuos mayores de 55 años^5^. En el 2019, la incidencia de FA en Europa fue de 110,18 casos por cada 100.000 habitantes^368^, y que la incidencia se duplique hasta el año 2060, llegando a 17,9 millones de casos^369^, debido al aumento en su diagnóstico y al envejecimiento de la población^5^. Por otra parte, la FA subclínica o AHRE son frecuentes en pacientes con marcapasos, con una incidencia del 30% al 70%, aunque puede ser inferior en la población general^365^.

La FA constituye un factor de riesgo en el desarrollo de enfermedad renal, EV y de mortalidad^5^. La mortalidad por FA ha aumentado del 0,25% en 1990 al 0,56% en 2019^368^. La incidencia, prevalencia y riesgo de padecer FA es mayor en hombres que en mujeres al ajustar por edad^5^. Sin embargo, la mortalidad por FA es mayor en mujeres (0,75%) que en hombres (0,4%)^368^. Además, los episodios de 5-6 minutos de AHRE/FA subclínica se asocian a un incremento de riesgo de FA clínica, ictus isquémico, eventos vasculares mayores y muerte cardiovascular^365^. La FA clínica se desarrolla en uno de cinco a seis pacientes con FA subclínica, alrededor de 2,5 años tras su diagnóstico^365^.

La complicación más grave de la FA es el ictus, aumentando en cinco veces el riesgo de padecer un ictus; sin embargo, los ictus relacionados con la FA son en gran medida potencialmente prevenibles con tratamiento anticoagulante^5^, ^365^.

### Cribado y diagnóstico

El diagnóstico definitivo de la FA se realiza mediante ECG, sea con una tira ≥ 30 segundos con una derivación, o con un ECG de 12 derivaciones que muestren un ritmo cardiaco con ondas P repetidas no discernibles e intervalos RR irregulares (nivel de evidencia I-B)^365^.

Las estrategias de cribado de la FA usadas más frecuentemente son el cribado oportunista y el cribado sistemático de individuos ≥ 65 años o con otras características que se asocian con un aumento del riesgo de ictus, operativizados mediante trazados de ECG o la toma de pulso^5^.

#### Beneficios y riesgos del cribado

La Sociedad Europea de Cardiología argumenta que el cribado de la FA podría prevenir episodios de ictus y el inicio de los síntomas^365^. De la misma manera, podría prevenir el remodelado eléctrico/mecánico atrial, la aparición de trastornos hemodinámicos, cardiomiopatía y taquicardia atrial/ventricular, así como la morbilidad asociada a FA, hospitalizaciones y mortalidad. Por ello, se ha planteado que la detección de la FA permitiría instaurar tratamiento anticoagulante en los pacientes con riesgo embólico asociado según las recomendaciones de las guías^365^, ^370^, ^371^ y, de este modo, reducir las complicaciones tromboembólicas. En contraposición, el cribado podría causar ansiedad, sobrediagnósticos, sobretratamientos, así como el aumento en la realización de pruebas invasivas^365^.

#### Evidencia científica sobre el cribado de fibrilación auricular

La evidencia científica sobre el cribado de FA está constituida por ensayos clínicos aleatorios (ECA) que comparan estrategias de cribado (oportunista o sistemática) entre sí o con la práctica clínica habitual, y de estudios sobre métodos diagnósticos para la detección temprana de la FA (p. ej., a través de relojes inteligentes).

Existen diferentes herramientas para diagnosticar tempranamente la FA en pacientes asintomáticos, como lo son los ECG de una o 12 derivaciones, los dispositivos móviles, o los relojes inteligentes con algoritmos de detección de FA y a través de fotopletismografía. Al tener cada vez más dispositivos con alto potencial para detectar la FA de forma autónoma, la evidencia actual estudia la eficiencia de estos dispositivos y se plantea su uso dentro de las estrategias de cribado. Todavía no existe un consenso ni una estrategia de cribado determinada hasta el momento, por lo que es importante valorar la evidencia existente para valorar su aplicación.

A continuación se resumen de forma no exhaustiva algunos ensayos clínicos sobre estrategias de cribado y sus resultados principales.

- SAFE^372^: ECA multicéntrico realizado en el Reino Unido en 50 centros de AP, en pacientes de > 65 años y en tres brazos: a) grupo control (práctica clínica habitual); b) grupo de intervención mediante cribado oportunista (toma de pulso y ECG si el pulso era irregular), y c) cribado sistemático (ECG de 12 derivaciones). Se detectaron más personas con FA a través del cribado que con la práctica clínica habitual (un 64% más). En cambio, no se apreciaron diferencias en la detección de FA nueva entre el cribado sistemático y el oportunista.
- DOFA-AP^373^: ECA multicéntrico realizado en AP en España en persona > 65 años comparó el cribado oportunista (palpación de pulso y ECG si pulso irregular) con la búsqueda activa y selectiva de pacientes sintomáticos. Esta última resultó ser más efectiva en la detección de FA que el cribado oportunista.
- STROKESTOP^374^: ECA multicéntrico realizado en el 2021 en Suecia comparando el cribado sistemático con ECG intermitentes por 14 días que se focalizó en pacientes de 75-76 años, encontrando un leve beneficio del cribado sistemático con ECG de una derivación frente a la práctica habitual.
- Cluster ECA en los Países Bajos^375^: se evaluó si el cribado oportunista de FA mediante ECG portátil de una derivación en > 65 años. No se detectaron más casos con el cribado oportunista frente a la práctica clínica habitual.
- VITAL AF^376^: clúster ECA realizado en EE. UU. en personas > 65 años, que comparó el cribado de la FA con ECG con la práctica clínica habitual. No se encontró un beneficio neto en cuanto a la detección de nuevos casos de FA.
- Un meta-análisis de 25 estudios realizados en 14 países concluyó que ambos cribados tanto oportunista como sistemático fueron efectivos a partir de los 40 años, siendo más eficaz el cribado sistemático. Además, se observó que la diferencia entre la detección de FA mediante ECG fue mínimamente superior respecto a la toma del pulso^372^.

#### Recomendaciones de cribado de las Guías de Práctica Clínica

La Sociedad Europea de Cardiología recomienda la detección oportunista de FA mediante la toma del pulso o con ECG en pacientes > 65 años de edad^365^. También recomiendan interrogar periódicamente a los marcapasos y desfibriladores automáticos implantables por AHRE.

Para ello, se recomienda que:

- Se informe a las personas sobre la importancia y las implicaciones del tratamiento tras detectar la FA.
- Se realice una evaluación clínica adicional dirigida por un médico para confirmar el diagnóstico de FA y proporcionar un manejo óptimo de los pacientes con FA confirmada.
- El diagnóstico definitivo de FA se establece solo después de que el médico revise el registro de ECG de una sola derivación de > 30 s o ECG de 12 derivaciones y confirme la FA.

Por otra parte, se debería considerar la detección sistemática con ECG para detectar FA en personas mayores de 75 años o en aquellos con alto riesgo de accidente cerebrovascular^365^.

En cuanto a las guías americanas, la AHA reconoce que puede existir un papel en la detección de FA silenciosa mediante la adquisición y transmisión electrocardiográfica remota con un dispositivo «inteligente» portátil o portátil habilitado para wifi con interpretación remota^364^. En cuanto a la última recomendación por parte del *U.S. Preventive Service Task Force* en el 2022, se considera que la evidencia actual es insuficiente para determinar el balance riesgo/beneficios del cribado de FA en adultos de 50 años o más, asintomáticos, y sin historia previa de AIT o ictus (grado I - insuficiente)^376^.

### Tratamiento

Esta sección está basada en la Guía de Práctica Clínica de la Sociedad Europea de Cardiología^365^.

#### Estrategia terapéutica

El tratamiento anticoagulante forma parte de la estrategia *Atrial fibrillation Better Care* de atención integral de la FA, o vía ABC: A) anticoagulación y prevención del ictus, B) buen control de los síntomas, y C) control de los FRV y comorbilidades.

La implementación de esta vía se ha asociado con menor riesgo de muerte por cualquier causa, menor incidencia de la variable compuesta de ictus/sangrado mayor/muerte vascular y primera hospitalización, así como tasas más bajas de eventos vasculares y menos costes relacionados con la salud^365^.

#### Anticoagulación y prevención el ictus

La indicación del tratamiento anticoagulante se realiza sobre la base del riesgo embólico del paciente cuando se estima que este es superior al riesgo de hemorragia inherente a la propia anticoagulación. El riesgo embólico se estima con la escala CHA2DS2-VASc Score, y se recomienda la anticoagulación cuando puntúa ≥ 2 en los varones o ≥ 3 en las mujeres (nivel de evidencia I-A)^365^.

Para la prevención de eventos trombóticos en la FA se recomienda el uso de anticoagulación oral. Puede realizarse con agentes antivitamina K (AVK) como el acenocumarol y la warfarina, que tienen indicación en cualquier tipo de FA (valvular o no valvular), o bien con los nuevos anticoagulantes de acción directa (NACO) (dabigatrán, apixabán, rivaroxabán, edoxabán), que no precisan monitorización y han demostrado una similar eficacia a los agentes AVK con menos complicaciones hemorrágicas graves, como la hemorragia intracraneal^377^. Los AVK disminuyen el riesgo de ictus en un 64% y de mortalidad en un 26% comparado con placebo, mientras que los NACO demostraron una disminución del riesgo del 19% para ictus isquémico, del 51% para ictus hemorrágico, y del 10% de la mortalidad por cualquier causa en comparación con los AVK^365^.

Sin embargo, los NACO están contraindicados en pacientes con prótesis valvulares mecánicas o con estenosis mitral moderada a severa en los que se recomienda el uso de fármacos AVK. Cuando se administre un AVK, se recomienda una INR 2,0-3,0, con un tiempo en rango terapéutico (TRT) individual ≥ 70% (nivel de recomendación I-B). Si presentan un TRT < 70% se recomienda cambiar a un anticoagulante de acción directa siempre que se asegure una buena adherencia y la continuidad del tratamiento^365^.

En el embarazo, las recomendaciones del tratamiento de la FA se basan en la terapia anticoagulante con heparina o AVK, dependiendo del periodo de gestación, y el control de la frecuencia cardiaca se basa en la terapia con BB cardioselectivos (nivel de recomendación I-C)^365^.

#### Buen control de los síntomas

Se recomienda el uso de fármacos para el control del ritmo cardiaco como los BB, o diltiazem o verapamilo como fármacos de primera línea para el control de la frecuencia cardiaca de pacientes con FA y FEVI ≥40%. En casos de pacientes con FEVI < 40% se recomiendan los BB y la digoxina (ambas recomendaciones con nivel I-B)^365^.

#### Control de los factores de riesgo vascular y comorbilidades

La identificación y gestión de enfermedades concomitantes, factores de riesgo cardiometabólicos y factores de estilo de vida poco saludables complementa la prevención de accidentes cerebrovasculares y reduce la carga y gravedad de los síntomas de FA^365^.

Mientras que las estrategias de modificación integral de factores de riesgo y las intervenciones dirigidas a condiciones subyacentes han mostrado reducir la carga y recurrencia de FA, los estudios que abordan la gestión aislada de condiciones específicas (p. ej., hipertensión) dieron hallazgos inconsistentes, probablemente porque la condición no fue el único contribuyente a la FA^365^.

Por tanto, se recomienda la identificación y el tratamiento de los factores de riesgo y de las enfermedades concomitantes como parte integral del tratamiento en pacientes con FA (nivel de recomendación I-B)^365^. De la misma manera, se recomienda modificar el estilo de vida poco saludable y la terapia dirigida de enfermedades intercurrentes para reducir la carga de FA y la gravedad de los síntomas (nivel de recomendación I-B)^365^. Además, en los pacientes hipertensos con FA, se recomienda prestar atención a un buen control de la PA para reducir las recurrencias de FA y el riesgo de accidente cerebrovascular y hemorragia (nivel de recomendación I-B)^365^.

Por otra parte, se recomienda también la reducción de peso en pacientes obesos, evitar el exceso de alcohol en pacientes anticoagulados y realizar ejercicio moderado (nivel de recomendación II-A)^365^.

### Conclusiones

Aunque la evidencia científica es limitada, parece razonablemente plausible que una mayor detección de la FA conlleve un beneficio clínico significativo a medio y largo plazo. La toma del pulso está incluida en cualquier examen clínico básico, tanto en la AP como en el ámbito hospitalario. Por tanto, y de cara a un método más de cribado clínico inicial de FA, no parece probable atribuir costes adicionales a lo que se debería hacer de forma rutinaria en situaciones muy comunes en la práctica clínica ordinaria.

La guía del diagnóstico y tratamiento de la Sociedad Europea de Cardiología actualmente vigente recomienda el cribado de la FA por su bajo coste y potencial beneficio clínico debido a la magnitud del problema y de sus consecuencias y sus bien documentadas posibilidades de prevención mediante anticoagulación oral. Ahora bien, no existe un consenso sobre la realización del cribado en AP, por lo que, como norma general, se recomienda seguir la guía europea actual, teniendo en cuenta las diferencias entre recomendaciones de otras guías y la evidencia existente sobre el tema.

## Nuevas patologías y riesgo vascular

### Esteatosis hepática metabólica

La enfermedad de hígado graso no alcohólica/*non-alcoholic fatty liver disease* (NAFLD)/ esteatohepatitis no alcohólica (NASH) se asocia no solo con morbilidad y mortalidad hepática sino también con un mayor RV. El término MAFLD se propuso como un posible sustituto de NAFLD por consenso internacional. La NAFLD y las EV comparten varios factores de riesgo, como la obesidad, la RI, el síndrome metabólico, la hipertensión, la dislipidemia, la DM2 y la ERC. Las causas más comunes de muerte en pacientes con NAFLD en general son EV y cáncer no hepático, seguido de enfermedad hepática. Existe una fuerte asociación entre NAFLD y enfermedad cardiaca aterosclerótica, insuficiencia cardiaca y arritmias, particularmente FA. Optimizar la gestión de los FRV con el objetivo de reducir la morbilidad y mortalidad por EV es fundamental para mejorar los resultados en pacientes con NAFLD^378^, ^379^.

La evidencia reciente revela que la prevalencia global de NAFLD en adultos se estima en un 30% y > 50% de los pacientes con DM2, ≈70% entre los pacientes con EV y > 90% de los pacientes con obesidad grave. Las interrelaciones entre estas patologías respaldan el concepto de un entorno dismetabólico compartido impulsado por la genética, la epigenética, el desequilibrio entre la ingesta y el gasto energético y el estilo de vida. También hay evidencia de una fuerte asociación entre NAFLD/NASH y eventos vasculares mayores independientemente de los FRV tradicionales. Múltiples estudios han demostrado una asociación entre el desarrollo de NAFLD y una dieta subóptima, la inactividad física y el tabaquismo^379^, ^380^.

Generalmente se acepta que se deben utilizar métodos no invasivos para estratificar el riesgo de fibrosis avanzada. La prueba FIB-4 estima el grado de fibrosis hepática y permite clasificar correctamente al 87% de los pacientes con fibrosis hepática avanzada y así evitar la biopsia con un 71% de exactitud. Un FIB-4 < 1,30 indica que no hay fibrosis avanzada (F0-F1); un FIB-4 entre 1,30 y 2,67 requiere la prueba diagnóstica conocida como Fibroscan®; un FIB-4 > 2,67 indica fibrosis significativa (F3-F4). En pacientes con diagnóstico de NAFLD, esteatohepatitis no alcohólica (NASH) o riesgo de NASH y sin EV previa, un FIB-4 ≥ 2,67 es un fuerte predictor de MACE (HR ajustado [aHR] 1,80, IC 95% 1,61-2,02, p < 0,001), asociándose consistentemente con IAM (aHR 1,46, IC 95% 1,25-1,70, p < 0,001), hospitalización por angina inestable (aHR 1,24, IC 95% 1,03-1,49, p = 0,025), hospitalización por insuficiencia cardiaca (aHR 2,09, IC 95% 1,86-2,35, p < 0,001), injerto de derivación de arteria coronaria (aHR 1,65, IC 95% 1,26-2,17, p < 0,001) e ICP (aHR 1,72, IC 95% 1,21-2,45, p = 0,003)^381^.

**Figura 8:**
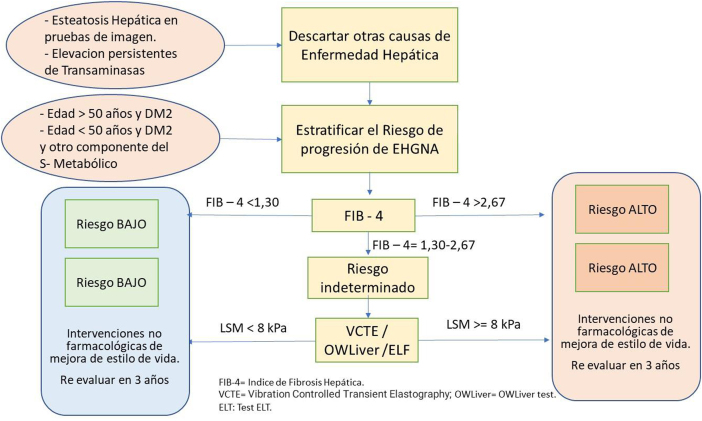
*Non-Alcoholic Fatty Liver Disease* (NAFLD) / *Non-Alcoholic SteatoHepatitis* (NASH). Algoritmo de actuación y riesgos según el índice FIB de fibrosis hepática. Modificado de ^362^.

En pacientes con prediabetes, DM2 o dos o más factores de riesgo metabólicos (o evidencia por imágenes de esteatosis hepática), la evaluación primaria del riesgo con FIB-4 debe repetirse cada uno o dos años. Si la FIB-4 es ≥ 1,3, se pueden utilizar *Vibration Controlled Transient Elastography* (VCTE), *Magnétic Resonance Elastography* (MRE); o el Test ELF, un análisis de sangre no invasivo que mide tres marcadores directos de fibrosis: ácido hialurónico, péptido amino terminal del procolágeno tipo III e inhibidor tisular de la metaloproteinasa de matriz 1, para excluir la fibrosis avanzada. Ver guía de la American *Association for the Study of Liver Diseases*, AASLD, 2023)^382^. La detección precoz del grado de fibrosis hepática en Atención Primaria a pacientes con NAFLD pueden constituir una opción de cribado oportunista previa a la confirmación mediante elastografía de transición (Fibroscan®), prueba diagnóstica que cuantifica la rigidez del hígado que es proporcional al grado de fibrosis hepática. La detección en dos pasos combinando los métodos no invasivos bioquímicos con la elastografía transitoria constituye el algoritmo de elección: paso 1 (Atención primaria): FIB-4 (y test ELF si FIB-4 no es preciso); paso 2 (Medicina Digestiva): Fibroscan^379^, ^380^ (fig. 8).

## Recomendaciones NO HACER

### No hacer en hipertensión arterial

- Medir la PA sin seguir las recomendaciones consensuadas (manguitos adecuados, aparatos validados, reposo previo, postura adecuada, etc.).
- Realizar una sola medida para el diagnóstico de HTA, y no realizar tomas fuera de consulta para confirmar la misma (MAPA o AMPA).
- Consumir con frecuencia alimentos salados (p. ej., pan y bollos, queso, hamburguesas con queso, carnes frías como jamón o pavo, pizza o platos de pasta, sopas, aperitivos como patatas fritas), y ultra-procesados.
- Tratar a pacientes mayores únicamente por su edad, o considerar objetivos terapéuticos más bajos basándose únicamente en la misma.
- Considerar que las personas mayores frágiles posiblemente se puedan beneficiar de una PA más alta y, por tanto no tratar, sin reevaluar periódicamente la posibilidad de tratamiento farmacológico.
- Iniciar un tratamiento farmacológico antihipertensivo en todas las elevaciones agudas de la PA.
- Iniciar un tratamiento farmacológico con monoterapia usando dosis bajas de hipotensores (habituales en insuficiencia cardiaca).
- Usar dos fármacos de inicio en HTA grado 1 de bajo o moderado riesgo, o cuando el objetivo terapéutico sea inferior a 20/10 mmHg del inicial.
- Asociar IECA con ARA 2, o BB con calcio-antagonistas no dihidropiridínicos.
- Utilizar diuréticos de asa en el tratamiento de la HTA esencial.
- No tratar con IECA, ARAII, inhibidores de la renina, inhibidores de la neprilisina o antagonistas de la aldosterona en pacientes hipertensas y embarazadas o que contemplen embarazo^33^, ^383^.

### No hacer en dislipemia

- En pacientes con insuficiencia cardiaca no se recomienda iniciar tratamiento hipolipemiante en adultos que no tengan otra indicación para su uso; tampoco en pacientes con estenosis valvular aórtica (para reducir su progresión) sin enfermedad coronaria. Por otra parte, las enfermedades inflamatorias crónicas inmunomediadas (CIIM), por sí solas, no constituyen una indicación para el inicio de estatinas^384^.
- No tratar con estatinas en adultos con las siguientes condiciones o patologías:
  ∘ Dislipemia con creatina quinasa (CK) cinco veces por encima del límite superior, medida dos veces en una semana^385^.
  ∘ Dislipemia en pacientes con ERC en diálisis y sin EV aterosclerótica^385^.
  ∘ Dislipemia en mujeres durante el embarazo y periembarazo^384^, ^385^.
  ∘ Dislipemia en mujeres en periodo de lactancia^385^.

### No hacer en obesidad

- Prescripción de dieta no adaptada a las preferencias del paciente.
- Prescripción de ejercicio físico no adaptado al paciente.
- Prescripción de tratamiento farmacológico sin abordaje de terapia conductual, dieta y ejercicio físico.
- Derivación a CB a pacientes con IMC ≥ 35 y < 40 kg/m^2^ sin complicaciones ni patología asociada.
- Derivación a CB a pacientes con IMC 30-34,9 kg/m^2^ donde la pérdida ponderal no sea prioritaria.

### No hacer en diabetes

- Hacer cribado poblacional de diabetes. Debe hacerse cribado en población de riesgo mediante glucemia basal o HbA1c y preferiblemente aprovechando una analítica por otro motivo.
- Obviar el uso de iSGLT2 o arGLP1 en pacientes en prevención secundaria vascular o con ERC.
- Obviar el uso de iSGLT2 en pacientes con DM e insuficiencia cardiaca.
- Esperar a intensificar el tratamiento cuando un paciente no presenta buen control o porque presenta cifras límite. Hay que evitar la inercia.
- No medir la adherencia terapéutica de forma habitual. Antes de intensificar un tratamiento hay que comprobar si el paciente lo toma.
- Usar fármacos que producen hipoglucemias sin probar alternativas farmacológicas más seguras.
- Circunscribir las visitas de revisión a la medición del peso y la glucemia capilar sin plantear objetivos consensuados y estrategias de educación sanitaria para conseguirlos.
- Iniciar el tratamiento insulínico con 10 unidades y decirle al paciente que suba dos o tres unidades cada tres días, sin advertir al paciente cuál es la dosis habitual.
- Permitir en el momento del diagnóstico y meses siguientes controles laxos en lugar de buscar un control lo mejor posible para alcanzar el efecto legado.
- No medir de forma conjunta el FG y el CAC. Pues los dos parámetros son necesarios para caracterizar el grado de afectación renal.
- Prescribir fármacos antidiabéticos sin conocer la función renal que puede condicionar el uso de algunos de ellos o la dosificación.
- No comprobar en cada visita si el peso del paciente ha quedado registrado en la historia.
- Prescribir un iDPP4 junto con un arGLP1.
- Prescribir fármacos que precisan varias tomas al día frente a los de toma única o terapias combinadas que facilitan la adherencia terapéutica.

### No hacer en enfermedad renal crónica

- No determinar el IAC en el cribado y diagnóstico de la ERC.
- No registrar el diagnóstico y estadio de la ERC.
- No hacer un manejo integral de la ERC.
- No utilizar metformina (ajustada al grado de ERC), asociada a iSGLT2 como tratamiento farmacológico de primera línea de la DM2 en la ERC.

### No hacer en antiagregación

- Usar AAS como prevención primaria en personas sin EV.
- Usar como primera línea de tratamiento clopidogrel en monoterapia tras un IAM.
- Usar anticoagulantes de forma rutinaria en el tratamiento del ictus agudo.

### No hacer en fibrilación auricular

Las recomendaciones de no hacer de la Guía de Práctica Clínica de la Sociedad Europea de Cardiología^365^, Colegio Americano de Cardiología^386^:

- No se recomienda el tratamiento antiplaquetario solo (monoterapia o AAS en combinación con clopidogrel) para la prevención del accidente cerebrovascular en la FA (nivel de evidencia III-A). Es necesario identificar y tratar otros factores de riesgo y enfermedades concomitantes.
- El riesgo estimado de hemorragia, en ausencia de contraindicaciones absolutas para la anticoagulantes orales (ACO), no debería por sí solo guiar las decisiones de tratamiento para usar ACO en la prevención del accidente cerebrovascular (nivel de evidencia III-A).
- El patrón clínico de FA (es decir, detectada por primera vez, paroxística, persistente, persistente de larga duración, permanente) no debería condicionar la indicación de tromboprofilaxis (nivel de evidencia III-A).
- Los NACO están contraindicados en pacientes con prótesis valvular mecánica (nivel de evidencia III-B).
- No se recomienda el uso de NACO en pacientes con FA y estenosis mitral moderada a grave (nivel de evidencia III-C).
- En pacientes con síndrome del seno enfermo, alteraciones de la conducción auriculoventricular o QTc prolongado (> 500 ms), no se debería intentar la cardioversión farmacológica a menos que se hayan considerado los riesgos de proarritmia y bradicardia (nivel de evidencia III-C).
- No se recomienda el tratamiento con fármacos antiarrítmicos en pacientes con FA permanente con control de frecuencia y en pacientes con alteraciones avanzadas de la conducción, a menos que se proporcione estimulación antibradicardia (nivel de evidencia III-C).
- Los BB no deben utilizarse de forma rutinaria para la prevención de la FA postoperatoria en pacientes sometidos a cirugía no cardiaca (nivel de evidencia III-B).
- No se recomienda la realización de ablación de FA con el único fin de suspender la anticoagulación crónica. No se ha demostrado que la anticoagulación se pueda suspender de manera segura después de la ablación en pacientes con riesgo elevado de accidente cerebrovascular.

## Abordaje de la cronicidad desde Atención Primaria

El paciente con factores de riesgo o patología vascular es un paciente con una condición crónica de salud que requiere un enfoque diferente al tradicional aportado por el sistema sanitario para el abordaje de las patologías agudas. Esta orientación hacia la atención episódica y aguda, no permite hacer frente a las necesidades multifacéticas y complejas de las personas con enfermedades crónicas, y los pacientes a menudo reciben una atención con una participación limitada en el manejo de la enfermedad y escasa coordinación y comunicación entre los proveedores de la atención. Algunas de las características diferenciales se describen en la tabla 16. Se trata de factores de riesgo (HTA, DM2, dislipemia [DLP]) que suelen cursar de forma asintomática por lo que el paciente precisa una buena motivación para tomar medicamentos toda la vida. Por otro lado, la mayoría de medicamentos son preventivos y por tanto el paciente no percibe una mejoría por tomarlos (estatinas, anticoagulantes), por lo que la falta de adherencia es uno de los principales problemas para conseguir un buen grado de control. Los estudios demuestran que el grado de control por ejemplo de la glucemia en el caso de la DM2 se sitúa alrededor del 50%, muy bajo^387^, ^388^, ^389^.

**Tabla 16:** Diferencias en las necesidades de atención sanitaria entre pacientes con patologías agudas o crónicas

	Agudas	Crónicas
Necesidad de seguimiento en el tiempo	Baja (limitado)	Alta (prolongado)
Tratamiento	Agudo (curativo)	Crónico (preventivo)
Síntomas	Sí	No
Necesidad de educación sanitaria	Baja	Alta
Necesidad de colaboración y compromiso del paciente	Baja	Alta
Comorbilidades asociadas	Raro	Frecuente
Problemas de adherencia	Bajos	Altos
Número de fármacos	Bajo	Alto (polimedicación)
Edad	Variable	Media/alta
Necesidad de cuidador	Raro	Frecuente

Elaboración propia.

En HTA o DLP los datos son similares^4^, ^289^, ^290^. En un estudio con 92.436 pacientes en prevención secundaria en la Comunidad Valenciana, se observó que al año después del evento solo el 43% de los pacientes seguía tomando los tres fármacos profilácticos, IECA/ARA2, estatina y ASS^390^ comprobando, asimismo, en un seguimiento de tres años, que la mortalidad en los pacientes con buena adherencia era un 39% menor (HR 0,61, IC 95% 0,51-0,74) respecto a los pacientes que habían dejado de tomar estos fármacos. Es decir, la adherencia constituye uno de los pilares fundamentales en el seguimiento de estos pacientes, pues es la primera causa de mal control. Antes de intensificar un fármaco o cambiarlo hay que valorar si el paciente lo estaba tomando correctamente. Además, se ha comprobado que el médico suele sobreestimar la adherencia de sus pacientes cuando no se mide objetivamente^391^.

En la tabla 17 se describen de menor a mayor complejidad, tres métodos para valorar la falta de adherencia: La persistencia o retirada de la prescripción en la farmacia^231^, la pregunta de Haynes et al.^392^, y el recuento de comprimidos^313^. No se incluye la escala Morisky Medical Adherence Scale (MMAS) 8 por no haber alcanzado buenos indicadores para su uso en la validación realizada en español^393^.

**Tabla 17:** Instrumentos de medida para valorar la adherencia terapéutica utilizando la diabetes como ejemplo

Instrumento de medida	Pregunta	Respuesta
Revisión de la historia de salud electrónica	¿Está registrada la dispensación del fármaco en la farmacia al paciente de forma continuada?	Sí (el paciente ha retirado el fármaco de la farmacia; se considera buena adherencia) No (el paciente ha dejado de retirar el fármaco. Se considera abandono terapéutico)
Preguntas de Haynes Sacckett	La mayoría de los pacientes tienen dificultades en tomar todos sus comprimidos para la diabetes. ¿Tiene usted dificultades en tomar todos los suyos? Si la respuesta es afirmativa se pregunta por la toma de comprimidos en el último mes aplicando los mismos criterios que en el caso del recuento de comprimidos	Sí (el paciente comunica mala adherencia) No (el paciente comunica buena adherencia)
Recuento de comprimidos	Recuento de comprimidos consumidos en relación con los que deberían haber sido consumidos en un periodo de tiempo (p. ej., 1 mes) Se considera buena adherencia el consumo del 80-110% de los comprimidos prescritos en un periodo de tiempo (p. ej., 1 mes)	< 80% 80-110% Más de 110%

Elaboración propia.

La medición de la persistencia permite identificar el abandono terapéutico observando en la historia clínica electrónica si la farmacia ha dejado de dispensar el fármaco. Es el primer método a valorar, pues si el paciente no está retirando la medicación de la farmacia no requiere otros métodos de medición alternativos. A la pregunta de Haynes et al.^392^, comentar que muestra una alta especificidad (pocos falsos positivos) pero baja sensibilidad (muchos falsos negativos) lo que significa que si el paciente contesta que tiene dificultades para tomar la medicación puede identificarse la falta de adherencia con poca probabilidad de error; pero si el paciente contesta que no tiene dificultades debe emplearse otro método complementario (recuento de comprimidos), antes de identificar al paciente como adherente a la medicación.

En el artículo de García et al.^313^ se describen todos los métodos. Una sencilla estrategia es conseguir, al menos una vez al año, y preferiblemente en la consulta de enfermería, que el paciente traiga la medicación que tiene en casa y comente cómo toma y para qué toma cada fármaco. En una revisión sistemática analizando intervenciones para mejorar la adherencia terapéutica, se describen tres intervenciones que consiguieron mejoras tanto en la adherencia como en los resultados clínicos: los mensajes cortos por telemedicina (65% vs. 13%), el uso de terapias combinadas a dosis fija (86% vs. 65%) y una intervención basada en trabajadores sanitarios comunitarios (OR = 2,62; IC 95%: 1,32 a 5,19)^394^.

Otra de las principales causas de la falta de control es la inercia clínica, tanto diagnóstica como terapéutica. La inercia diagnóstica ha sido acuñada por el Centro de Investigación en Atención Primaria de la Universidad Miguel Hernandez^395^, ^396^, ^397^, ^398^ y hace referencia a la existencia en los registros de la historia clínica de valores diagnósticos; pero ausencia de registro diagnóstico ni intervención terapéutica (p. ej., dos o más registros de glucemia superiores a 126 mg/dL sin diagnóstico de diabetes ni tratamiento). La inercia clínica fue definida por Phillips et al. como los fallos del médico en la iniciación o intensificación del tratamiento cuando están indicados^399^. Aunque el término de inercia terapéutica fue nombrado por primera vez por Andrade et al.^400^.

En España, diversos estudios han puesto de manifiesto la importancia de evitar la inercia para conseguir un buen control, observando inercia en uno de cada cinco pacientes con diabetes^321^, mientras en otro estudio afectaba a uno de cada tres pacientes^401^. En un estudio analizando de forma conjunta adherencia e inercia en 3900 pacientes con HTA mal controlada, se observó que en uno de cada dos la causa era falta de adherencia y en uno de cada tres la causa era la inercia terapéutica^402^.

La falta de adherencia junto con la inercia son las dos causas principales de no alcanzar un buen control de los factores de riesgo cardiovascular para los que se dispone de eficaces tratamientos^225^, ^294^, ^403^.

**Figura 9:**
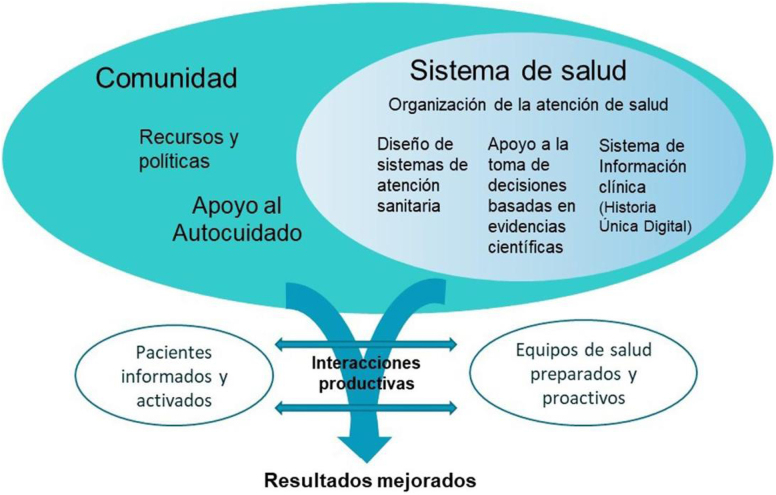
Modelo *Chronic Care Model* de atención integral a pacientes con problemas crónicos de salud. Tomado de: 404, 405.

**Figura 10:**
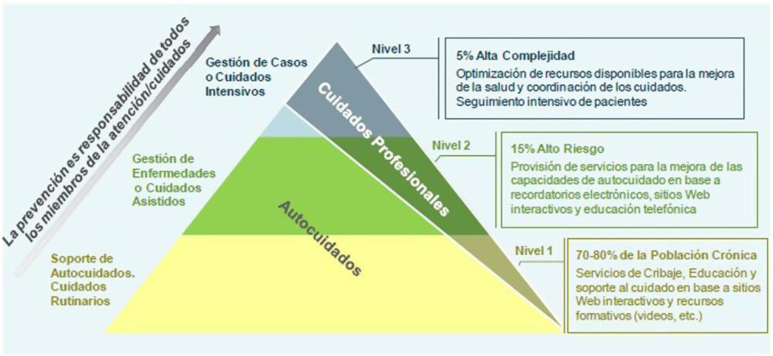
Modelo Kaiser Permanente de estratificación del riesgo de descompensación en pacientes crónicos. Tomado de 389, 390.

Dos modelos destacan a nivel internacional para mejorar la atención a los pacientes crónicos: El *Chronic Care Model* (CCM)^404^, ^405^ y el Káiser permanente (KP)^406^ (Figura 9, Figura 10).

El CCM incide en la necesidad de una corresponsabilidad entre el paciente y el profesional sanitario para alcanzar un buen control de las patologías crónicas. La guía de la *American Diabetes Association* (ADA) 2024 lo recomienda con un nivel alto de evidencia^407^.

Ello supone diseñar estrategias para conseguir un paciente informado y activo capacitado para el automanejo de la patología y un equipo de salud proactivo que tome medidas basadas en evidencias científicas y que utilice los sistemas de información para implementar una cultura evaluativa que permita medir resultados en salud. El KP recomienda estratificar el riesgo de descompensación de los pacientes. Actualmente la historia de salud electrónica en España dispone de medios automatizados para realizar en cada consulta listados de pacientes basados en riesgo aplicando diversos algoritmos (grupos de riesgo clínico (CRG) o grupos clínicos ajustados (ACG). A modo de esquema pueden resumirse las siguientes actuaciones que facilitarían la aplicación de estos modelos en la práctica clínica en AP para prestar una atención más integral a los pacientes crónicos con multimorbilidad^387^, ^388^, ^389^:

**Impulsar las actividades preventivas**. Actitud proactiva siguiendo las recomendaciones del PAPPS.
**Estratificar el riesgo del paciente**, siguiendo las recomendaciones del modelo de Káiser Permanente, permitirá identificar aquellos pacientes que requieren mayor atención por el profesional sanitario. Individualizar el tiempo de atención al paciente que más lo necesita.
**Favorecer la capacitación** (empoderamiento) del paciente y su entorno. Tomar decisiones compartidas con el paciente para hacerle corresponsable del manejo de la patología. Implicar al cuidador formal o informal. Favorecer la educación sanitaria.
**Valorar la actitud a través de las fases de cambio conductual,** del modelo de Prochaska y Di Clemente ayudará a ser más eficaces en las intervenciones siendo más intensivos en los pacientes más receptivos y a trabajar el cambio de fase en los menos receptivos, analizando las causas.
**Comprobar la adherencia terapéutica del paciente y la inercia clínica del profesional** de forma periódica y sistemática.
**Emplear la telemedicina** para la monitorización no presencial, pues ha demostrado ser una medida complementaria y eficaz a las intervenciones tradicionales.
**Trabajar en equipos multidisciplinares.** Una historia de salud electrónica única compartida entre profesionales pues la atención a estos pacientes requiere de equipos multidisciplinares donde el intercambio de información es imprescindible para garantizar la eficacia y la seguridad de las intervenciones.
**Implicar a la comunidad.** Realizar actividades que permitan promover la salud en la comunidad.
**Tomar decisiones basadas en la evidencia científica,** priorizando aquellas con mayor nivel de evidencias y **abandonando las prácticas que no aportan valor (no hacer).**
**Prestar atención integral e integrada abordando los aspectos biológicos, psicológicos y sociales.** Este es uno de los pilares básicos de la medicina de familia. Pero también es preciso conocer y disponer de recursos sociales para resolver problemas sociales en el entorno social y no en el sanitario.

## Financiación

La presente investigación no ha recibido ayudas específicas provenientes de agencias del sector público, sector comercial o entidades sin ánimo de lucro

## Conflicto de intereses

Los autores declaran no tener ningún conflicto de intereses.

## Colaboradores

Antonio Pérez Pérez. Especialista en Endocrinología. Hospital de Santa Creu i Sant Pau. Barcelona. Universidad Autónoma de Barcelona. CIBERDEM. Miguel Angel Sánchez-Chaparro. Especialista en Medicina Interna. UGC Medicina Interna, Hospital Universitario Virgen de la Victoria, Málaga. Alberto Cordero Fort. Especialista en Cardiología. Hospital Universitario San Juan de Alicante. Rafael Santamaría Olmo. Especialista en Nefrología. Hospital Universitario Reina Sofia. Investigador en el Instituto Maimónides de Investigación Biomédica de Córdoba (IMIBIC). Córdoba.
